# Measuring the effect of Non-Pharmaceutical Interventions (NPIs) on mobility during the COVID-19 pandemic using global mobility data

**DOI:** 10.1038/s41746-021-00451-2

**Published:** 2021-05-13

**Authors:** Berber T. Snoeijer, Mariska Burger, Shaoxiong Sun, Richard J. B. Dobson, Amos A. Folarin

**Affiliations:** 1 ClinLine, Leiderdorp, The Netherlands; 2 OCS Life Sciences, ‘s Hertogenbosch, The Netherlands; 3 The Department of Biostatistics and Health informatics, Institute of Psychiatry, Psychology and Neuroscience, King’s College London, London, UK; 4 Institute of Health Informatics, University College London, London, UK; 5 South London and Maudsley NHS Foundation Trust, London, UK

**Keywords:** Health care economics, Health policy

## Abstract

The implementation of governmental Non-Pharmaceutical Interventions (NPIs) has been the primary means of controlling the spread of the COVID-19 disease. One of the intended effects of these NPIs has been to reduce population mobility. Due to the huge costs of implementing these NPIs, it is essential to have a good understanding of their efficacy. Using aggregated mobility data per country, released by Apple and Google we investigated the proportional contribution of NPIs to the magnitude and rate of mobility changes at a multi-national level. NPIs with the greatest impact on the magnitude of mobility change were lockdown measures; declaring a state of emergency; closure of businesses and public services and school closures. NPIs with the greatest effect on the rate of mobility change were implementation of lockdown measures and limitation of public gatherings. As confirmed by chi-square and cluster analysis, separately recorded NPIs like school closure and closure of businesses and public services were closely correlated with each other, both in timing and occurrence. This suggests that the observed significant NPI effects are mixed with and amplified by their correlated NPI measures. We observed direct and similar effects of NPIs on both Apple and Google mobility data. In addition, although Apple and Google data were obtained by different methods they were strongly correlated indicating that they are reflecting overall mobility on a country level. The availability of this data provides an opportunity for governments to build timely, uniform and cost-effective mechanisms to monitor COVID-19 or future pandemic countermeasures.

## Introduction

The pandemic caused by the coronavirus disease 2019 (COVID-19) has had a huge impact on global health and economies, with costs of $11 trillion, projected (as of June 2020) by the International Monetary Fund (IMF)^1^. The implementation of governmental Non-Pharmaceutical Interventions (NPIs) has been the primary means of controlling the pandemic. NPIs have been issued by governments worldwide in response to the COVID-19 pandemic and collected by Assessment Capacities Project (ACAPS)^2^. ACAPS NPIs were standardised and included a secondary review and categorisation into five groups: social distancing, movement restrictions, public health measures, social and economic measures and lockdowns. These standardised NPIs and the corresponding methodology and data coding insights of ACAPS are freely available along with the actual interventions issued per country and the corresponding classifications^2^. The main parameters governing communication of infectious disease are duration of infectious period, infectiousness, susceptibility and opportunity. Of these parameters, opportunity is the most readily modulated without use of pharmaceuticals. It has been widely demonstrated in epidemiological studies that mobility is important in limiting opportunity for infection and several NPIs therefore target mobility. A strong reduction in mobility has a positive effect on reducing COVID-19 transmission by limiting opportunity for the virus to spread in the population and thus reducing the effective reproduction number “*R*_t_”. The positive effect of issuing NPIs on the reduction of COVID-19 has already been reported for different regions and countries like Wuhan^3^, China^4–6^, Hong Kong^7^, the UK^8^, Spain^9^ and Europe^10^. Due to the huge costs of implementing these NPIs, it is essential to have a good understanding of their benefits. It is plausible that NPIs with the same intention and thus classification may have different effects in different countries, as substantiated and reported by Haug et al.^11^ The corresponding variation in effectiveness can be approximated by assessing the change in mobility in each country by using freely available aggregated phone-derived mobility data from Apple and Google^12,13^. Apple mobility data reflect requests for directions in Apple Maps for driving, walking and train transit, while Google mobility data shows movement trends by country across different categories of places, like retail and recreation (RAR), grocery and pharmacy, transit and stations (TS), parks, workplaces and residential.

Though the intended positive effects of constrained mobility on disease reduction are clear, there are substantial negative consequences on wellbeing^14^, education^15^ and the world economy^1^. Therefore, it is important that the costs and benefits are carefully weighed in a timely manner and at a local level. A first step in this process is to establish appropriate mechanisms and metrics to measure the effects of NPIs efficiently. Countries/regions emerging from these measures may have a preference to shift to more regional or localised NPIs. Smartphone mobility data provides a real time, passive means to observe mobility at both population and individual levels.

Deriving meaningful insights from real world data, like Google and Apple mobility rates, is challenging. Weekday effects, holiday effects and effects of unexpected events vary from period to period and from country to country and are reflected in the data. Standard modelling techniques like deriving inflexion points from local regression (LOESS) of fit data is less useful for this kind of real world time series data. Using mobility and NPI data from several countries, this study aims to establish a useful model from which the proportional effects of the NPIs on the mobility state transition can be assessed between the period before the first significant drop in mobility and the relative stable period of low mobility after this first drop (further referred to as pre- and post-lockdown).

## Results

### Mobility per country

The visualisations of the mobility per country are available as Supplementary Fig. 1 “Apple mobility profiles per country”, Supplementary Fig. 2 “Google RAR mobility profiles per country” and Supplementary Fig. 3 “Google TS mobility profiles per country”. The Apple Mobility data was available for 59 countries and the Google data for 124 countries. Of these, we had data from both sources for 56 countries.

### Descriptive analysis of average % mobility lost after stabilizing

**Fig. 1:**
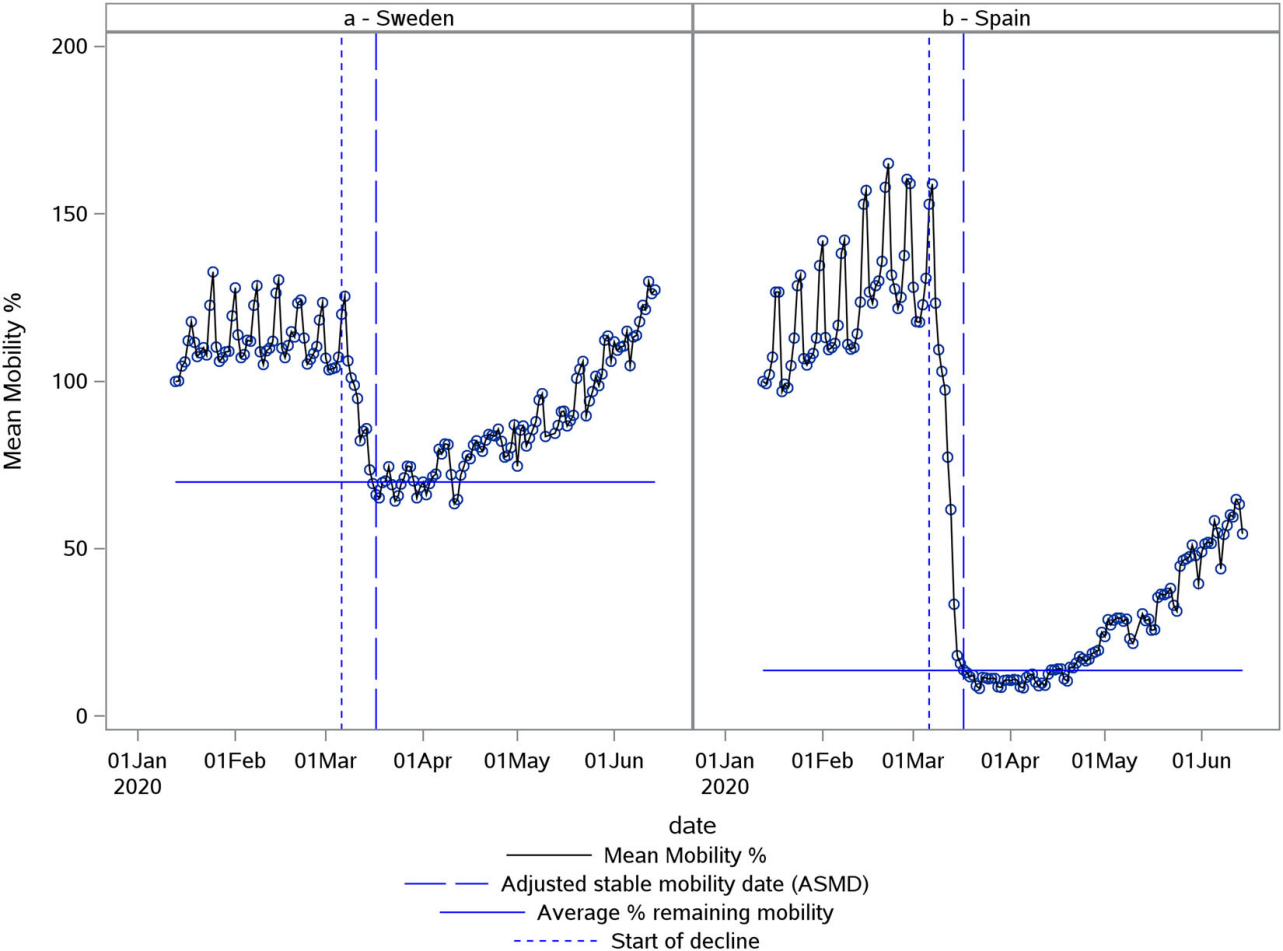
Largest and smallest % mobility lost. Plot of Apple normalized mobility data in % deviation from baseline. The smallest % mobility lost was observed for Sweden (**a**). The largest % mobility lost was observed for Spain (**b**). The first vertical (blue small) dotted line indicates the estimated start of decline. The second vertical (blue large) dotted line indicates the adjusted stable mobility date (ASMD). The horizontal line indicates the estimate for average % remaining mobility.

**Fig. 2:**
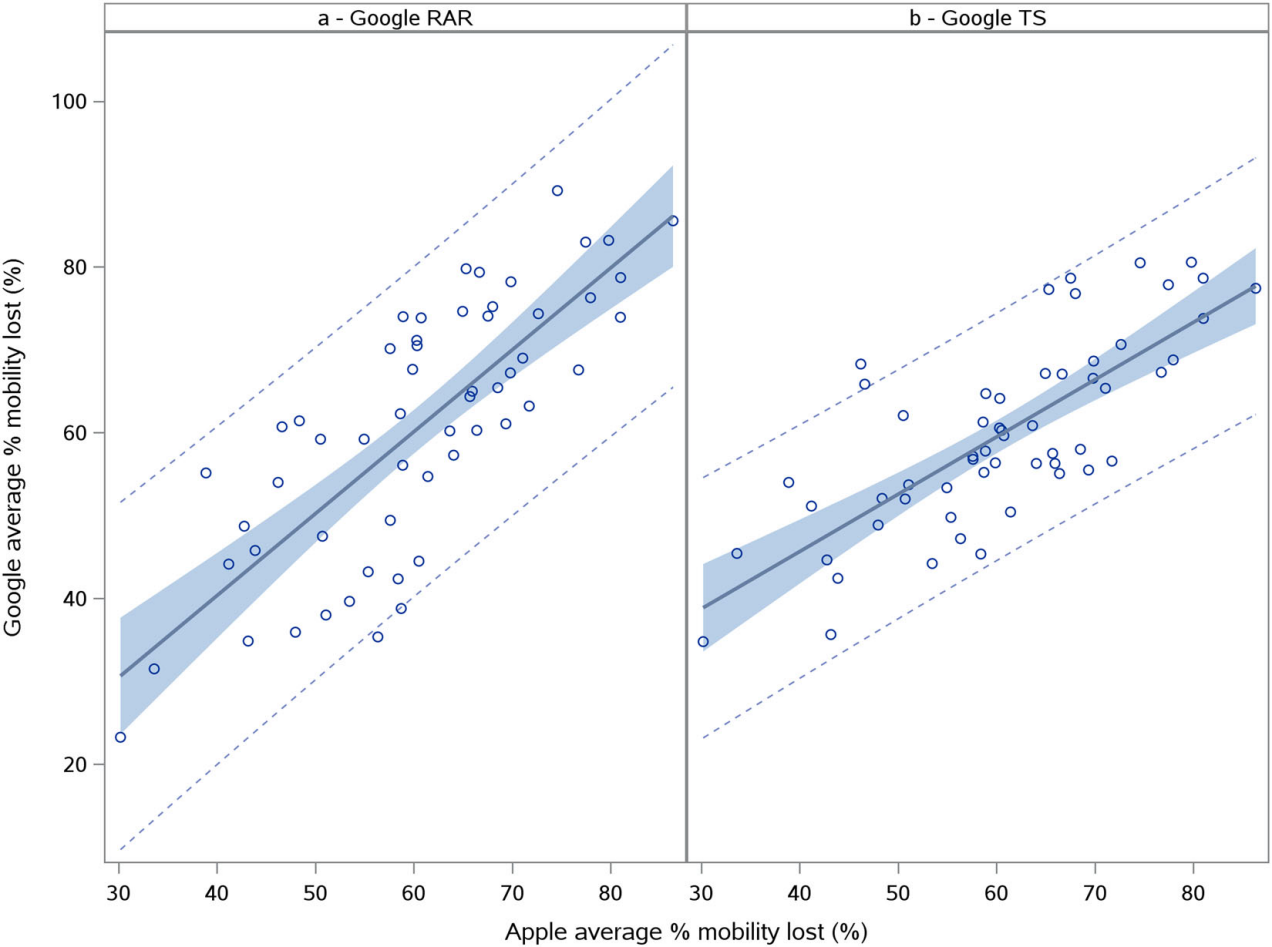
Correlation between Apple and Google mobility data for average % mobility lost. Plot of Apple % mobility lost vs Google RAR (**a**) and Google TS (**b**) % mobility lost after stabilizing including regression line (gray line) and corresponding 95% prediction (dotted line) and confidence limits (light blue area).

The average % mobility lost after stabilizing was estimated for all countries for which data was available. Only in 3 cases (2 for Google RAR and 1 for Google TS) this parameter could not be estimated based on the derivation method described above. This was due to too much variation in the mobility data and a less clear lockdown pattern. It resulted in a slightly lower *n* for the overall global summary statistics that were calculated based on this parameter for both Google RAR and Google TS data [Table 1]. The average % mobility lost for the Apple data follows a normal distribution with a mean of 60.6%. The smallest % mobility loss was observed for Sweden (30.2%), which did not have either a partial or full lockdown before the stabilizing of mobility. The largest % mobility loss was observed for Spain (86.3%) which had a partial lockdown before the stabilization of mobility [Fig. 1]. Likewise, both the Google RAR data as well as the Google TS data followed a normal distribution for this parameter with slightly lower mean values [Table 1]. The Pearson’s correlation between the estimated % mobility lost for Google RAR and Apple data was 0.7824 (*p* < 0.0001) and between Google TS and Apple data it was 0.7607 (*p* < 0.0001) [Fig. 2]. This indicates that both data sources independently show similar effects of the NPIs issued despite the different mobility measurement methods.

**Table 1:** Global summary statistics for the average % mobility lost after stabilizing.

Statistic	Apple (*N* = 59)	Google RAR (*N* = 124)	Google TS (*N* = 124)
*n*	59	122	123
Mean (SD)	60.6 (12.2)	56.9 (17.7)	57.2 (15.5)
Median	60.3	60.3	57.5
Range	30.2–86.3	19.8–89.2	20.4–86.5
95% CI	(57.4; 63.7)	(53.7; 60.1)	(54.4; 60.0)

*N* – Total number of countries.

*n* – Number of countries included in analysis.

SD – Standard deviation.

CI – Confidence interval.

### Descriptive analysis of the speed of effect

**Fig. 3:**
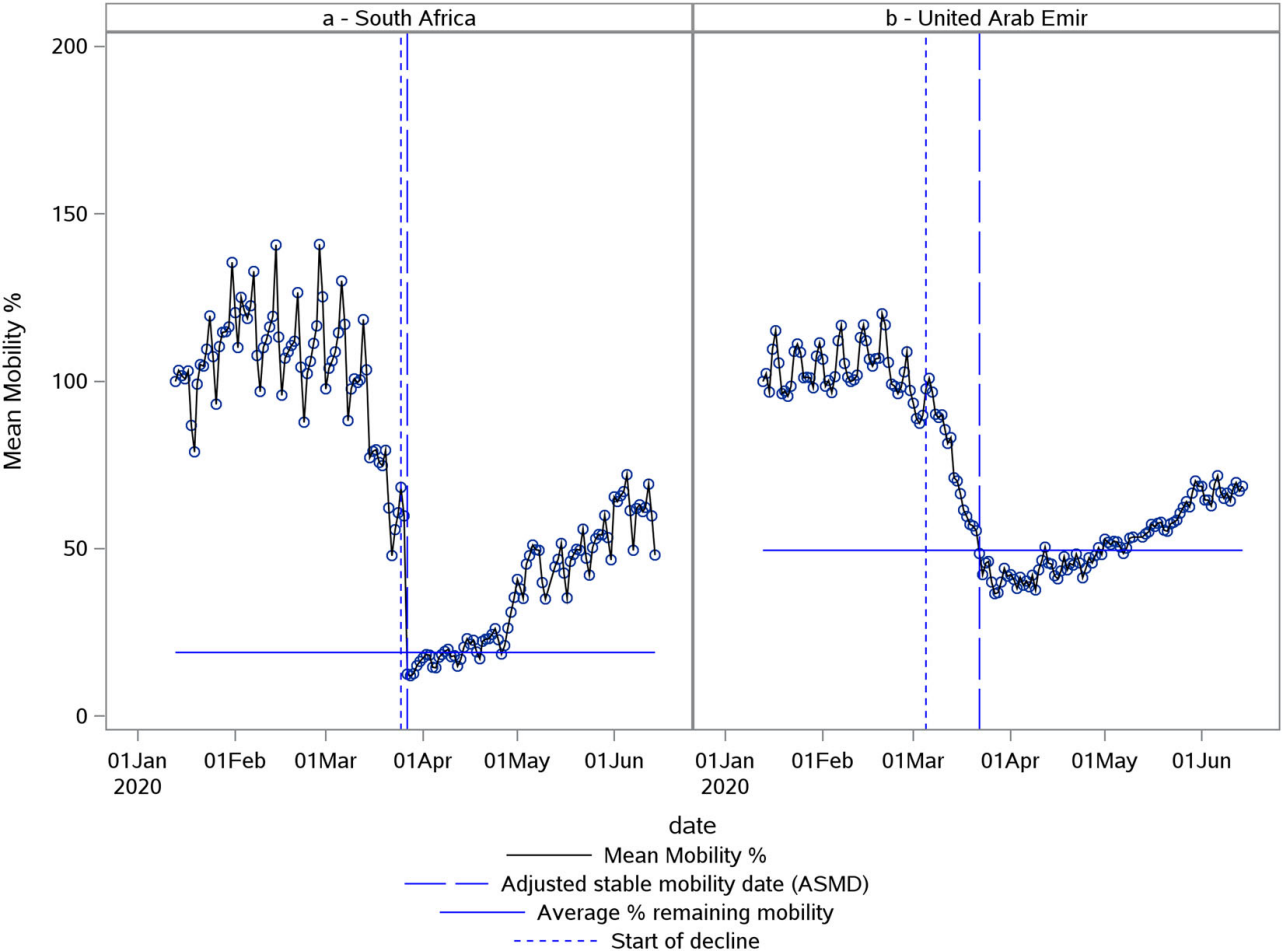
Comparison between fastest and slowest effect on mobility. Plot of Apple normalized mobility data in % deviation from baseline. The fastest effect of NPIs on mobility was observed for South-Africa (**a**) while the slowest effect was seen in the United Arab Emirates (**b**). The first vertical (blue small) dotted line indicates the estimated start of decline. The second vertical (blue large) dotted line indicates the adjusted stable mobility date (ASMD). The horizontal line indicates the estimate for average % remaining mobility.

**Fig. 4:**
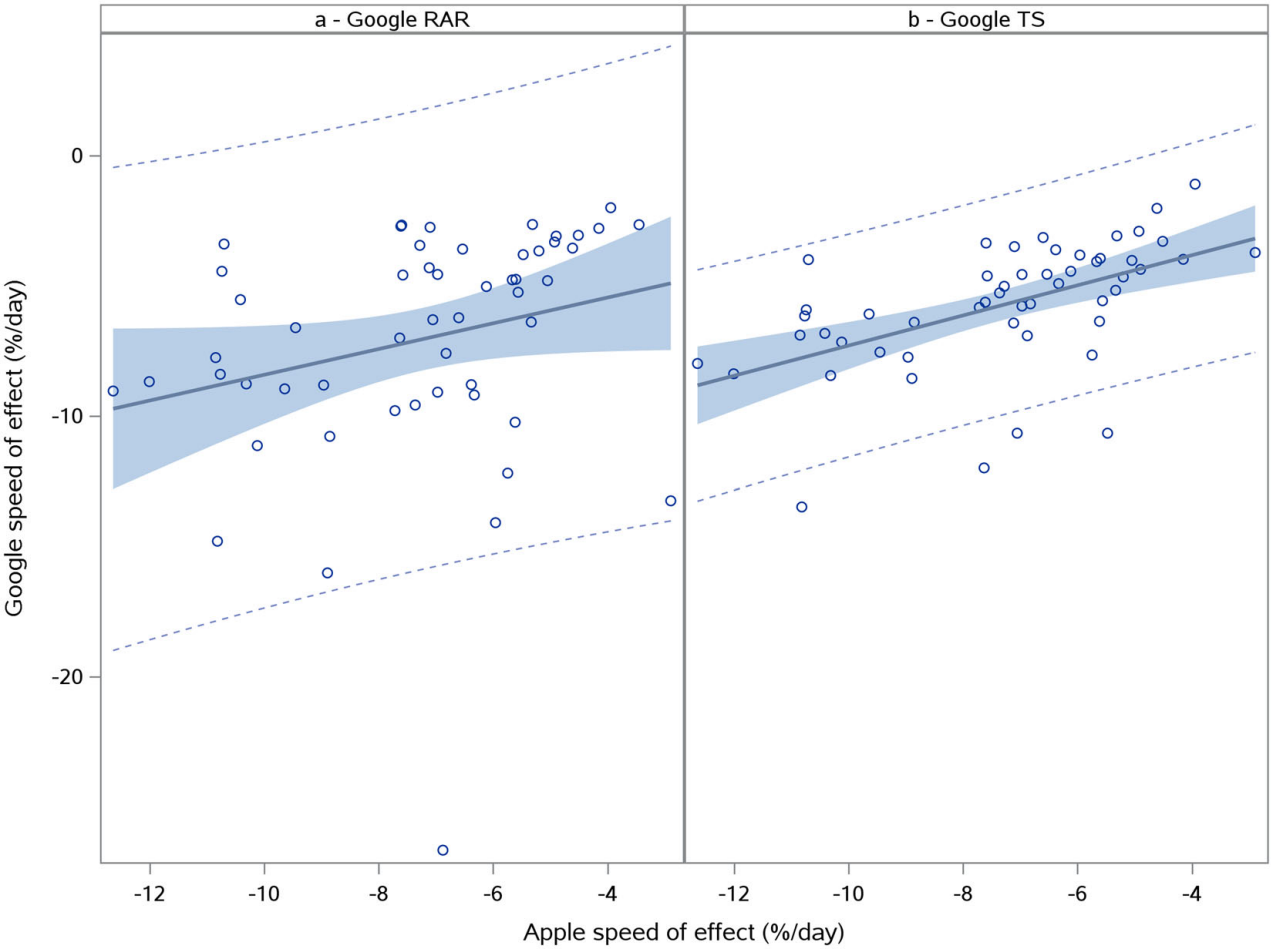
Correlation between Apple and Google mobility data for speed of effect. Plot of Apple vs Google RAR (**a**) and Google TS (**b**) speed of effect (%/day) including regression line (gray line) and corresponding 95% prediction (dotted line) and confidence limits (light blue area).

The speed of the effect of the NPIs was summarized using descriptive statistics [Table 2]. The derivation method did not result in an estimate being generated for some of the available countries. Due to noise, this parameter could not be established for between 2 to 6 countries, as reflected by the lower *n* compared to the number of countries available (*N*). This was mostly due to atypical mobility patterns like less clear lockdown effects. The average rate of reduction in mobility for the Apple data was 7.8% per day and ranged from 27.9% to 2.9% per day. The country with the fastest effect was South-Africa, where the effect of the NPIs was seen almost immediately, while the country with the slowest effect was the United Arab Emirates, where the effect of the NPIs was more gradual [Fig. 3]. The speed of effect for Apple data was normally distributed over all the countries. For Google RAR and Google TS data, normality could not be established. For this reason, we did not perform the GLM analysis on speed of effects calculated for the data from these mobility data sources. The Pearson correlation coefficient between the slopes calculated for the Apple data and those of Google RAR and TS data were 0.2566 (*p* = 0.0637) and 0.5387 (*p* < 0.0001), respectively [Fig. 4]. South Africa was, as a clear outlier, excluded from the calculation of these correlation coefficients. Both Apple and Google calculated slopes for this country were extremely steep compared to the other countries. For the other countries, although a relation could be detected for the Google TS data, this was less clear and the variation in slopes was much less for the Google data.

**Table 2:** Global summary statistics for the speed of effect of the NPIs: % mobility change/day.

Statistic	Apple (*N* = 59)	Google RAR (*N* = 124)	Google TS (*N* = 124)
*n*	57	119	118
Mean (SD)	−7.8 (3.6)	−8.7 (12.1)	−6.3 (5.2)
Median	−7.1	−5.5	−5.1
Range	−27.9 to −2.9	−96 to −1.13	−36.5 to −1.1
95% CI	(−8.7; −6.7)	(−10.9; −6.5)	(−7.2; −5.4)

*N* – Total number of countries.

*n* – Number of countries included in analysis.

SD Standard deviation.

CI Confidence interval.

### Explorative analysis of NPIs

The NPIs that were issued by the governments are categorized. An overview of the timing of these categorized NPIs in relation to the ASMD is presented in Table 3. Social distancing measures like the limitation of public gatherings were implemented in the majority of the countries (78.0%), followed by school closures (64.4%) and closure of businesses and public services (55.9%). Of these, school closure and especially closure of businesses and public services were issued closer to the ASMD indicating that might have a more direct effect than the other NPIs issued. By contrast, less burdensome NPIs in the category of public health measures were generally issued much earlier.

**Table 3:** Number and percentage of countries implementing NPIs and days/weeks before ASMD.

Category^1^	Intervention^1^	Number (%) of countries with intervention	>8 weeks before	7–8 weeks before	4–6 weeks before	2–3 weeks before	10–14 days before	7–9 days before	4–6 days before	1–3 days before
Governance and socio-economic measures.	Economic measures	32 (54.2%)	1	1	3	1	1	4	10	11
	Emergency administrative structures activated or established	22 (37.3%)	2	4	2	1	1	4	4	4
	Limit product imports/exports	4 (6.8%)^2^				1		1	1	1
	Military deployment	7 (11.9%)^2^					1		1	5
	State of emergency declared	26 (44.1%)		1		1	2	4	11	7
Lockdown	Lockdown (Partial/Full)	18 (30.5%)^3^							3	15
	Partial lockdown	17 (28.8%)						1	3	13
Movement restrictions	Additional health/documents requirements upon arrival	8 (13.6%)^2^	1		1		2		1	3
	Border checks	11 (18.6%)^2^	1	1	2		3		2	2
	Border closure	27 (45.8%)					3	6	5	13
	Domestic travel restrictions	10 (16.9%)^2^							2	8
	International flights suspension	21 (35.6%)	1				1	3	7	9
	Surveillance and monitoring	11 (18.6%)^2^	2	1	2			1	3	2
	Visa restrictions	15 (25.4%)					3	3	4	5
Public health measures	Awareness campaigns	24 (40.7%)	2	6	2	2	2	1	4	5
	General recommendations	28 (47.5%)	4	2	1	3	3	2	4	9
	Health screenings in airports and border crossings	22 (37.3%)	4	4	2		5	1	3	3
	Isolation and quarantine policies	29 (49.2%)	1	1	3	1	2	4	7	10
	Mass population testing	3 (5.1%)^2^				1		1	1	
	Psychological assistance and medical social work	3 (5.1%)^2^		1						2
	Strengthening the public health system	33 (55.9%)	3	9	1	1	3	2	7	7
	Testing policy	4 (6.8%)^2^		1	1			1		1
Social distancing	Changes in prison-related policies	8 (13.6%)^2^						1	4	3
	Closure of businesses and public services	33 (55.9%)				1		3	10	19
	Limit public gatherings	46 (78%)	1	2		5	8	10	10	10
	Schools closure	38 (64.4%)			1		3	11	11	12

NPIs occurring only in 1 or 2 countries before ASMD are not presented in the table.

^1^ Categories and interventions as defined by ACAPs.

^2^ NPIs occurring in <20% of the countries were excluded from the statistical analysis.

^3^ Lockdown NPIs were combined into a single NPI. If a country experienced both partial and full lockdowns the country was only counted once for the combined NPI.

Based on the Apple data, on average 8.3 NPIs were implemented within a country before the ASMD. Of these on average 6.9 were implemented within the last 3 weeks before the ASMD [Table 4]. Although the same NPI dataset was used, the results for the Google data differed slightly from the Apple data, due to slight differences in the estimated ASMD.

**Table 4:** Global summary statistics for the number of distinct NPIs implemented before ASMD per country.

	Statistic	Total NPIs before stabilizing	NPIs within 3 weeks before stabilizing
Apple			
(*N* = 59)	Mean	8.3	6.9
	Median	8.0	7.0
	Mode	7	7
	Range	2–16	1–12
Google RAR			
(*N* = 124)	Mean	8.5	7.3
	Median	8.5	7.0
	Mode	9	9
	Range	0–17	0–15
Google TS			
(*N* = 124)	Mean	8.7	7.6
	Median	8.5	8.0
	Mode	8	9
	Range	0–17	0–14

**Fig. 5:**
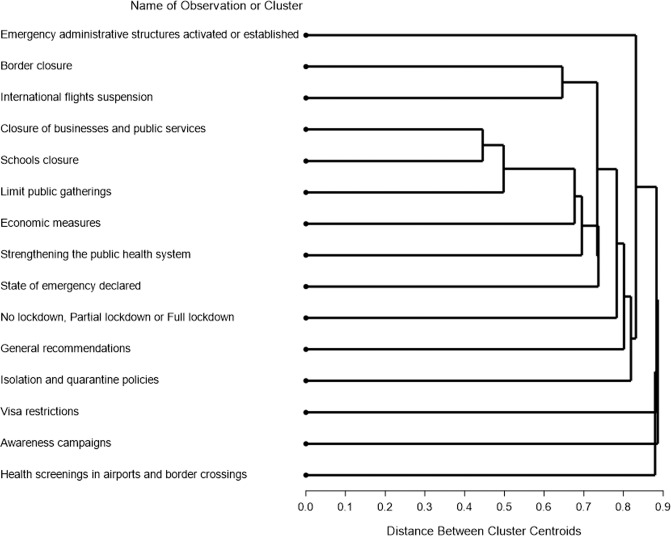
Tree diagram of correlations between individual NPIs. Tree diagram based on Cluster analysis between individual NPIs. Linked NPIs are more related (issued in same countries). The shorter the branch, the stronger the relationship.

**Fig. 6:**
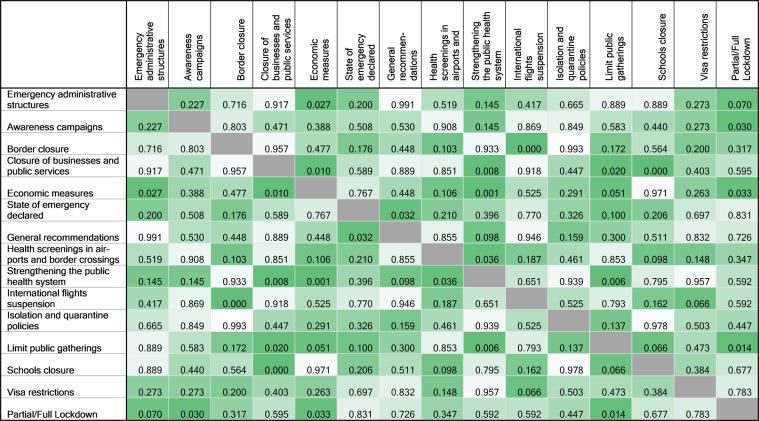
Heatmap of pairwise Chi-square associations between NPIs. Heatmap based on the *p*-values obtained from individual Chi-square tests indicating whether NPIs occurred in the same countries or not.

The overall results of the cluster analysis for assessing the associations between different NPIs are presented by the tree diagram in Fig. 5. To further investigate these correlations, Chi-square tests between pairs of NPIs were performed [Fig. 6]. As this was an explorative analysis, the resulting *p*-values were not adjusted for multiple testing. *p*-values close to 0.05 should therefore be considered with this caveat. From both analyses, close and significant relations between border closure vs international flight suspension (Chi-square *p* < 0.001) and school closure vs closure of businesses and public services (Chi-square *p* < 0.001) were observed. In addition, the limitation of public gatherings had a strong relation with closure of businesses and public services (Chi-square *p* = 0.020) as can be seen from both analyses as well. Based on the Chi-square analysis, more NPIs seem to be related to the the businesses/school closure cluster, like economic measures, strengthening the public health system and lockdown measures. These relationships and their strength given the other relationships, are depicted in the tree diagram as well.

### Effect of NPIs on mobility

The number of interventions issued before lockdown had an overall significant effect on the % mobility lost after stabilizing [Table 5] indicating that the more interventions were issued, the more the mobility is decreased.

**Table 5:** Effect of the number of NPIs on the % mobility lost after stabilizing.

Data source	Effect parameter	Estimate	*P*-value
Apple	Intercept	47.36	<0.0001
	Number of NPIs	1.90	0.0008
Google RAR	Intercept	45.65	<0.0001
	Number of NPIs	1.53	0.0057
Google TS	Intercept	44.66	<0.0001
	Number of NPIs	1.67	0.0005

The effect of NPIs on the Apple mobility data is presented in Table 6. The GLM best fit model showed significant increases in % mobility lost were attributable to lockdown measures (13.2%) and the declaration of a state of emergency (7.3%), with an additional 50.4% loss in mobility not attributable to any single NPI. Awareness campaigns and school closures were included in the model as well, with smaller, non-significant contributions. For the speed of the effect, a basic reduction in mobility of 4.9% per day was found which was not attributable to a single NPI in the model. This speed of effect was significantly increased by 1.8% per day after issuing the lockdown measures and by 1.2% per day after limiting public gatherings. School closures and the declaration of a state of emergency were included in the model as well with smaller non-significant contributions to the speed of effect.

**Table 6:** Estimates and *p*-values from the selected best fit model from the GLM analysis for the Apple data.

Endpoint	Parameter	Estimate	*P*-value
Average % mobility lost (extent of the effect)	Intercept	50.4	<0.001
Overall model *p*-value = <0.001	Awareness campaigns	5.598	0.090
	Lockdown (partial/full)	13.249	<0.001
	School closures	4.766	0.084
	Strengthening the public health system	−3.917	0.167
	State of emergency declared	7.261	0.008
Slope (speed of the effect in %/day)			
Overall model *p*-value = <0.001	Intercept	−4.876	<0.001
	Limit public gatherings	−1.222	0.075
	Lockdown (partial/full)	−1.787	0.006
	School closures	−1.028	0.080
	State of emergency declared	−0.93	0.101

The estimates and *p*-values from the selected best fit model for the Google RAR and Google TS data are presented in Table 7. As was seen with the Apple data, the presence of either a partial or full lockdown in the countries contributed to the largest increase in the average % mobility lost for Google RAR (16.6%) as well as for Google TS data (16.4%). The declaration of a state of emergency within the countries had a significant increase of 4.9% in the average % mobility lost for the Google TS data, where the closure of businesses and public services had a significant increase of 5.8% in the average % mobility lost for the Google RAR data.

**Table 7:** Estimates and *p*-values from the selected GLM best fit model for the Google data.

Endpoint	Parameter	Estimate	*P*-value
Google RAR			
Average % mobility lost (extent of the effect)	Intercept	45.699	<0.001
Overall model *p*-value = <0.001	Lockdown (partial/full)	16.622	<0.001
	Closure of businesses and public services	5.791	0.040
	State of emergency declared	5.040	0.071
Google TS			
Average % mobility lost (extend of the effect)			
Overall model *p*-value = <0.001	Intercept	50.344	<0.001
	General recommendations	−4.000	0.1156
	Lockdown (partial/full)	16.371	<0.001
	State of emergency declared	4.868	0.046

## Discussion

In this study, we utilized Google and Apple mobility data^12,13^ to assess (1) the magnitude of the change in mobility and (2) the rate of transition between the pre- and post-lockdown states during the COVID-19 pandemic. We hypothesise that the magnitude of mobility loss describes the capacity for a given country’s population to self-isolate and the rate of the mobility loss reflects the degree of urgency at which NPIs are enacted. Although the Apple data and Google (RAR and TS) data are derived from different mobility sources, the magnitude of change, represented by the mean ***average % mobility lost after stabilizing*** for these different data sources, were comparable. An average decrease in mobility of 60.6% was observed for the Apple mobility data while those of the Google RAR data and the Google TS data were 56.9% and 57.2% respectively. The high and significant correlation between the independently estimated parameters of Apple and Google (RAR: *r* = 0.7824, *p* < 0.0001; TS: 0.7607, *p* < 0.0001) indicate that, in general, both show similar effects on mobility. While Apple data (generated when requesting directions in Apple maps) includes requests from Apple computers, most of the requests are likely issued from mobile phones as the penetration of these devices is higher and phones are typically the devices used for navigation. The global penetration of mobile subscriptions is high with 104 subscriptions /100 inhabitants in 2018^16^. However, for a number of countries, especially in African countries, like Eritrea with 20/100 in 2019, it is considerably lower. The Apple dataset only included countries that had a high penetration of mobile subscriptions with the lowest for India (84 subscriptions/100 inhabitants) and highest for Hong Kong (209/100)^16^. Although Apple market share worldwide is only 13.5% in Q2 of 2020^17^ this varies greatly between countries. Apple states that the inclusion of countries in the dataset is based on the number of requests issued within the country^12^. With the knowledge that Apple and Google datasets are derived from a large proportion of the national populations, and using different modes of measuring mobility, the high correlation between the Apple and Google data indicates they are indeed representative of phone user mobility trends in general. While there may be some intrinsic biases, these are likely small and at the very least the trends can be said to apply to the substantial fraction of the population who contributed mobility data.

While the Apple data was based on direction requests, Google data is based on actual visits to specific places. As the Google data did not depend on the type of smartphone used (iOS or Android), the coverage of this data is more than twice that of the Apple data (124 vs 59 countries). Google uses data from people with a google account who have their location history switched on to assess crowding of specific places. Due to the large number of contributing users we consider this data to be as good an approximation of general mobility in a country as is presently possible. Data for Retail and Recreation (RAR) and Transits and Stations (TS) showed clear lockdown effects like the Apple data did. However, as RAR and TS are more specific for a location than the Apple data, it might depend more on the country’s infrastructure, traditions and habits whether effects are visible. To improve consistency and reduce variability, combining the Google data for different modalities could provide an even more consistent pattern than was established in this study.

The number of unique NPIs implemented within the 3 weeks before stable low mobility had a significant effect on the average % mobility lost after stabilizing. This was also observed by Islam et al.^18^, who saw an additive effect on incidence rates of COVID-19 of five of the main NPIs issued. Our finding of close correlation between NPIs may, in part, explain why, in their study, the sequence of NPIs did not show a consistent association pattern with COVID-19 incidence. We found that effects of individual NPIs on the decrease in mobility were hard to distinguish as they were often issued within a few days from each other. Two of the most closely associated NPIs were school closures and closure of businesses and public services. The latter effect was selected in the Google RAR model while the first was selected in the more general Apple direction model. This as well indicates that although comparable, the Google data is slightly more specific to certain activities and national habits.

The Apple data showed a 50.4% mobility loss that was not attributable to a single NPI in the best fit model. The Google TS model was similar in this regard (50.3%), while the Google RAR model was more specific with 45.7% unattributable loss in mobility. This relatively large unattributable part is likely to be caused by the high association of individual NPIs with each other. Despite that, all models showed an additional significant reduction in mobility attributable to lockdown measures (Apple: −13.2%, Google RAR: −16.6% and Google TS: −16.4%). Further NPIs that contributed to the loss in mobility were “state of emergency declared” (Apple: −7.2% [*p* = 0.008], Google RAR: −5.0% [*p* = 0.071] and Google TS: −4.7% [*p* = 0.046]) and “school closure” (Apple: −4.8% [*p* = 0.084]) or “closure of businesses and public services” (Google RAR: −5.8% [*p* = 0.040]). Although not significant, awareness campaigns seem to have an additional effect on mobility reduction (Apple: −6.0% [*p* = 0.090]). In the toolkit of NPIs, school closures and lockdowns (state of emergency) while potent, are also highly costly to the economy, while awareness campaigns are potentially effective but less costly. These findings also noted by Haug et al.^11^ and could provide a lower cost NPI option, which could in the future be optimised based on the mobility data and put to greater use.

For the speed of the effect observed between the start of decline and the ASMD, analysis of the Apple data showed a reduction in mobility of 4.9% per day which was not attributable to a specific NPI. Although the implementation of a lockdown is expected to differ greatly between countries (partial to full lockdown), an average effect on increase in speed of mobility reduction of 1.8% per day was still observed. Contrary to the magnitude of the mobility loss model, limiting of public gatherings was selected as having an effect in the speed of effect model (decrease 1.2%/day, *p* = 0.075), suggesting that these limitations had a speeding up effect on the NPIs issued in a country.

Most studies to date assessed the impact of NPIs on COVID-19 case numbers, deaths and the basic reproduction number (*R*_t_)^7,10^. The generalized NPIs, as obtained from the ACAPS database, are catalogued with best effort and may not be consistent in how strictly they are implemented and adhered to in different countries. They are often issued jointly and different NPIs are, as we saw in this study, moderately correlated. This was also substantiated by Hale et al.^19^ who proposed an aggregate stringency index based on all the NPIs issued in a country. As this index is a combination of different NPIs and their characteristics, it is according to Hale et al. a better predictor of death rates than the individual NPIs. The variability of NPI implementation and adherence are not provided in the NPI database. This was illustrated by Drake et al.^8^ who examined the effect of NPIs on mobility in the UK using the same Google data. They observed a decrease in adherence to movement restrictions over time though this overlooks the between-country-effects of NPIs. The % remaining mobility in a country, combined with the duration of low mobility, is therefore likely to be a better estimate to correlate with the case and death rates than the specific NPIs issued. This together with other factors influencing the spread of COVID-19, like infectiousness of the virus and distancing measures, we argue should yield an improved model for prediction of cases and deaths in a specific country or sub national regions.

Cases, deaths and *R*_t_ are difficult to measure, especially in a timely manner; additionally they encounter difficulties accounting for asymptomatic cases without early and large-scale population testing. Mobility data, on the other hand, could be a useful metric as it is widely available, implemented continuously, passively and increasingly uniformly in most countries (with the caveat of mobile network penetration and device ownership excepted). When assessing the direct effect of NPIs on COVID-19 cases, there will be a lag of at least 14 days due to incubation timing and a delay of disease transfers. Rather than the delayed COVID-19 direct measures like cases and deaths, the effects of loosening the NPIs and the behavioural rebound of the population can be more immediately examined using the mobility rates. Based on this, more direct and accurate changes in issuing or withdrawal of NPIs can be made resulting in better health care planning and governmental policies.

A key issue noted in the early pandemic was the uncertainty and requirement to “build up” monitoring programmes. While the Google and Apple data we used has utility in the present, there is ample opportunity for future expansion of this approach by using Google and Apple location data to derive more specific measures around e.g. local population mixing (time-dependent geo co-localisation at street or postcode level). We argue that this could be done in a similar, privacy preserving manner, using the differential privacy approach used in TS and RAR and generating anonymised aggregate metrics. Even more accurate behavioural changes in activity after the implementation of NPIs can be observed in greater detail by analysing data collected directly through smartphones and wearables^20^. However, as this actively requires the permission and cooperation of the user population, as well as the analysis of huge amounts of data, this is more feasible for sub-populations in selected countries.

Pre-pandemic data for both Apple and Google data, as is typical of mobility data, showed strong weekday periodicity which varied considerably and also characteristically from country to country. The weekday periodics for Apple data were generally more pronounced before stabilization and disappeared thereafter as typical working routines broke down during lockdown. On the contrary, as the Google data had corrected for weekday effects obtained from baseline, when the real weekday effect diminished during lockdown, clear overcorrecting was visible for the Google data, showing reverse weekday effects in the mobility graphs. For this study, we strived to correct the (reverse) weekday effects after lockdown based on the average post-lockdown weekday deviations to get a more accurate % mobility left after stabilization. In addition, any remaining weekday effects were removed by using the average of the 2 weeks after the stable mobility date. Visual inspection of the results showed an accurate overlay of the resulting % mobility left the actual data. For the speed of effect we accounted for smaller deviations in mobility to overcome the weekday effect. However, in case of rapid decreases, i.e. within a few days, the weekday effect could have resulted in a bias in the estimates of this parameter.

In a number of countries, the % mobility was less stable, with increased activity starting again after a few days of stabilization. Although the resulting estimated % remaining mobility is, in these cases, higher than the lowest mobility reported, it still gives a reliable representation of the mobility in the first two weeks of lockdown. By comparison using the moving weekday average, with our method, a more accurate adjusted stable mobility date (ASMD) and start of decline date could be calculated. Due to inconsistencies in the weekday effects from week to week, there is still a quantity of variation remaining in the data after correcting for this based on our method. Therefore, it was not possible to assess the start of decline and start of stability using standardized methods like deriving inflexion points from a LOESS fit of the data.

**Fig. 7:**
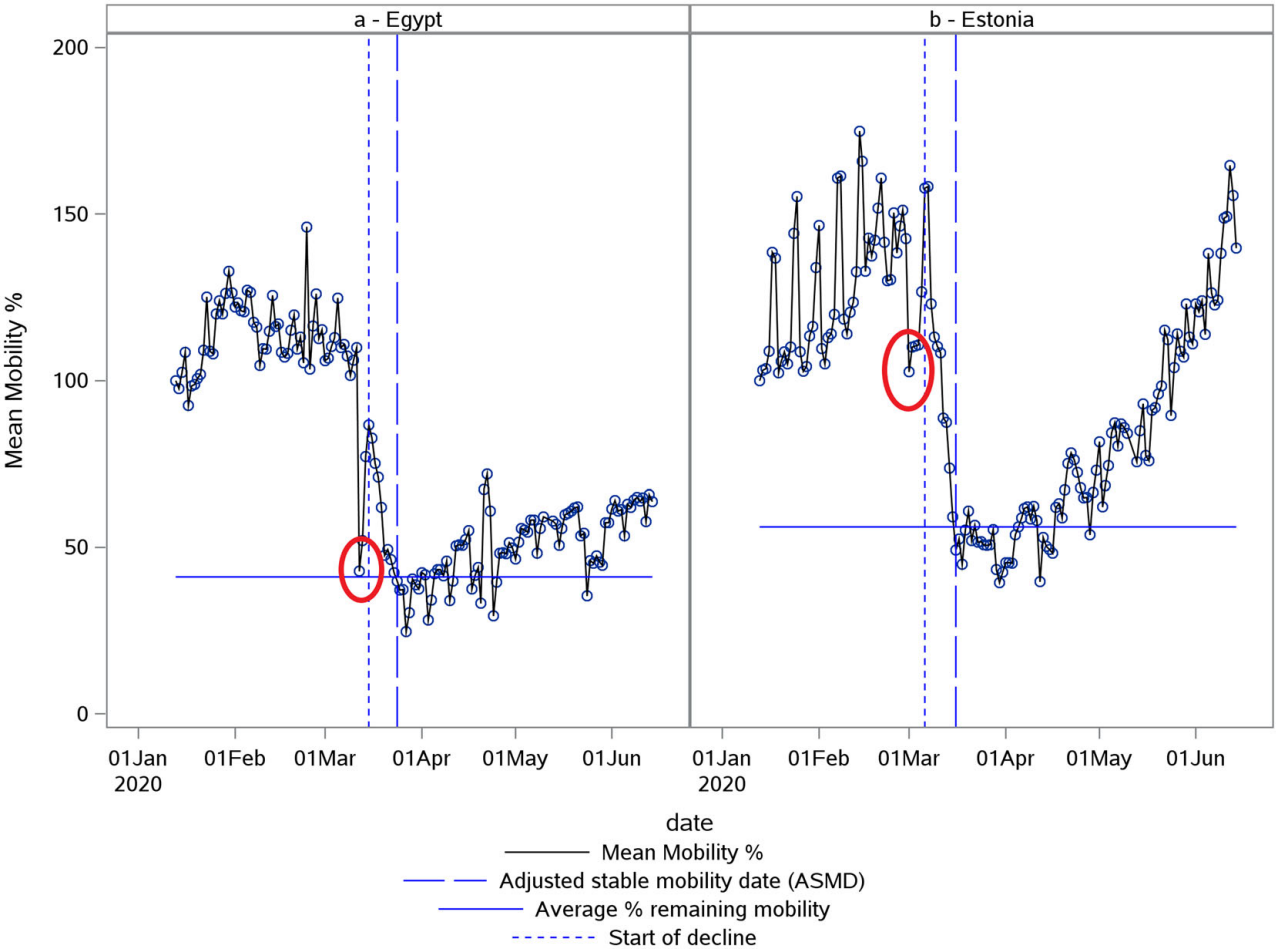
Examples of effects of significant events on mobility. Plot of Apple normalized mobility data in % deviation from baseline indicating the Egypt flood just before lockdown (**a**) and the elections in Estonia (**b**). The first vertical (blue small) dotted line indicates the estimated start of decline. The second vertical (blue large) dotted line indicates the adjusted stable mobility date (ASMD). The horizontal line indicates the estimate for average % remaining mobility. Red circles mark atypical events, flooding in (**a**) and elections in (**b**).

In addition to the weekday effects in some cases, significant events, other than NPIs, had an effect on mobility, like the flooding in Egypt of 11 March 2020^21^, Independence Day of 24 February 2020 in Estonia and the elections of 29 February 2020 in Slovakia [Fig. 7]. Upon visual inspection of the corresponding country plots and the resulting parameters, these events did not seem to have a significant effect on the % remaining mobility or the rate of the decline of mobility although in some cases it had an effect on the start of the decline date which was not used in the analyses.

Based on visual inspection, the rate of decline, as calculated between the start of decline and the ASMD, was approximately linear and overlaid per country mobility data. Only for South Africa did the decline seem to occur in two phases, starting with a slow degradation and eventually a very fast decline within a few days which is illustrated by the outlying value for the speed of effect for South Africa. This two-phase decline can be explained by the initial lower effective NPIs issued and subsequent full-lockdown after which mobility was rapidly reduced to a very low level. This illustrates that the model of speed of decline is more viable in the initial clear mobility transition and is less usable for the later periods in the COVID-19 epidemic where NPIs are more gradually issued and withdrawn. By including additional inferences for changes in the speed of effect and using a variable rate, this issue can be accounted for in future analyses.

## Methods

### Data sources and availability

Google and Apple mobility datasets were downloaded from the sites of their respective providers^12,13^. The Apple mobility data shows a relative volume of direction requests for driving, walking and train transits per country compared to a baseline volume on 13 January 2020. Apple data was extracted on 14 June 2020 and was used in the analysis for the time period from 13 January 2020 to 14 June 2020 which includes the first period of clear mobility drop in all countries. All datasets used for the analyses are prepared with the intent of making them available for the public. The data available to the public are not individually identifiable and therefore analysis would not involve human subjects. Analysis of de-identified, publicly available data does not constitute human subjects research as defined at 45 CFR 46 and that it does not require IRB review^22^. The specific downloaded data sources as well as the derived aggregated data and parameters are available via https://github.com/MariskaBurger/Covid-19-analysis-results/tree/master.

The Google mobility data shows movement trends by country across different categories of places based on the mobility history tracker which is voluntarily turned on by the users^13^. This data shows the change in quantity of visitors to these categorized places compared to baseline. Baseline for the Google data was defined as the median value from the corresponding weekday in the 5-week period from 3 January 2020 to 6 February 2020. Google data was extracted on 7 June 2020 and was used for the time period of 15 February 2020 to 7 June 2020. As only the RAR and TS categories showed clear lockdown effects, they were used in the analyses.

The NPIs recorded on a global level were extracted from ACAPS^2^. Every action taken by governments in response to COVID-19 is captured in this database. NPIs could be re-issued or prolonged, which is recorded in the ACAPs dataset as well. NPI data extracted on 14 June 2020 was used in the analysis and included interventions starting from 01 January 2020 until the extraction date.

### Analysis methods and derivations

All analyses were performed using SAS^®^ Studio 3.7 Enterprise Edition. We calculated average mobility per country per day in the Apple data using the driving, walking and train transit data. In order to make the data comparable with the Google mobility data, the percentage change values were added or subtracted from 100%, which is similar to the pre-lockdown mobility.

**Fig. 8:**
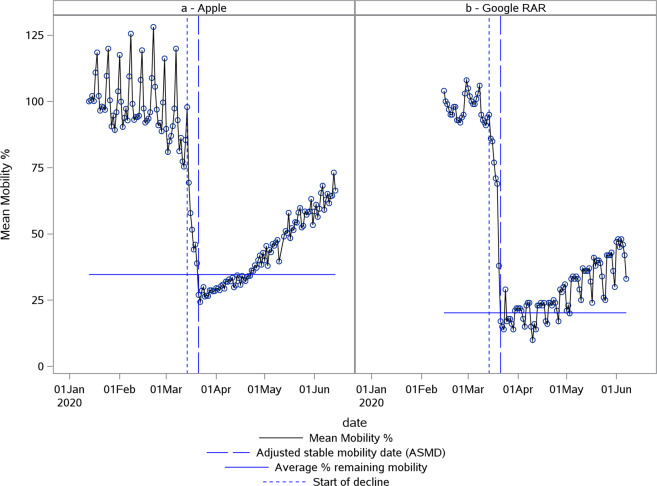
Comparing weekday effects in Google and Apple mobility data. Plot of Apple (**a**) and Google RAR (**b**) normalized mobility data in % deviation from baseline for Colombia. The first vertical (blue small) dotted line indicates the estimated start of decline. The second vertical (blue large) dotted line indicates the adjusted stable mobility date (ASMD). The horizontal line indicates the estimate for average % remaining mobility.

Weekday effects pre- and post-lockdown were not consistent as can be seen from the Google RAR and Apple mobility data of Colombia [Fig. 8]. While Apple did not correct for weekday effects by taking a baseline of one reference day, Google did take a baseline per weekday resulting in overcompensated post-lockdown data. To get a reliable estimate of percentage remaining mobility, time series data during lockdown were smoothed to account for the weekday effect by adding the average weekday deviation to the % mobility. This was only performed for the post-lockdown period by starting from the first Sunday where the mobility decreased below 70%. The ***weekday deviation*** was calculated as the difference between the actual mobility on the specific weekday and the average mobility for that corresponding week.

To assess the effect of NPIs on the magnitude and rate of the reduction of mobility, the following two primary parameters were derived after the smoothing of the weekday effects:

1. The ***average % mobility lost after stabilizing*** to assess the magnitude of the effect of the NPIs.

2. The rate of transition (gradient) between the start of the decline and the stabilizing point to assess the ***speed of the effect*** of the NPIs.

The initial cut-off value for the start of stable mobility was defined as the midpoint between 100% and the average percentage mobility of the 5 lowest reported mobility rates. The initial ***stable mobility date*** was then calculated as the first date where the mobility dropped below this initial cut-off value and did not increase more than 10% (absolute day-to-day change) for at least 7 consecutive days. To establish mobility after the transition, the ***average % remaining mobility after stabilizing*** was calculated as the average of the mobility for the 2 weeks following the stable mobility date and then subtracted from 100% to produce the ***average % mobility lost after stabilizing***. To get a more precise estimate of the start of stable mobility, an ***adjusted stable mobility date (ASMD)*** was determined for each country as the first date where the mobility dropped below the average % remaining mobility after stabilizing. Visual inspection of the resulting parameters showed an accurate estimation for nearly all countries included in both Apple and Google data (see supplementary Figures 1 “Apple mean mobility per country”, Supplementary Fig. 2 “Google RAR mean mobility per country” and Supplementary Fig. 3 “Google TS mean mobility per country” https://github.com/MariskaBurger/Covid-19-analysis-results/tree/master).

In order to calculate the speed of the effect of the NPIs, the ***start of the decline*** of the mobility was calculated as the last occurrence before the adjusted stable mobility date (1) with changes in mobility rate of less than +7% (so slight increases were also considered); (2) without 3 or more consecutive changes between −2% and 7%; and (3) with a total decline from the beginning of the decline to the last declining observation of more than 60% of the average % mobility lost after stabilizing. This was done to exclude periods where the % mobility stayed relatively constant for a period of time before declining. These cut-off values accounted for potential weekday effects and were determined by iteration through different values and visual observation of the resulting estimates. After finding the start of the decline date for each country, the ***gradient*** of the line connecting the start of the decline and the ASMD date was calculated as the ***speed of effect***.

The correlation between the different Google and Apple mobility data sources (Apple, Google RAR and Google TS) was examined by creating correlation graphs and calculation of Pearson correlation coefficients for the derived parameters *average % lost after stabilizing* and *speed of effect* for those countries where both sources were available.

For each country, the total ***number of unique NPIs*** implemented before the ASMD and the number of unique NPIs implemented within 3 weeks before ASMD were calculated. These numbers were slightly different between the two mobility data sources due to slight differences in the ASMD estimated for the different data sources. In case of re-issuing of an NPI, only the first implementation date for each NPI was used as reissued NPIs were generally extensions of the NPIs already issued and the first date of the NPI represented the earliest time point the mobility could be affected. For the statistical analyses, each country and NPI was dichotomized to whether NPI was implemented within 3 weeks before the ASMD or not.

In order to determine the correlation in occurrence of different NPIs, we performed cluster analysis based on the centroid method. This method measures the (squared) euclidean distance between the centroids or means of the clusters and results in unique clusters of different NPIs. For this analysis, Jaccard coefficients between each pair of NPIs were calculated as the number of countries that are coded as 1 for both NPIs (in each pair) divided by the number of countries that are coded as 1 for either or both NPIs (in each pair). To verify the results of the cluster analysis, the association between the different NPIs was further analysed using a Chi-square test for all possible combinations of NPIs. In accordance with the analyses of NPIs on the parameters derived from Apple and Google data, both Cluster and Chi-square analyses were performed on the NPIs which were issued within the last 3 weeks before ASMD and occurred in more than 20% of the countries. Results for these analyses based on the ASMD of the Google data sources were slightly different from the Apple data due to small shifts in the ASMDs. They however are equivalent and corresponding Google results are therefore not presented in this article.

The effect of the NPIs on average % mobility lost after stabilizing and speed of the effect were analysed using generalized linear models (GLM). We used the lowest Akaike’s Information Criterion (AIC) value to determine the best fit model. All assumptions related to generalized linear models were verified to hold true **(residual errors are independent, normally distributed and have constant variance)**. NPIs that occurred more than 3 weeks before the adjusted stable mobility date were less likely to have an effect on lockdown and seemed to have an obscuring effect on the analysis results. By classifying the time before stabilization (as presented in Table 3) in the analysis and testing the resulting models, the 3-week cut-off appeared to give the best univocal results. Therefore we have disregarded NPIs issued before from further analysis. In addition, for the same reason of convergence of models and reliability of model effects, we have excluded NPIs that occurred in less than 20% of the countries.

### Reporting summary

Further information on research design is available in the Nature Research Reporting Summary linked to this article.

## Supplementary information

Supplementary Information

Reporting Summary

## Data Availability

For the analyses described in this article we have used freely available data sources from Apple, Google and ACAPs. The corresponding used data can be downloaded via their respective websites^2,12,13^. The downloads used for the described analyses and datasets with derived parameters are available via https://github.com/MariskaBurger/Covid-19-analysis-results/tree/master.

## Code availability

Code for data cleaning and analysis is provided as part of the replication package. It is available at https://github.com/MariskaBurger/Covid-19-analysis-results/tree/master for review.
