# Supplementary material for: Measuring the effect of Non-Pharmaceutical Interventions (NPIs) on mobility during the COVID-19 pandemic using global mobility data
Source: NPJ Digit Med. 2021 May 13;4:81. doi: 10.1038/s41746-021-00451-2 (PMC8119480; doi:10.1038/s41746-021-00451-2)

Supplementary Figures 1 Apple mobility profiles per country

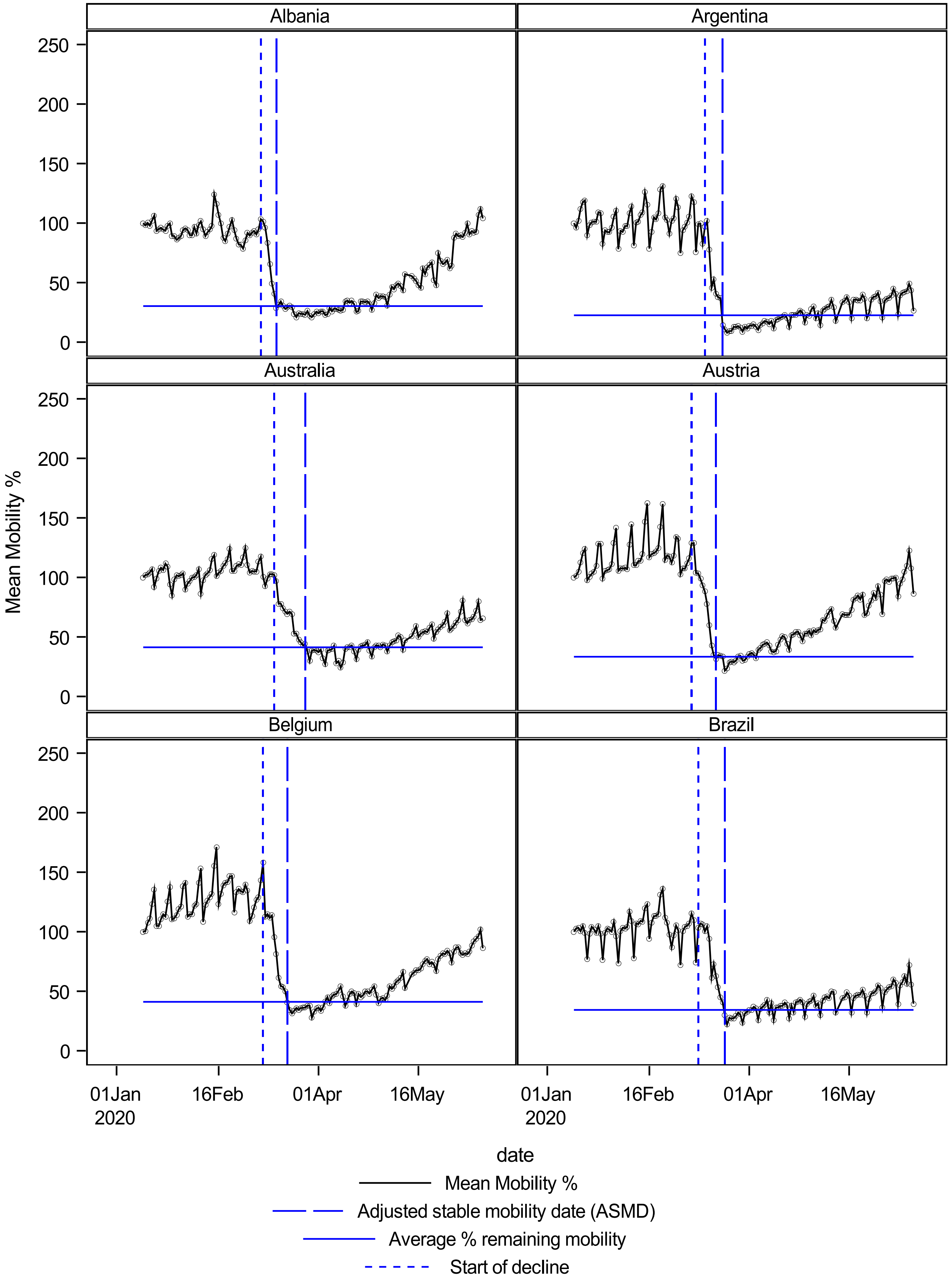

Supplementary Figures 1 Apple mobility profiles per country

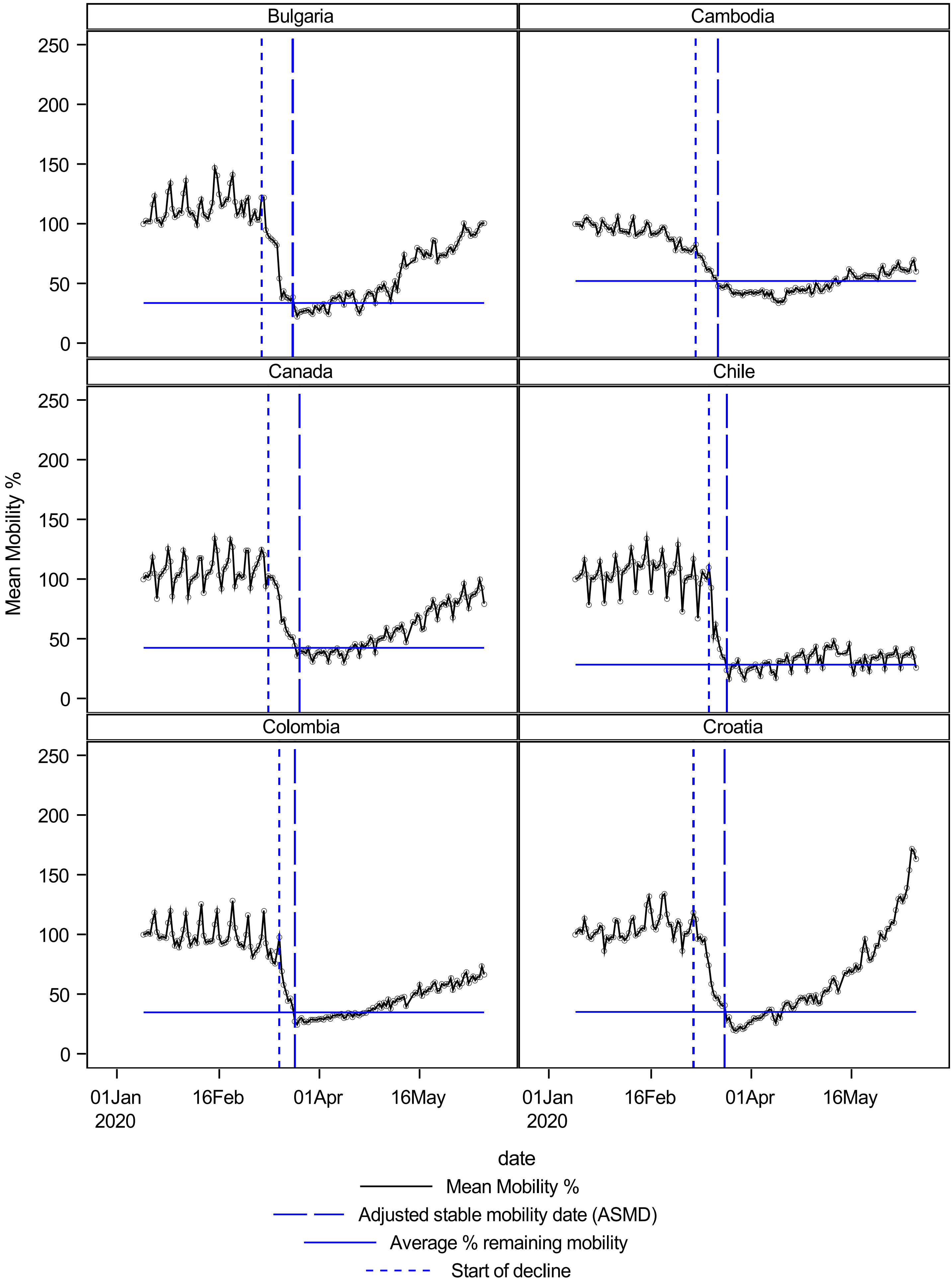

Supplementary Figures 1 Apple mobility profiles per country

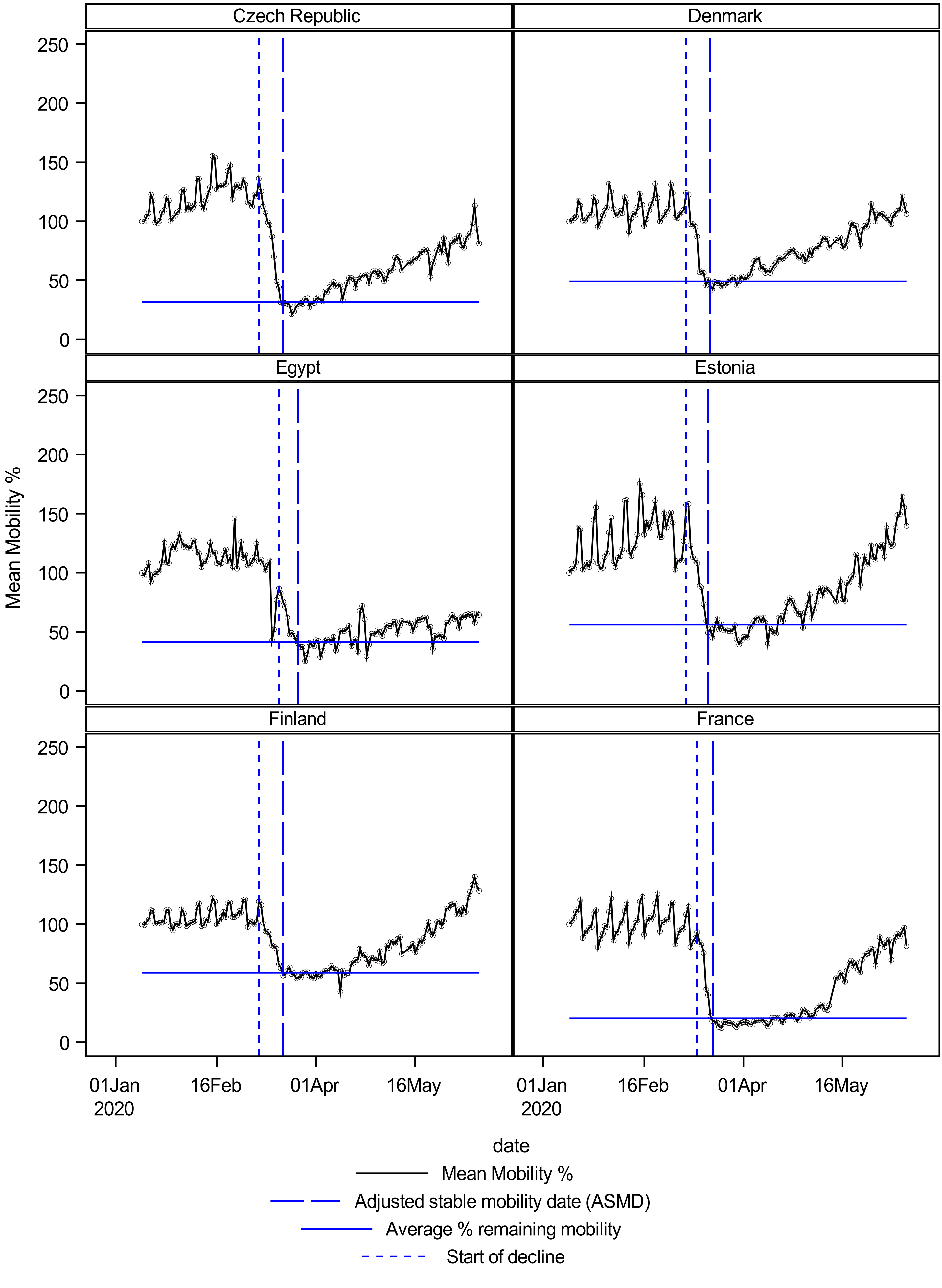

Supplementary Figures 1 Apple mobility profiles per country

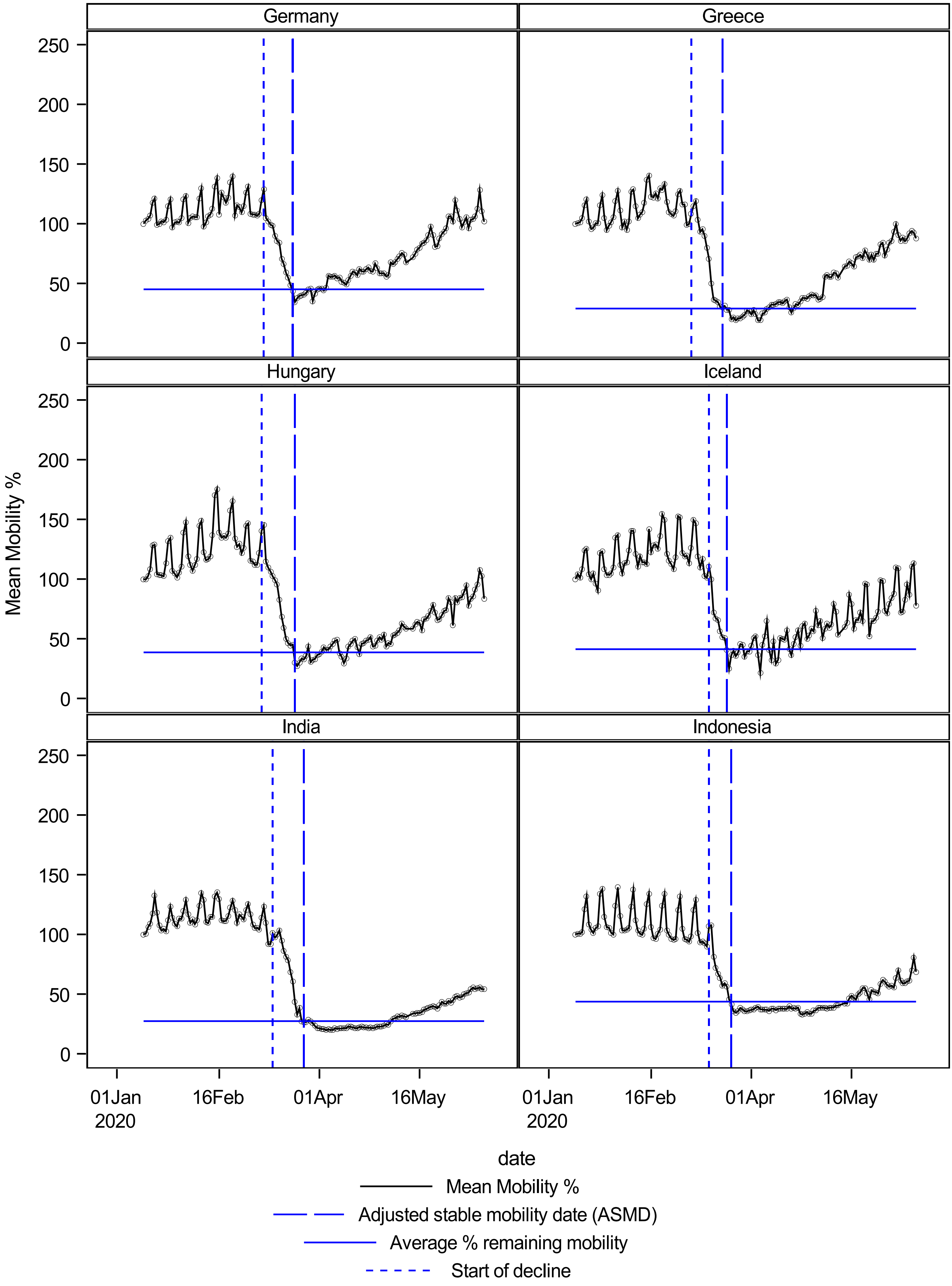

Supplementary Figures 1 Apple mobility profiles per country

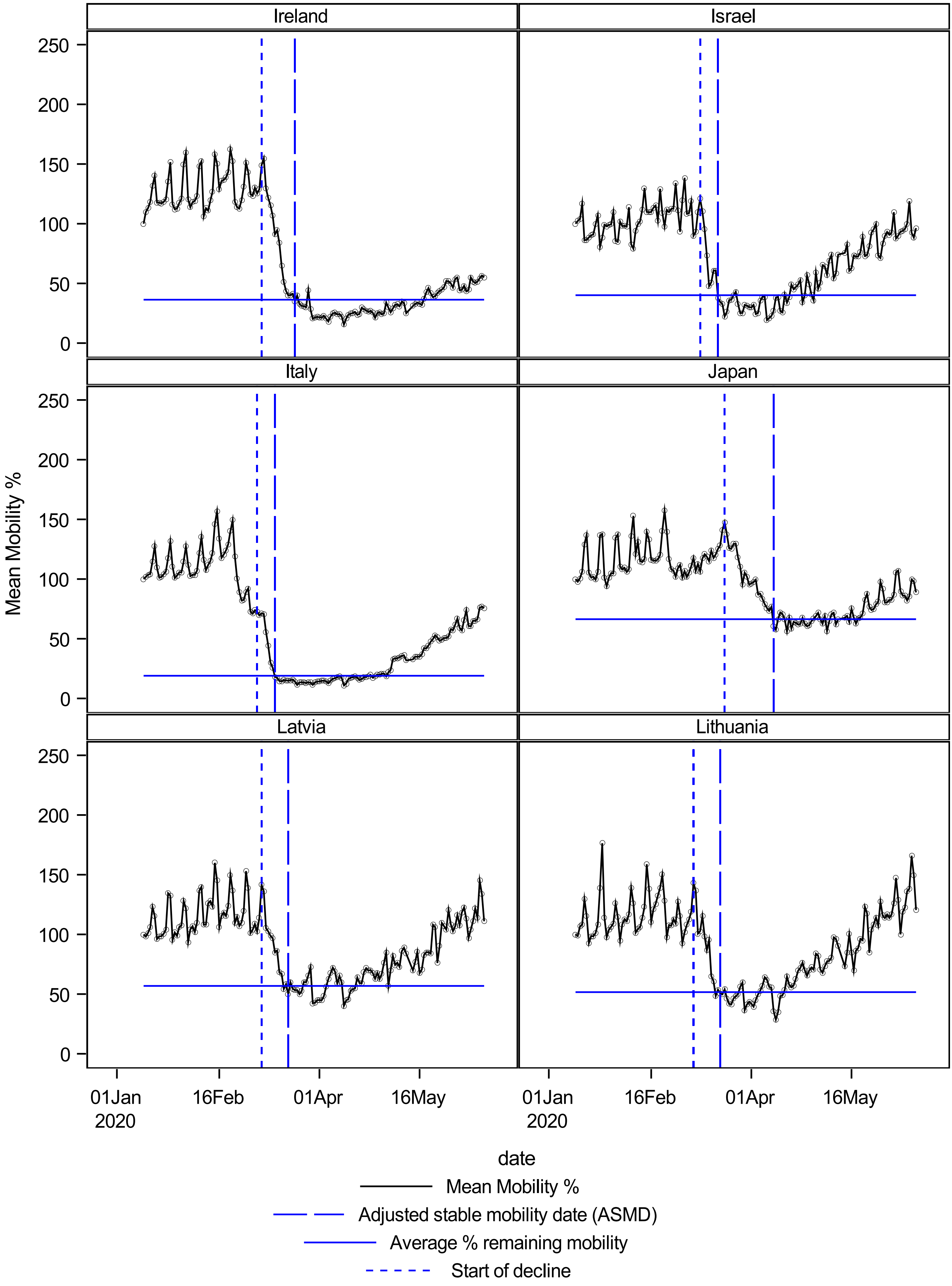

Supplementary Figures 1 Apple mobility profiles per country

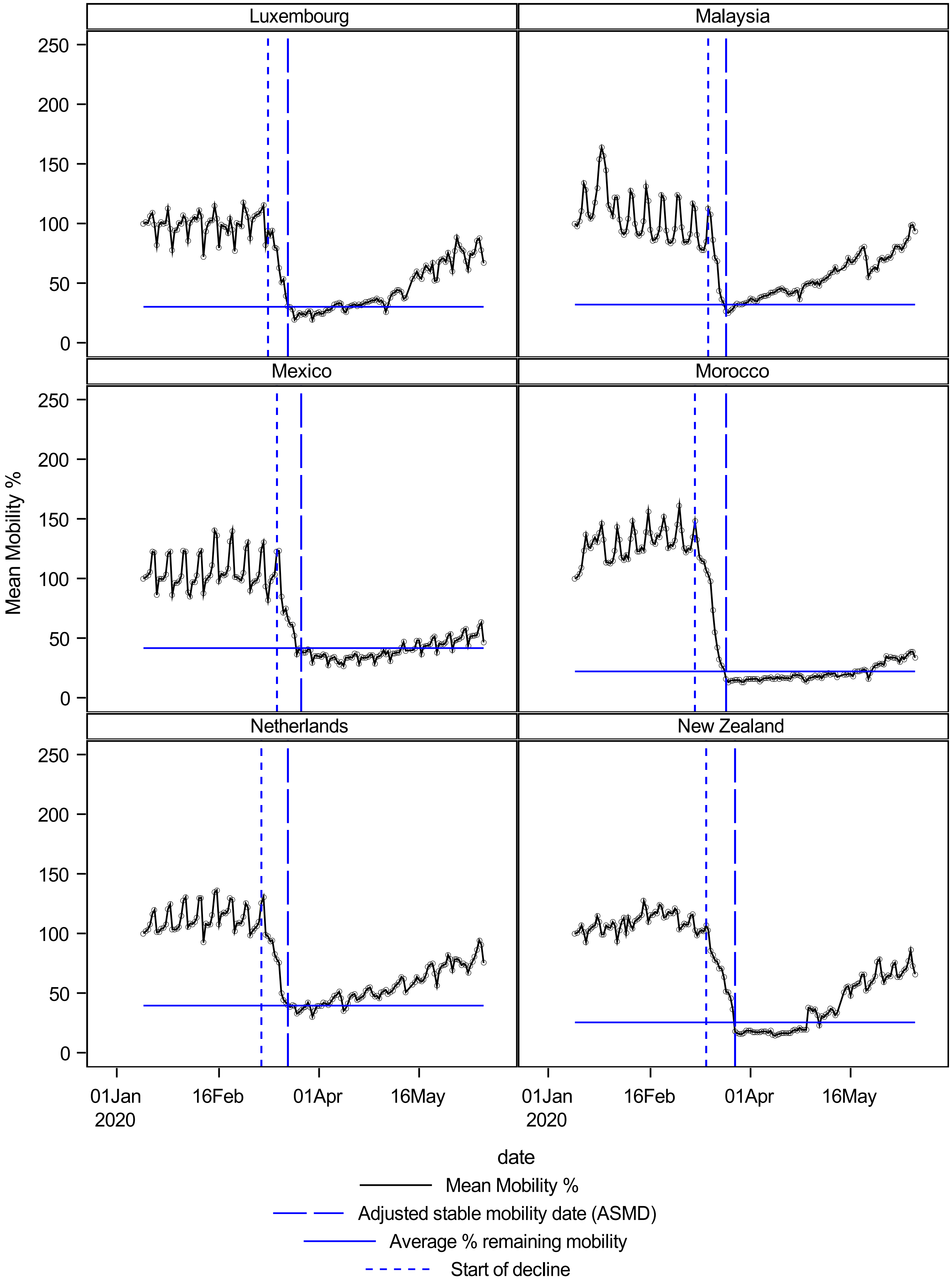

Supplementary Figures 1 Apple mobility profiles per country

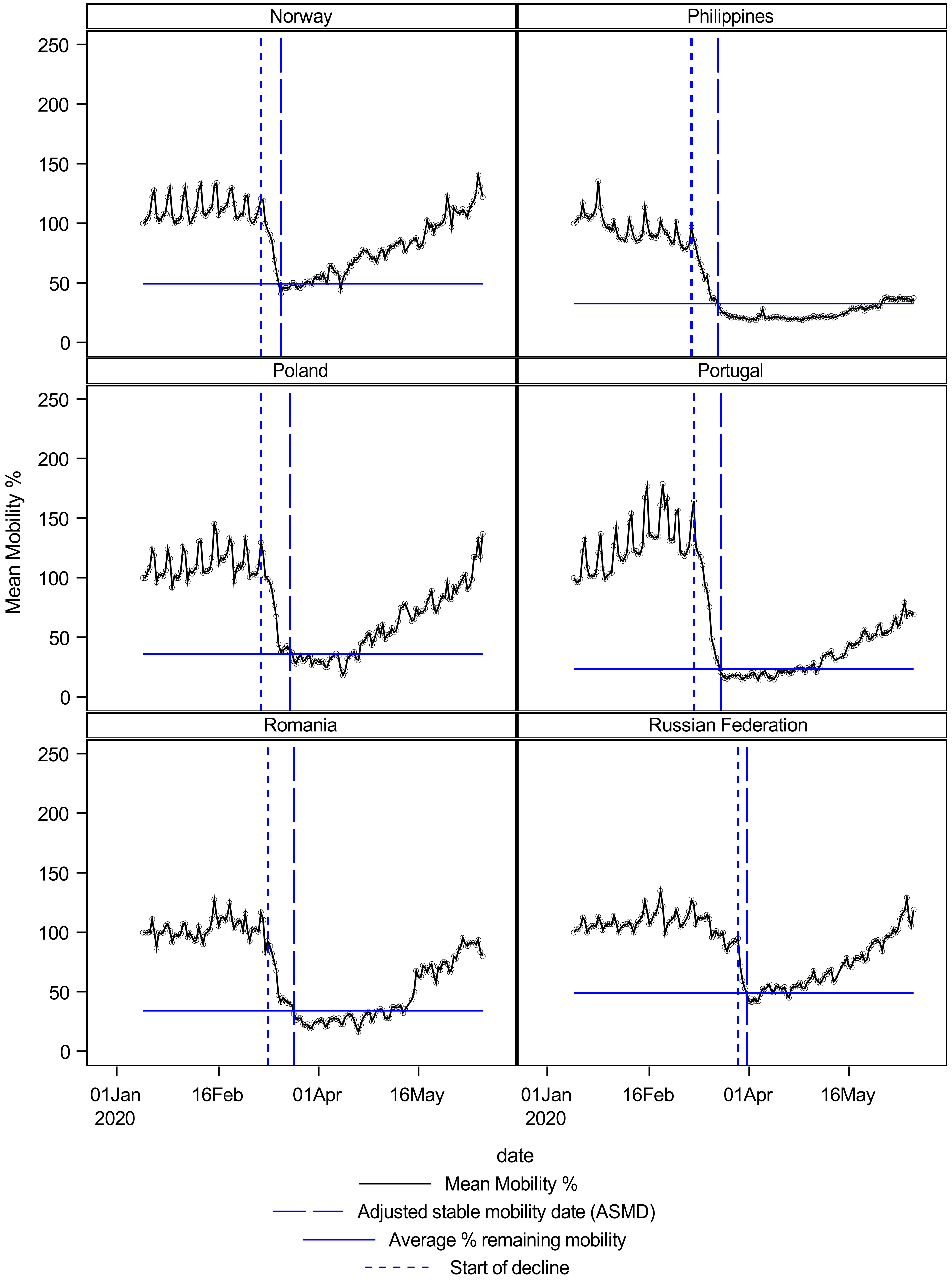

Supplementary Figures 1 Apple mobility profiles per country

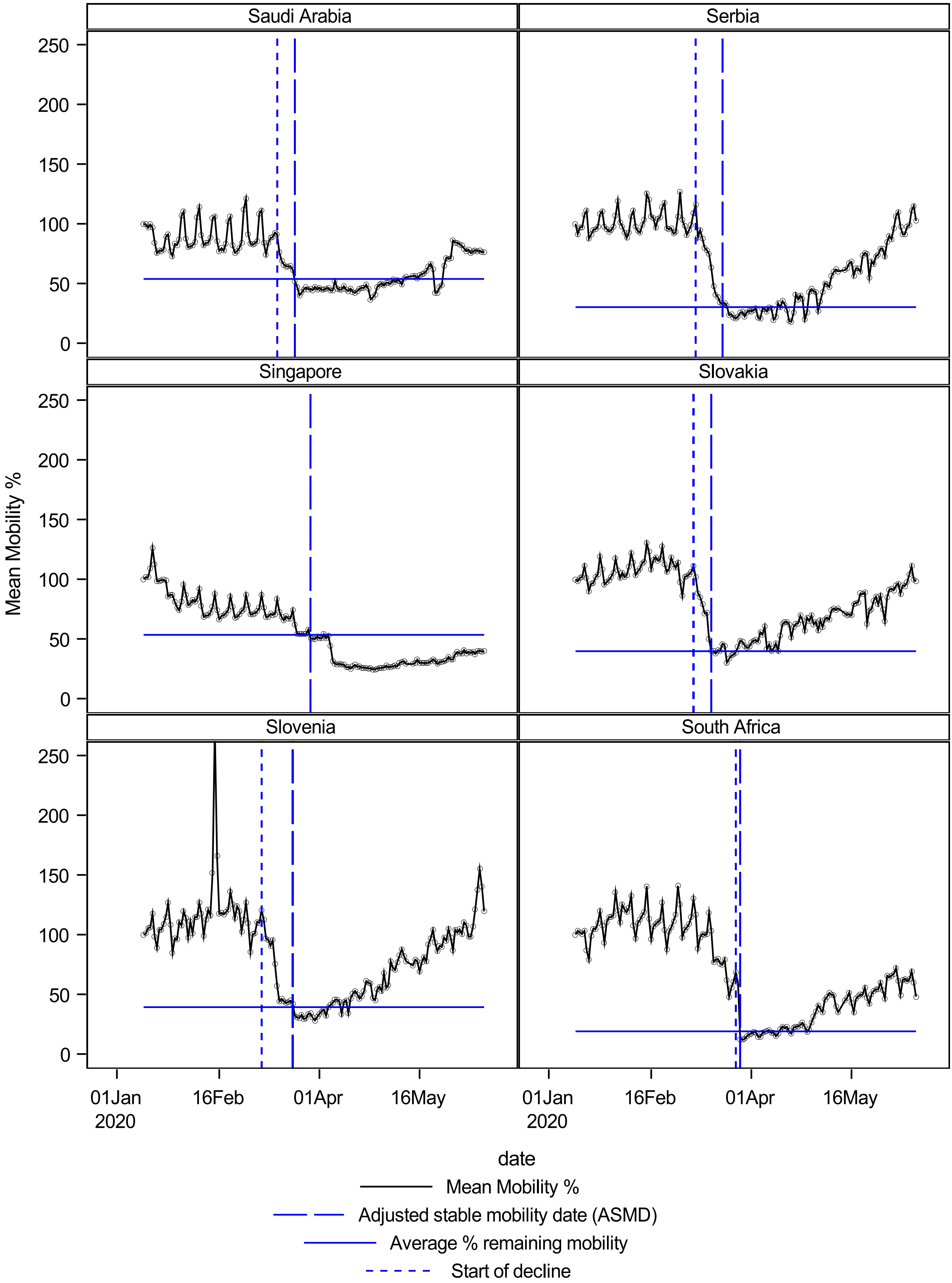

Supplementary Figures 1 Apple mobility profiles per country

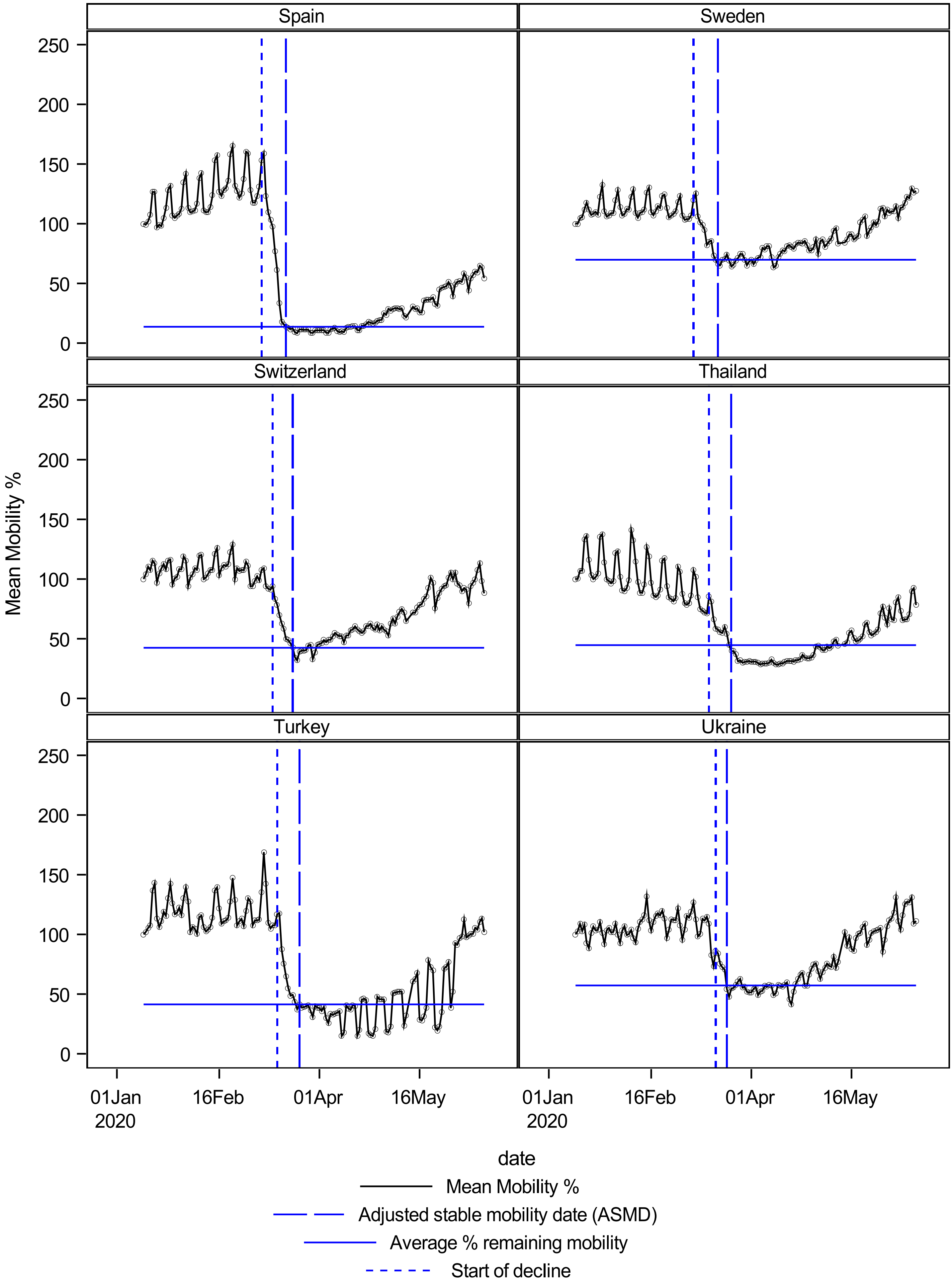

Supplementary Figures 1 Apple mobility profiles per country

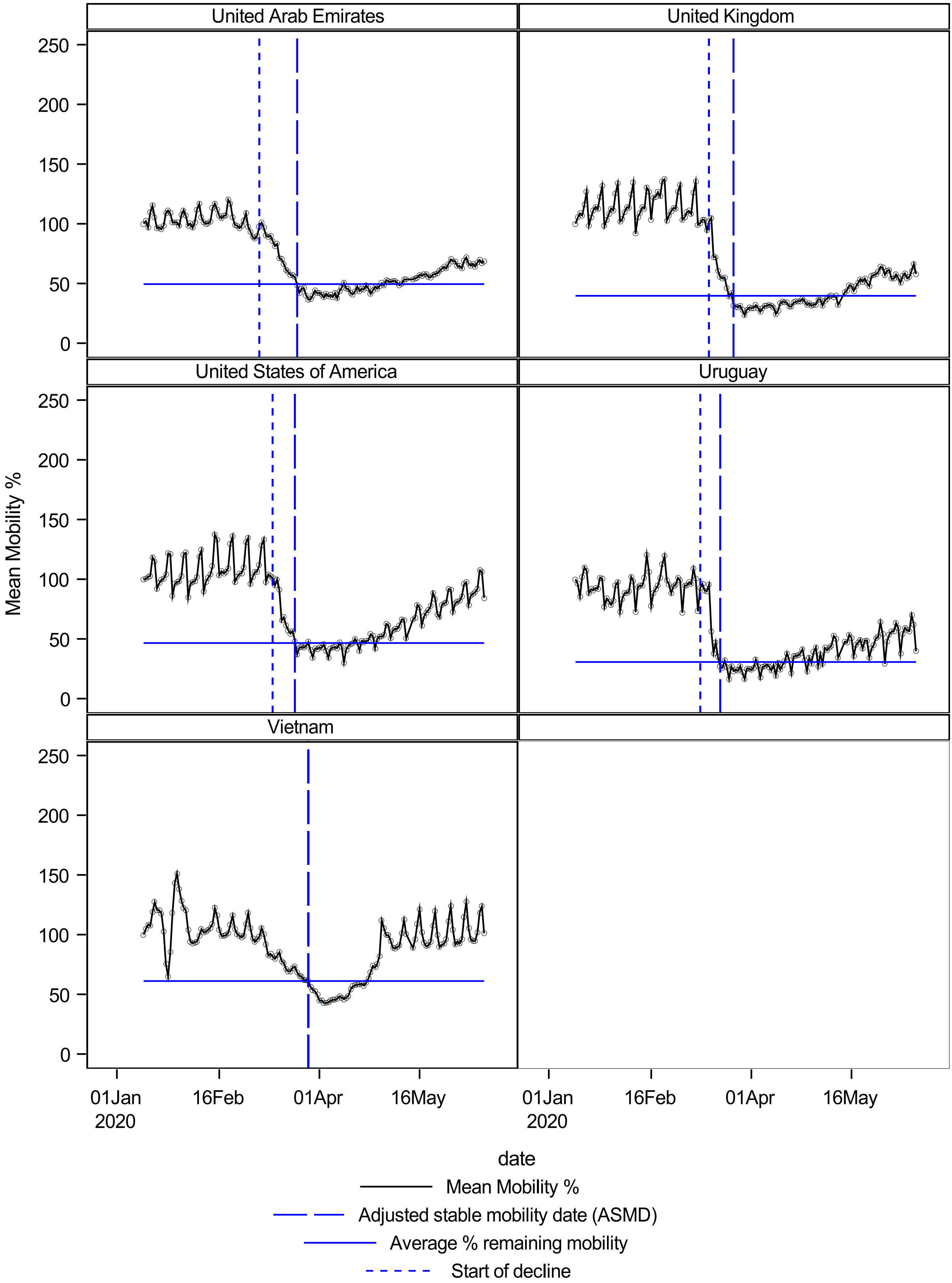

Supplementary Figures 2 Google RAR mobility profiles per country

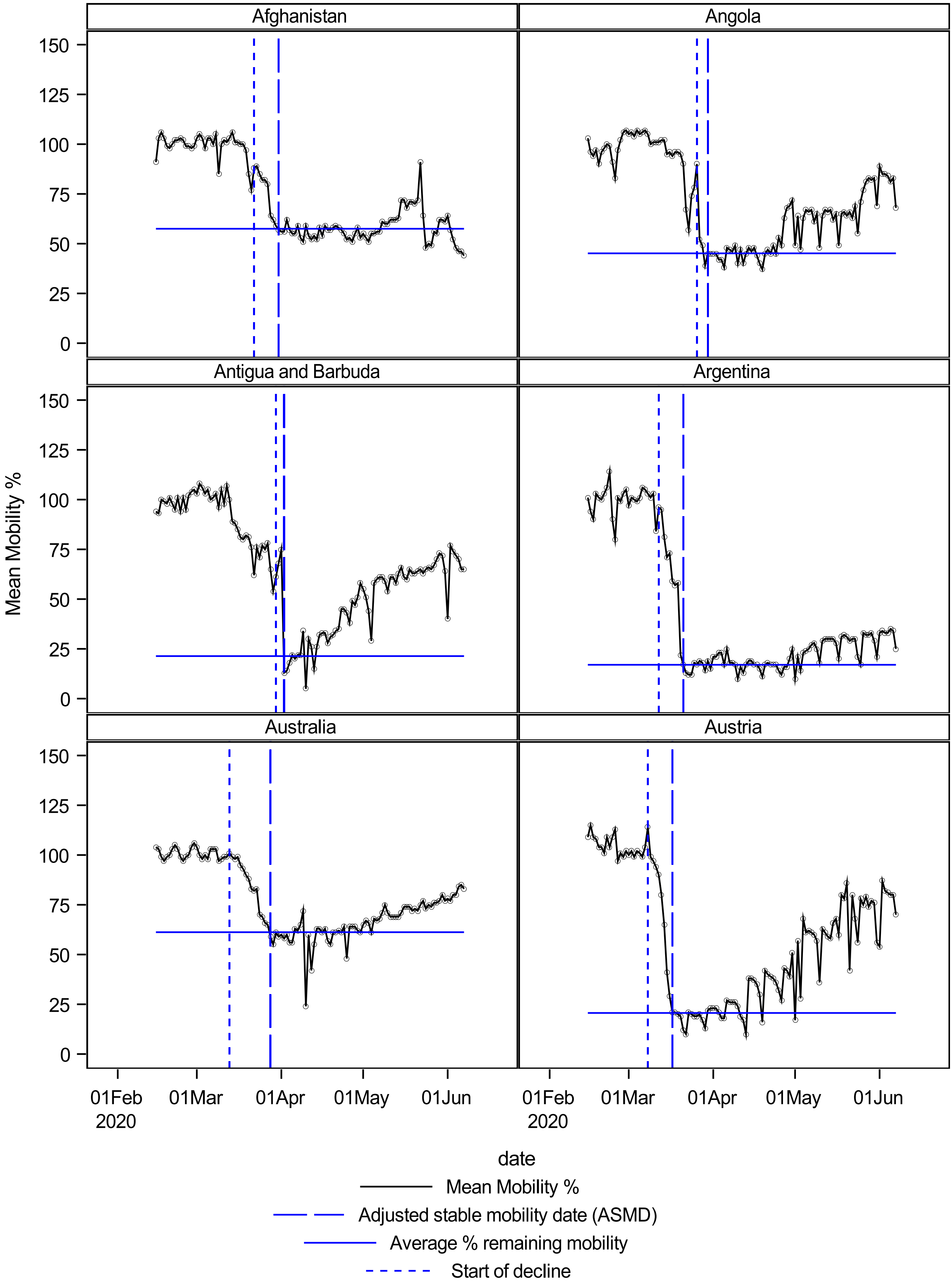

Supplementary Figures 2 Google RAR mobility profiles per country

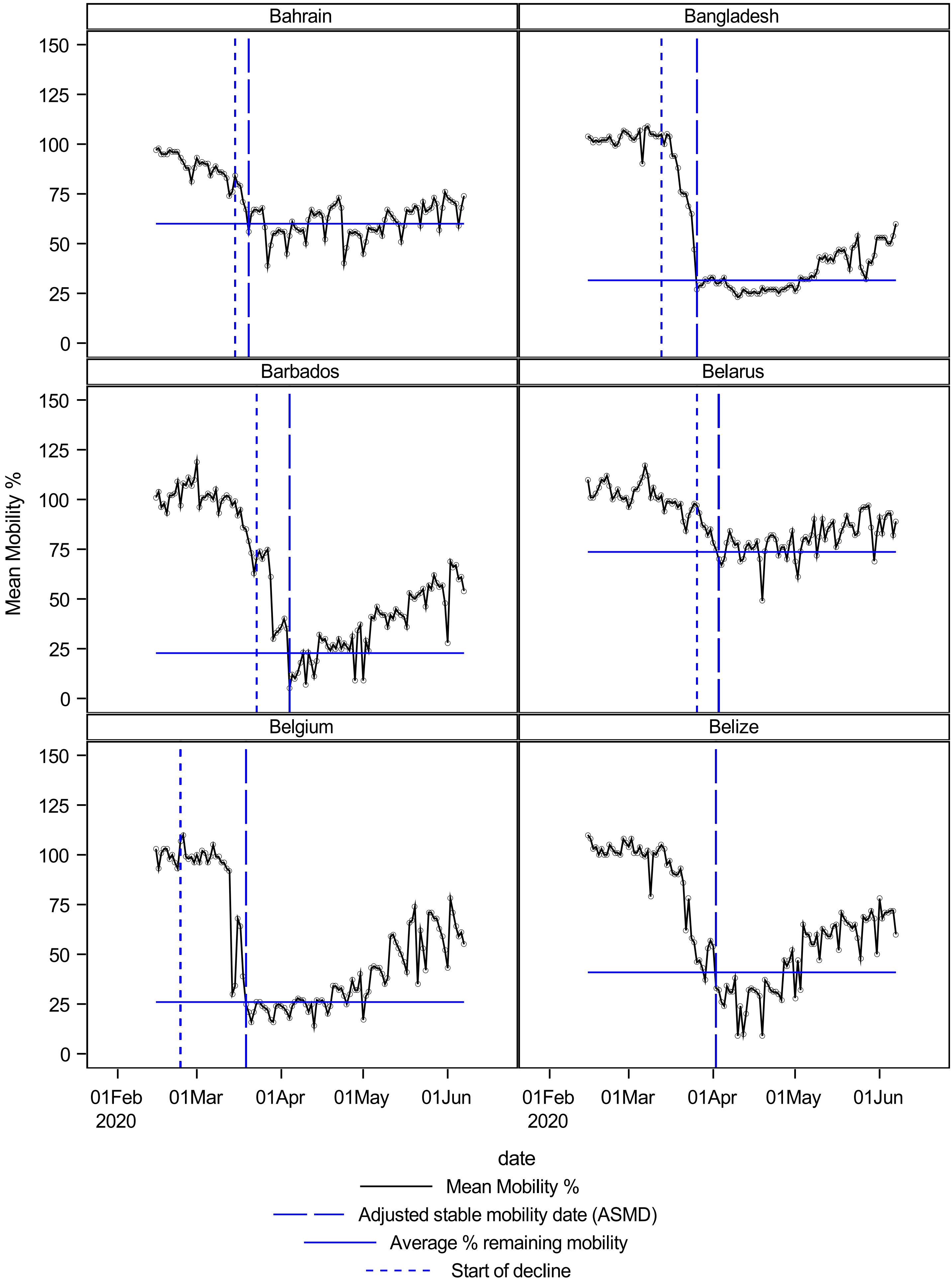

Supplementary Figures 2 Google RAR mobility profiles per country

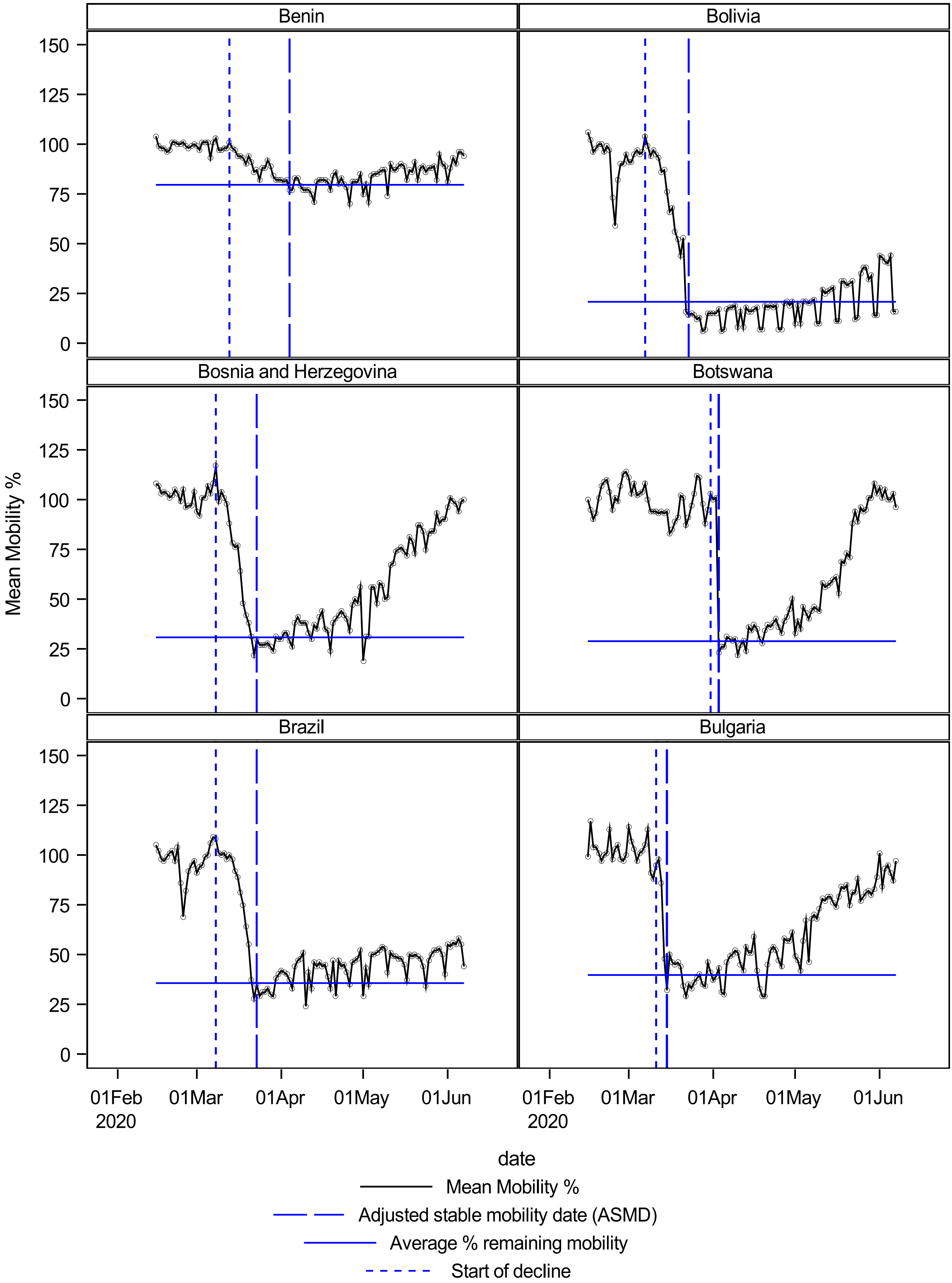

Supplementary Figures 2 Google RAR mobility profiles per country

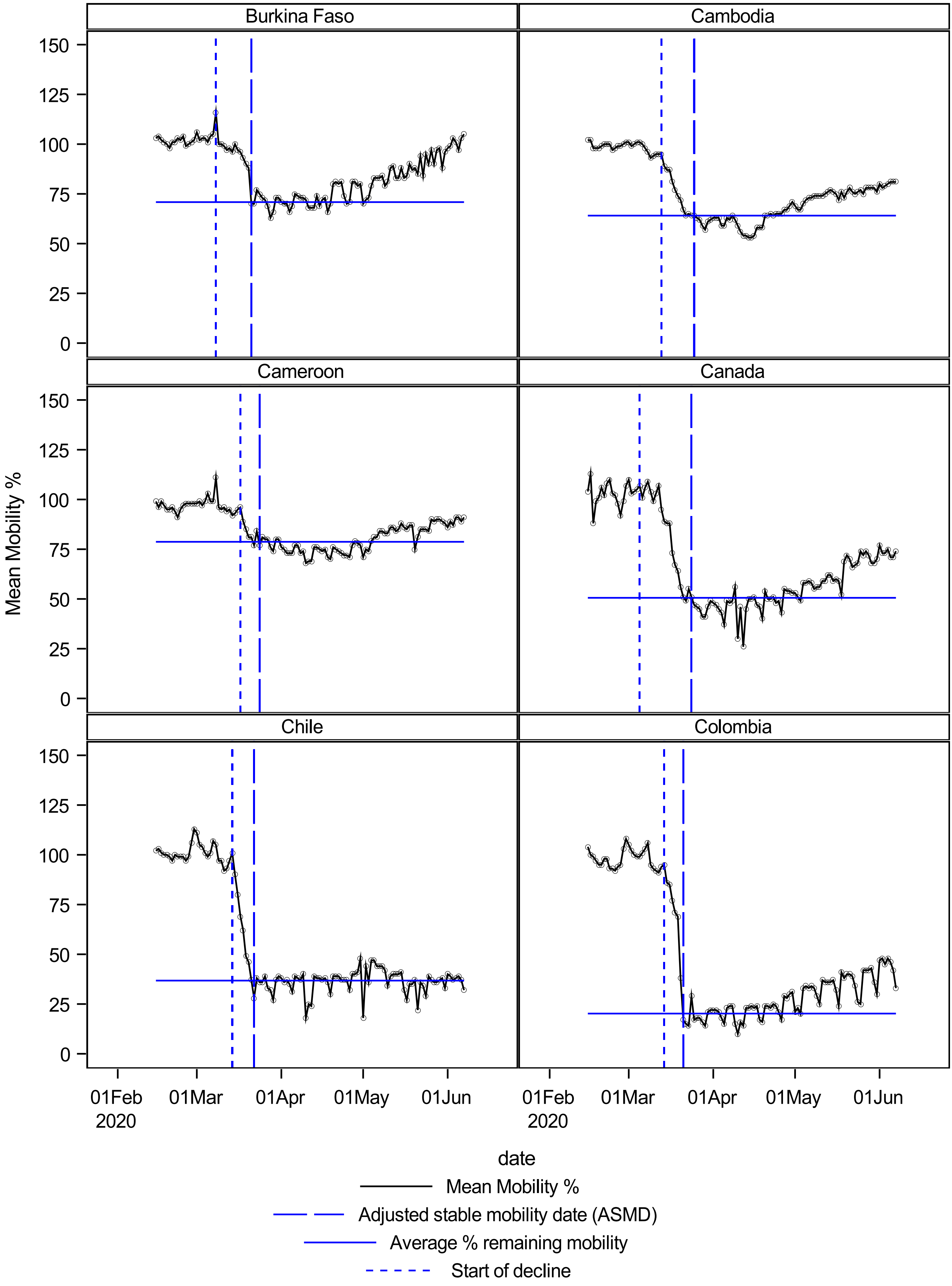

Supplementary Figures 2 Google RAR mobility profiles per country

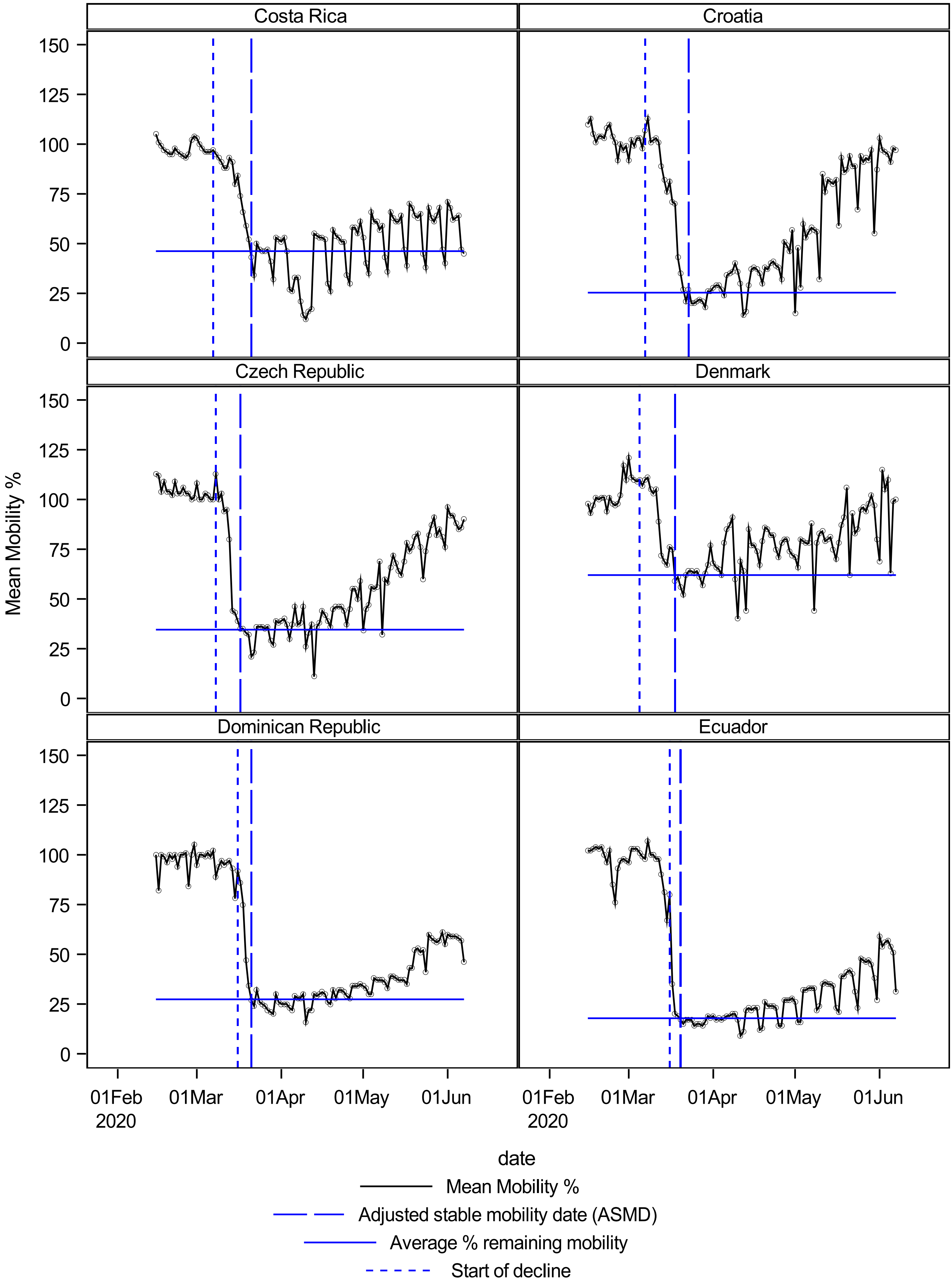

Supplementary Figures 2 Google RAR mobility profiles per country

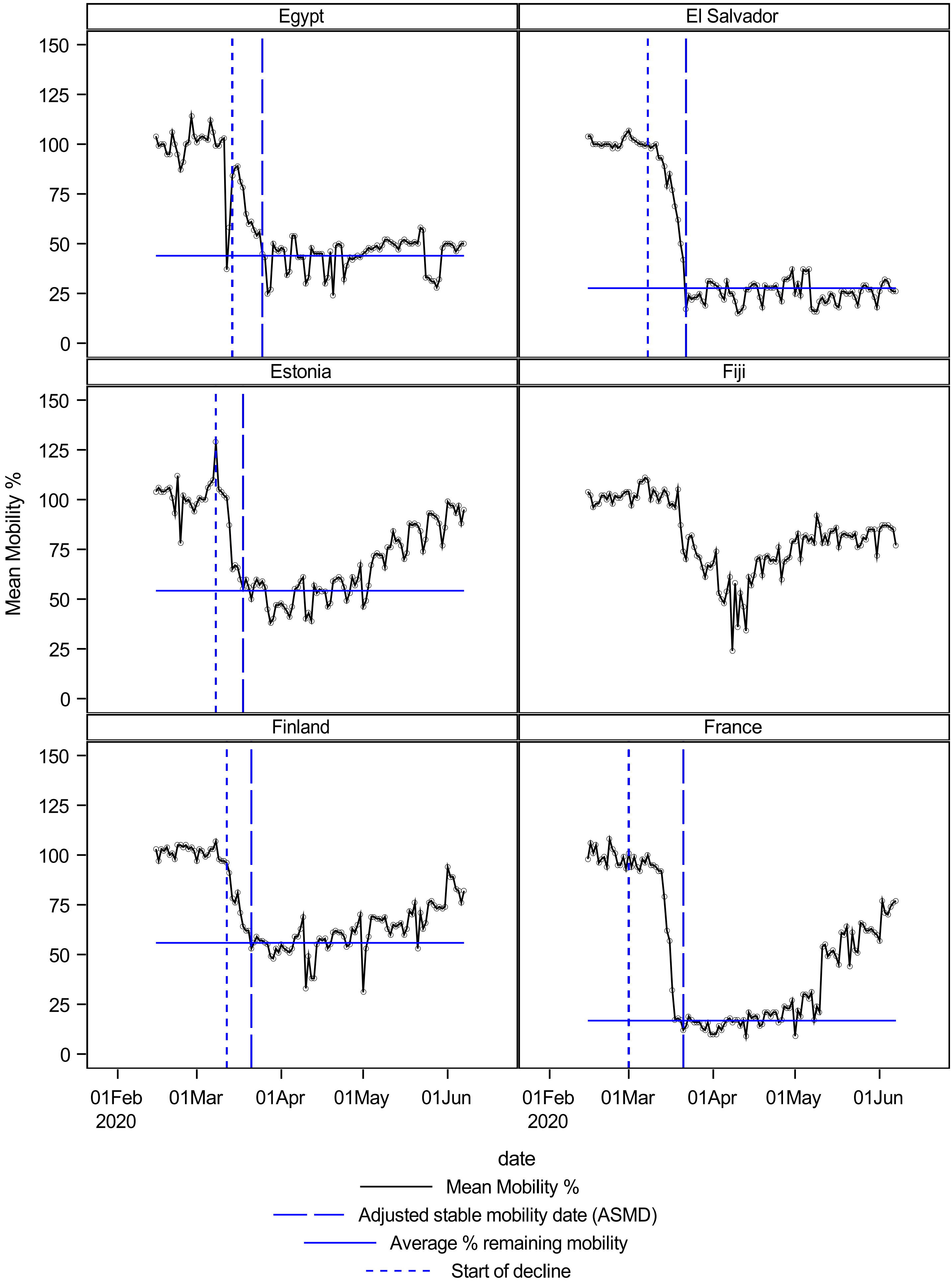

Supplementary Figures 2 Google RAR mobility profiles per country

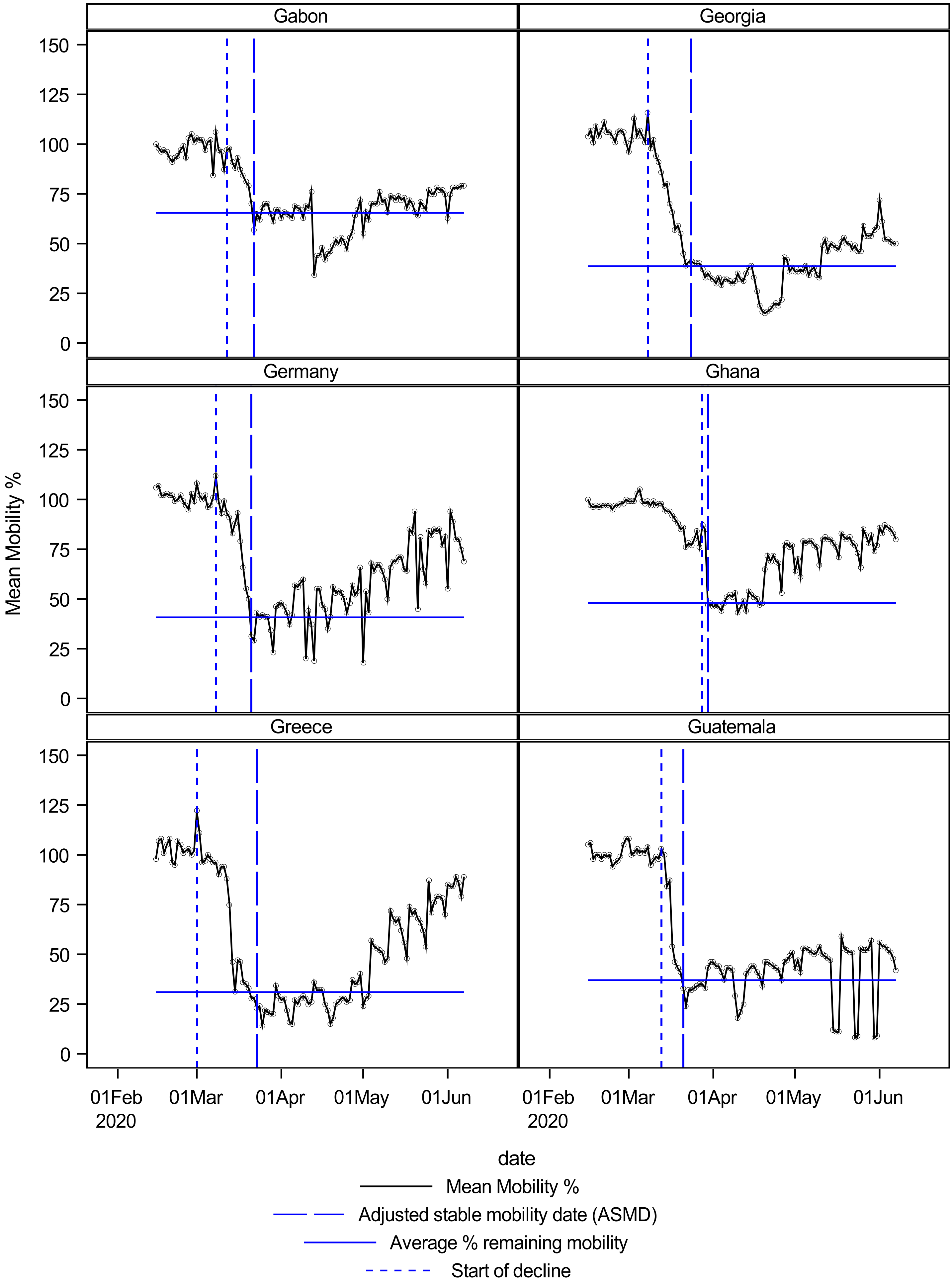

Supplementary Figures 2 Google RAR mobility profiles per country

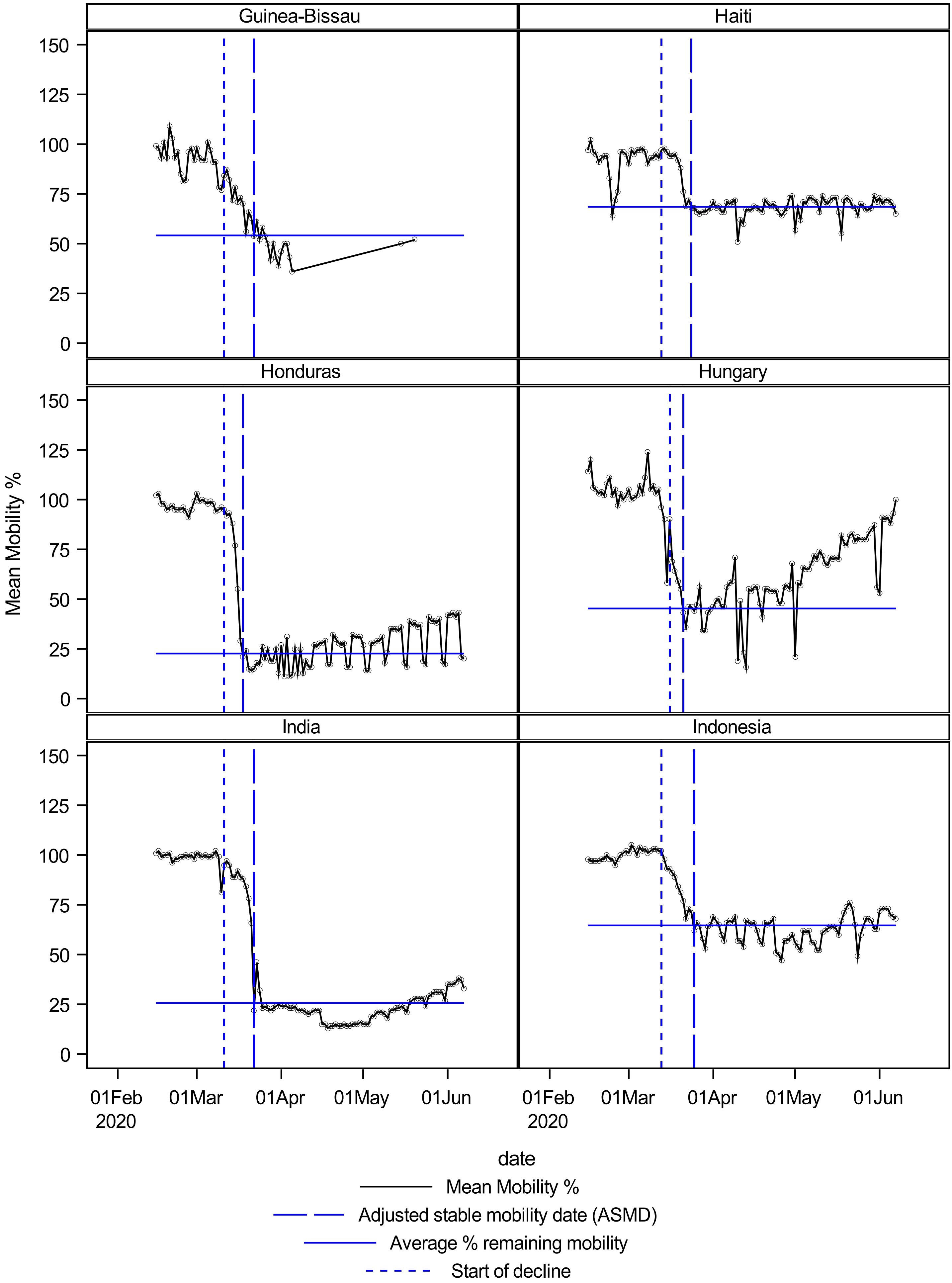

Supplementary Figures 2 Google RAR mobility profiles per country

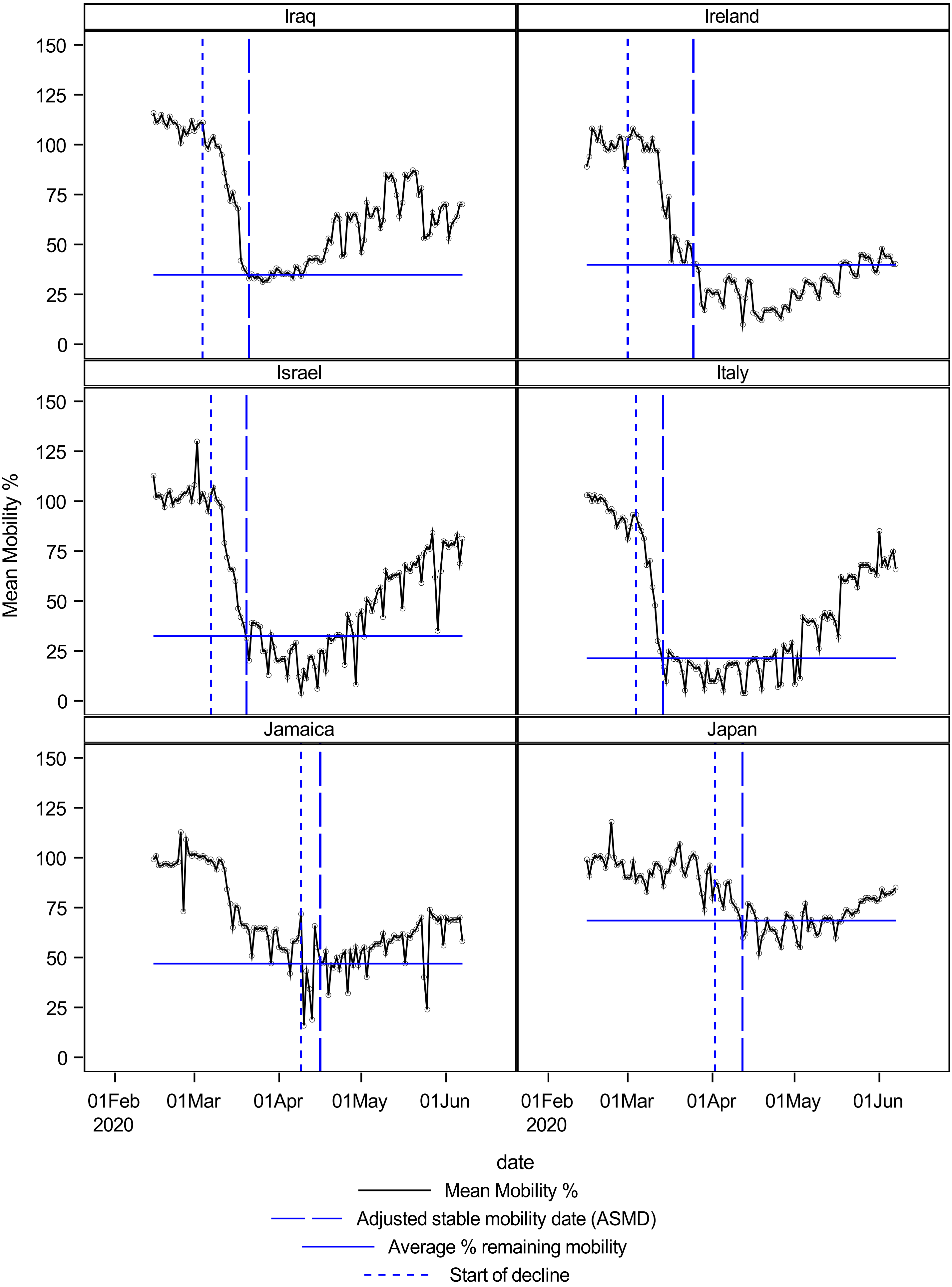

Supplementary Figures 2 Google RAR mobility profiles per country

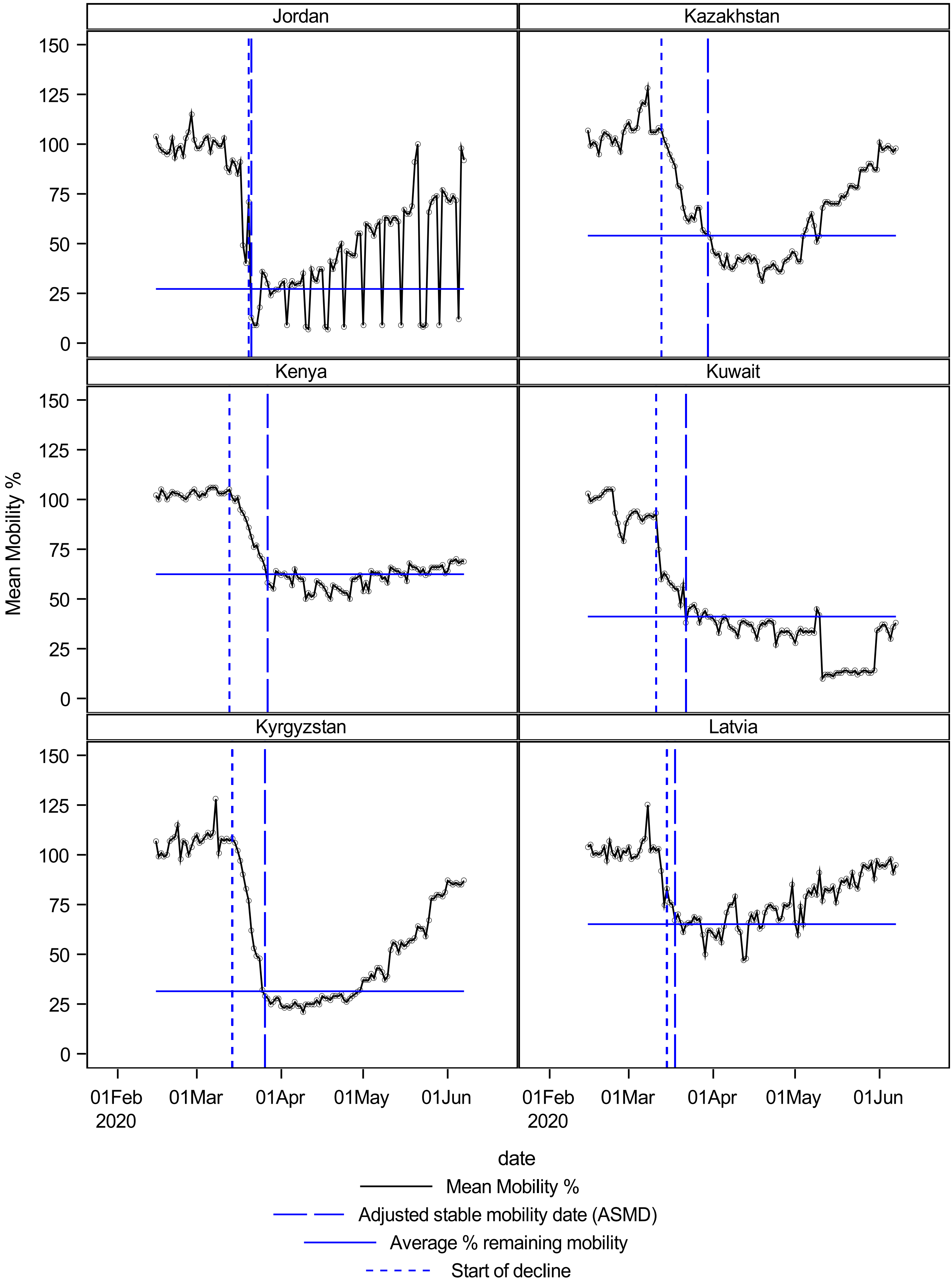

Supplementary Figures 2 Google RAR mobility profiles per country

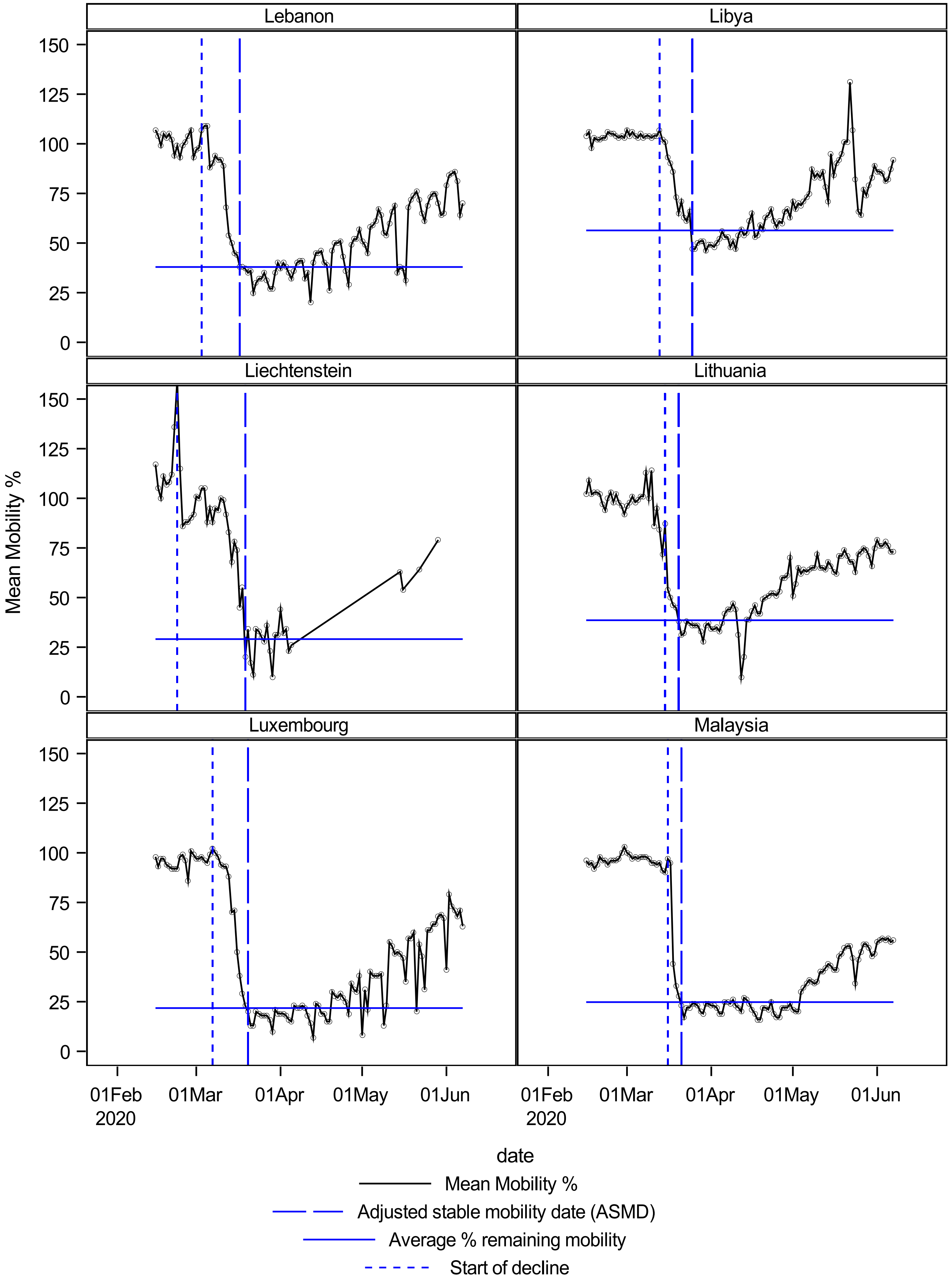

Supplementary Figures 2 Google RAR mobility profiles per country

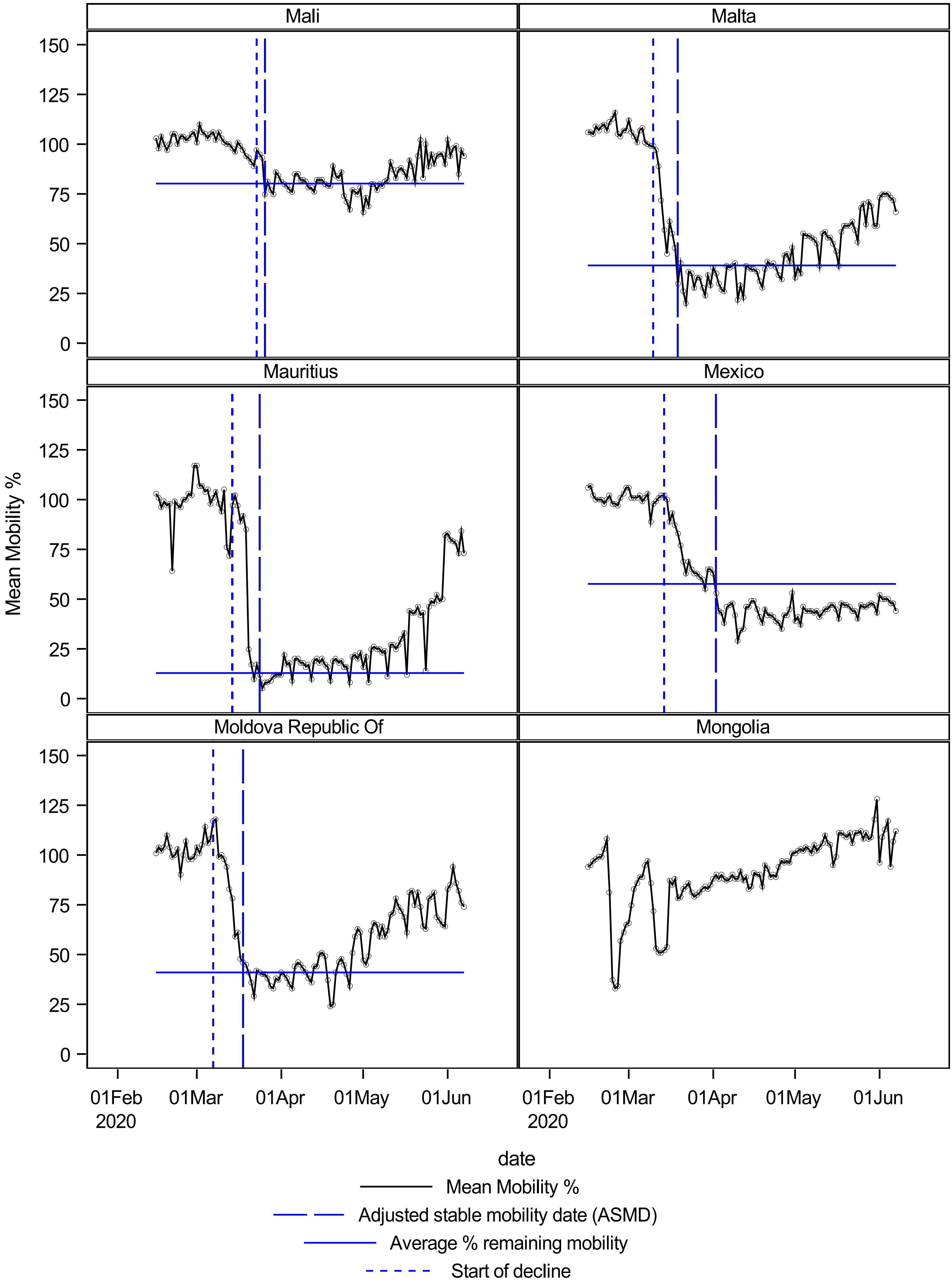

Supplementary Figures 2 Google RAR mobility profiles per country

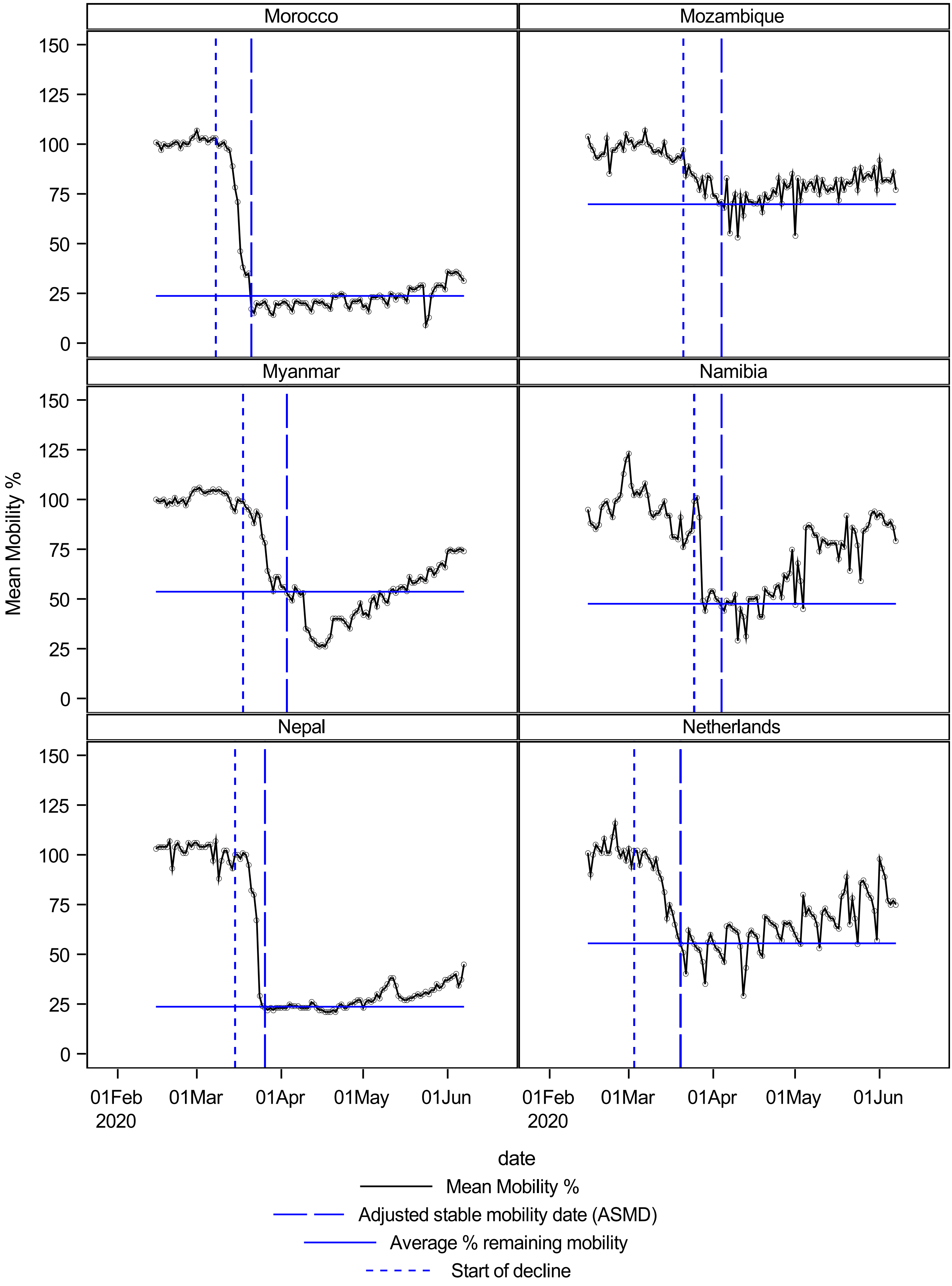

Supplementary Figures 2 Google RAR mobility profiles per country

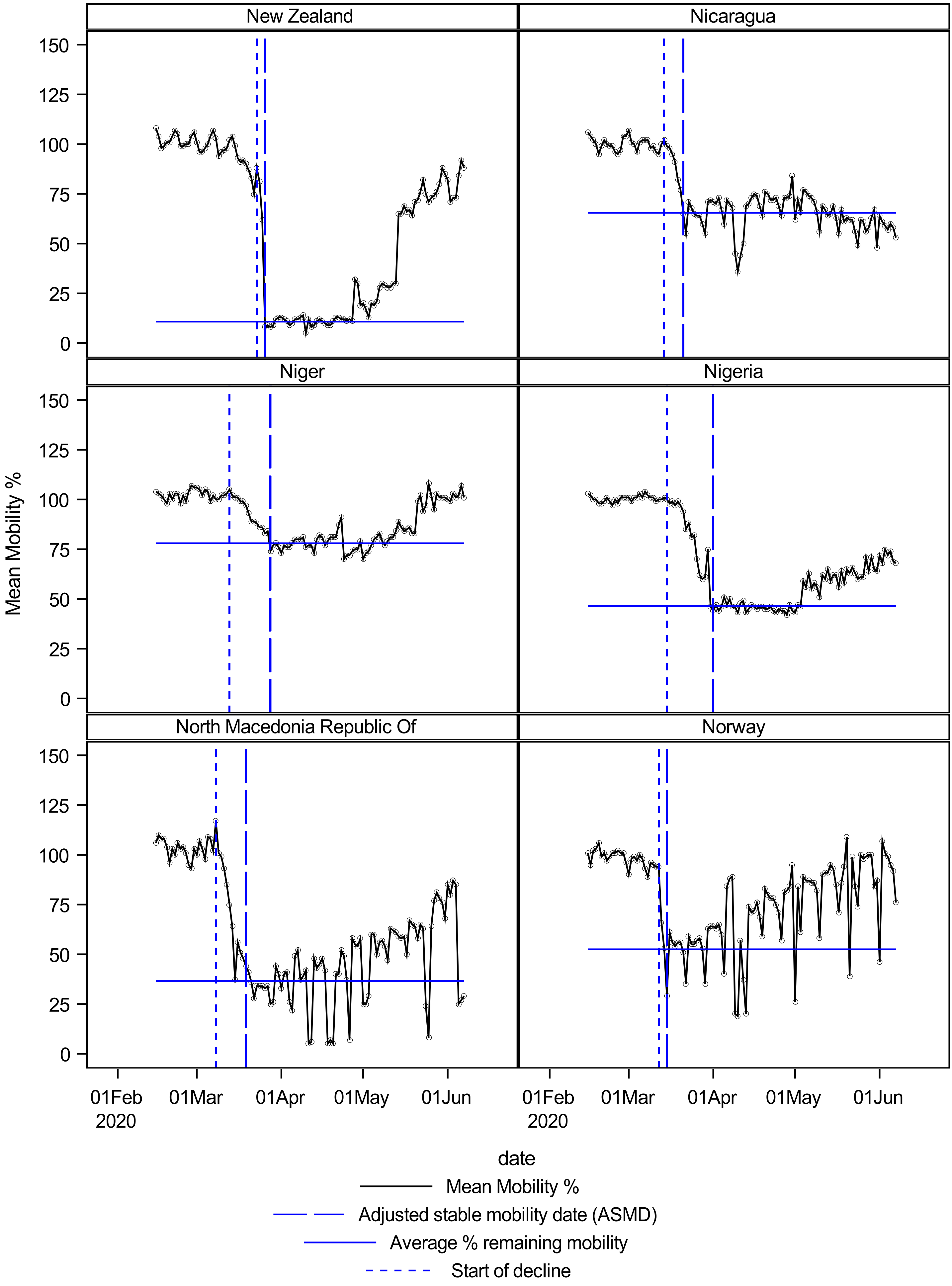

Supplementary Figures 2 Google RAR mobility profiles per country

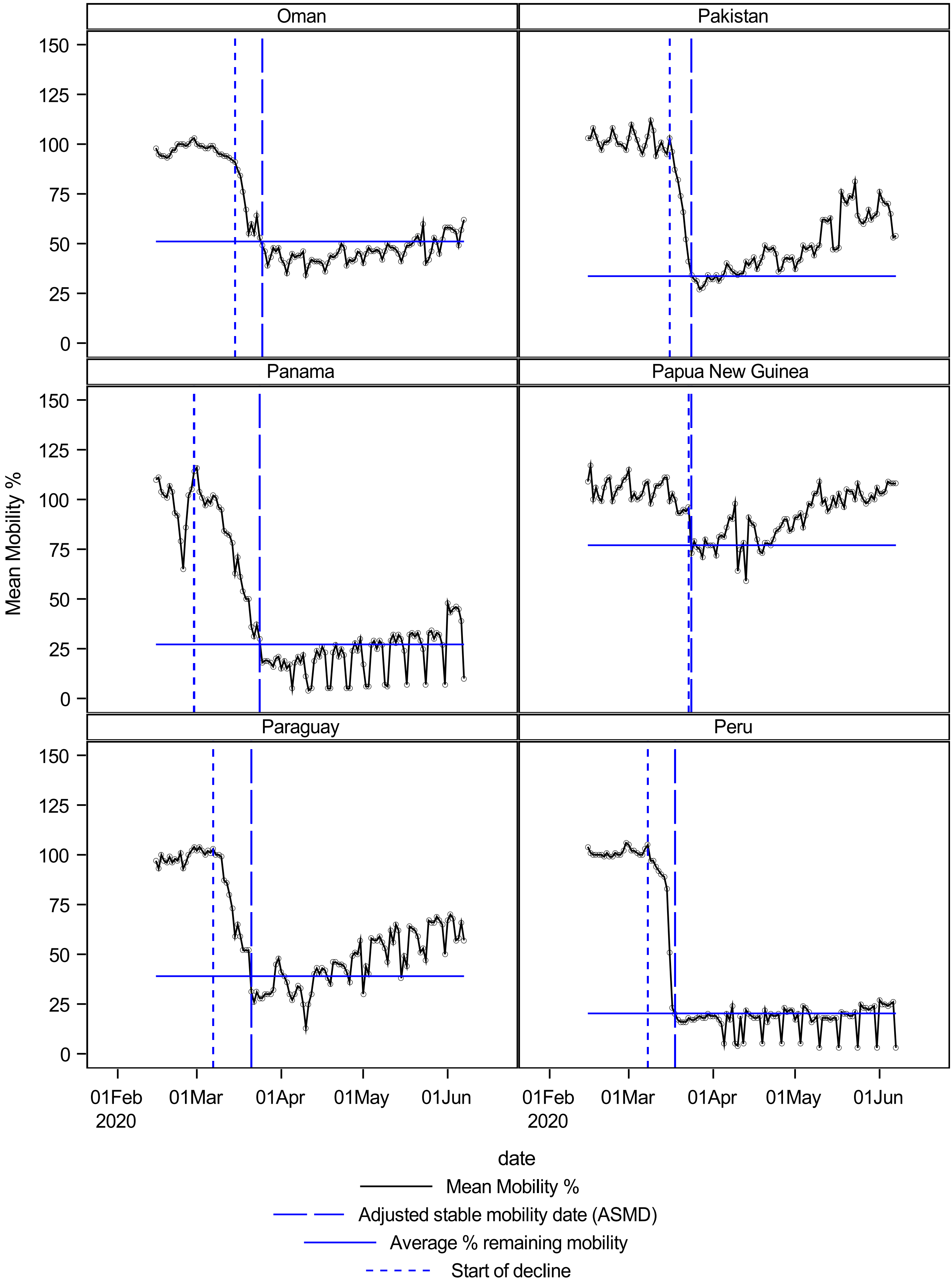

Supplementary Figures 2 Google RAR mobility profiles per country

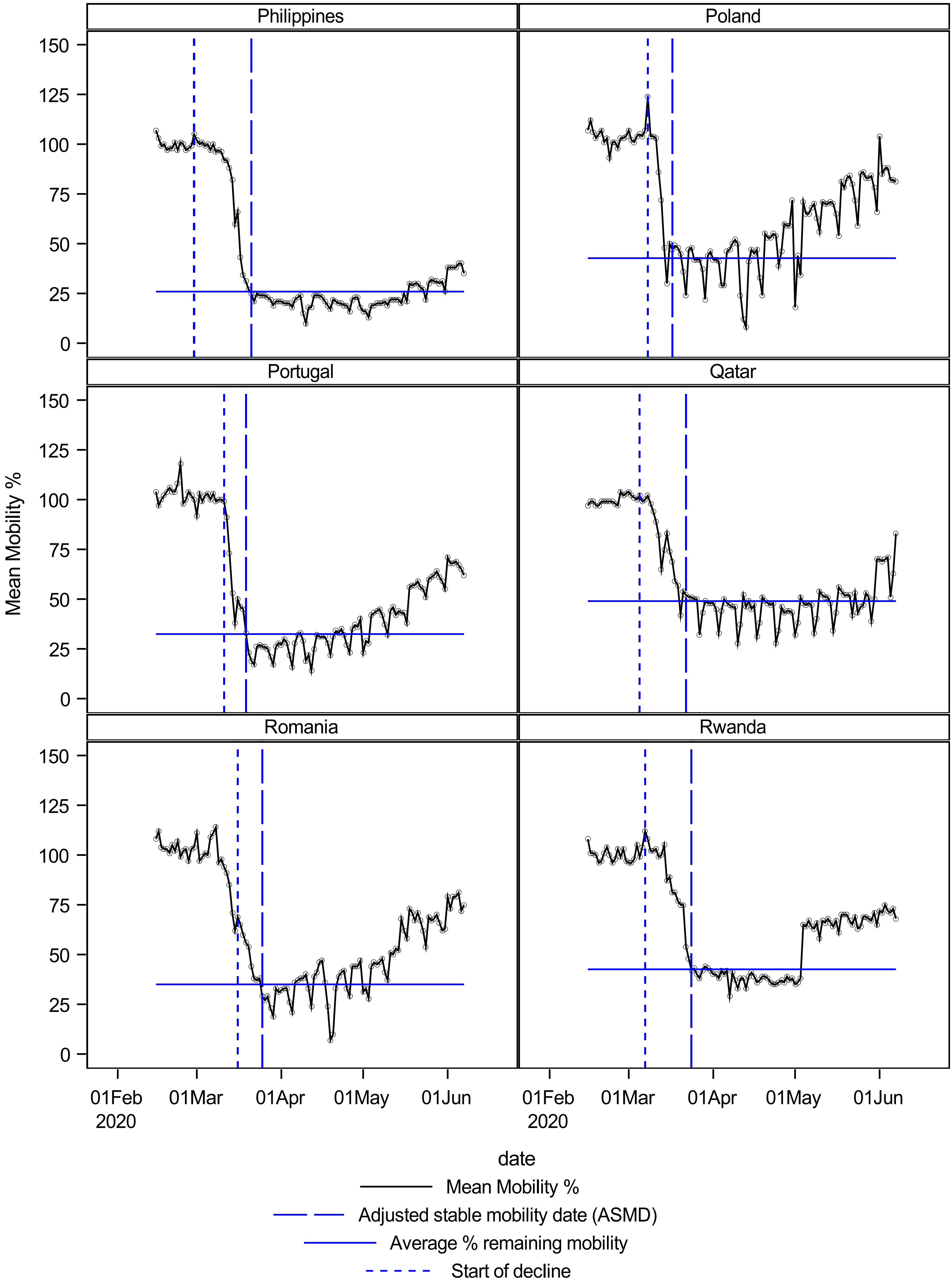

Supplementary Figures 2 Google RAR mobility profiles per country

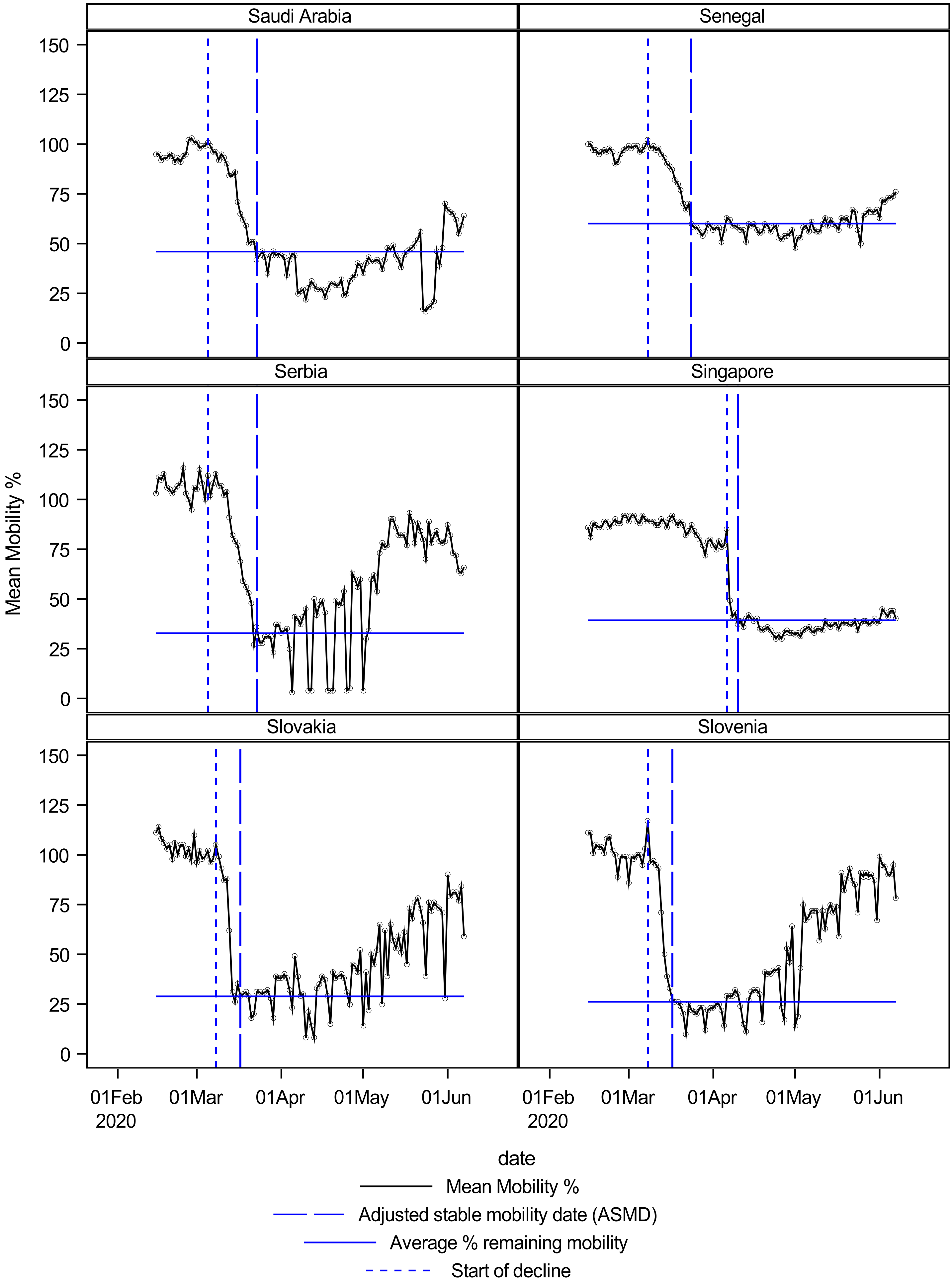

Supplementary Figures 2 Google RAR mobility profiles per country

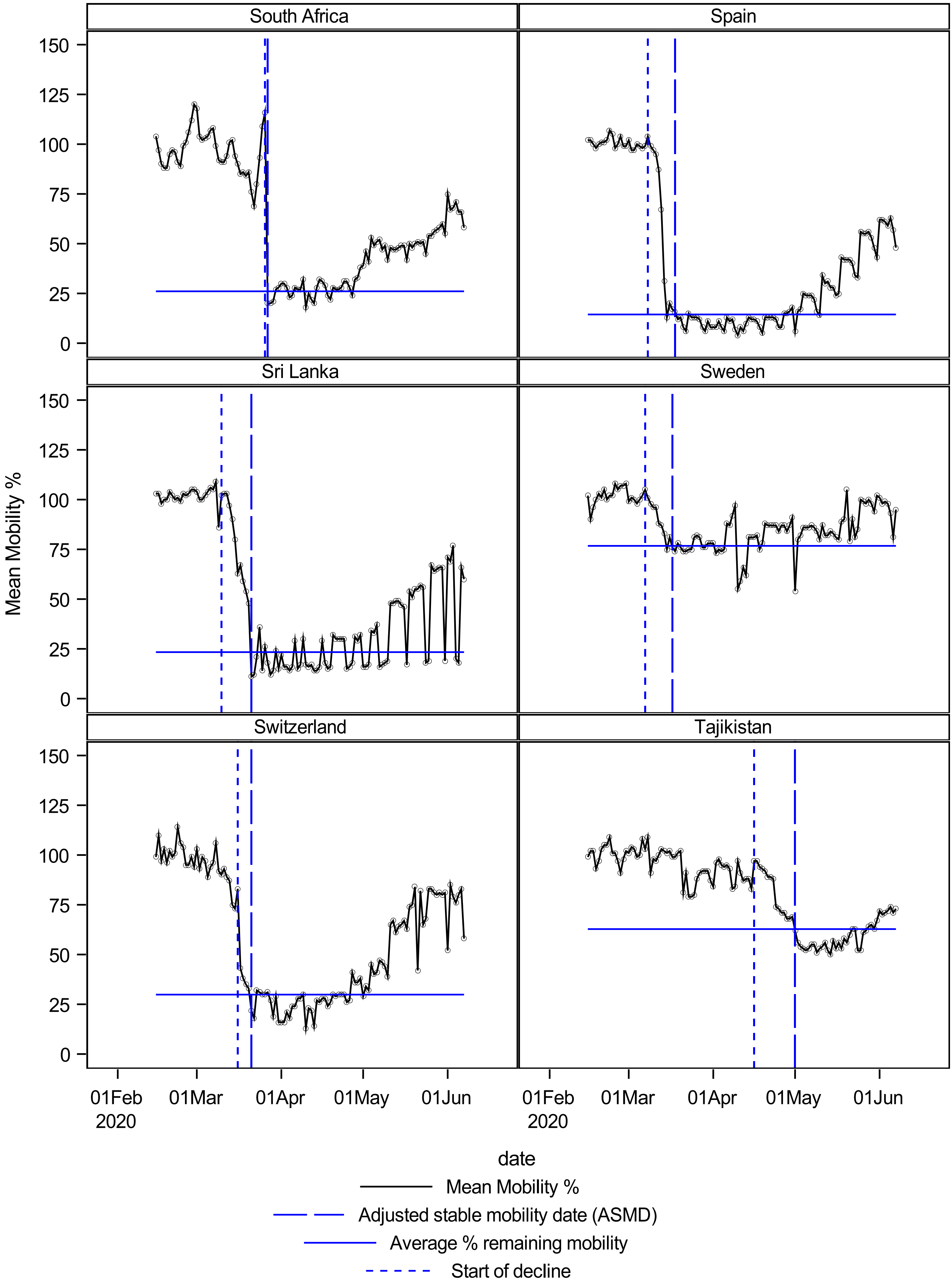

Supplementary Figures 2 Google RAR mobility profiles per country

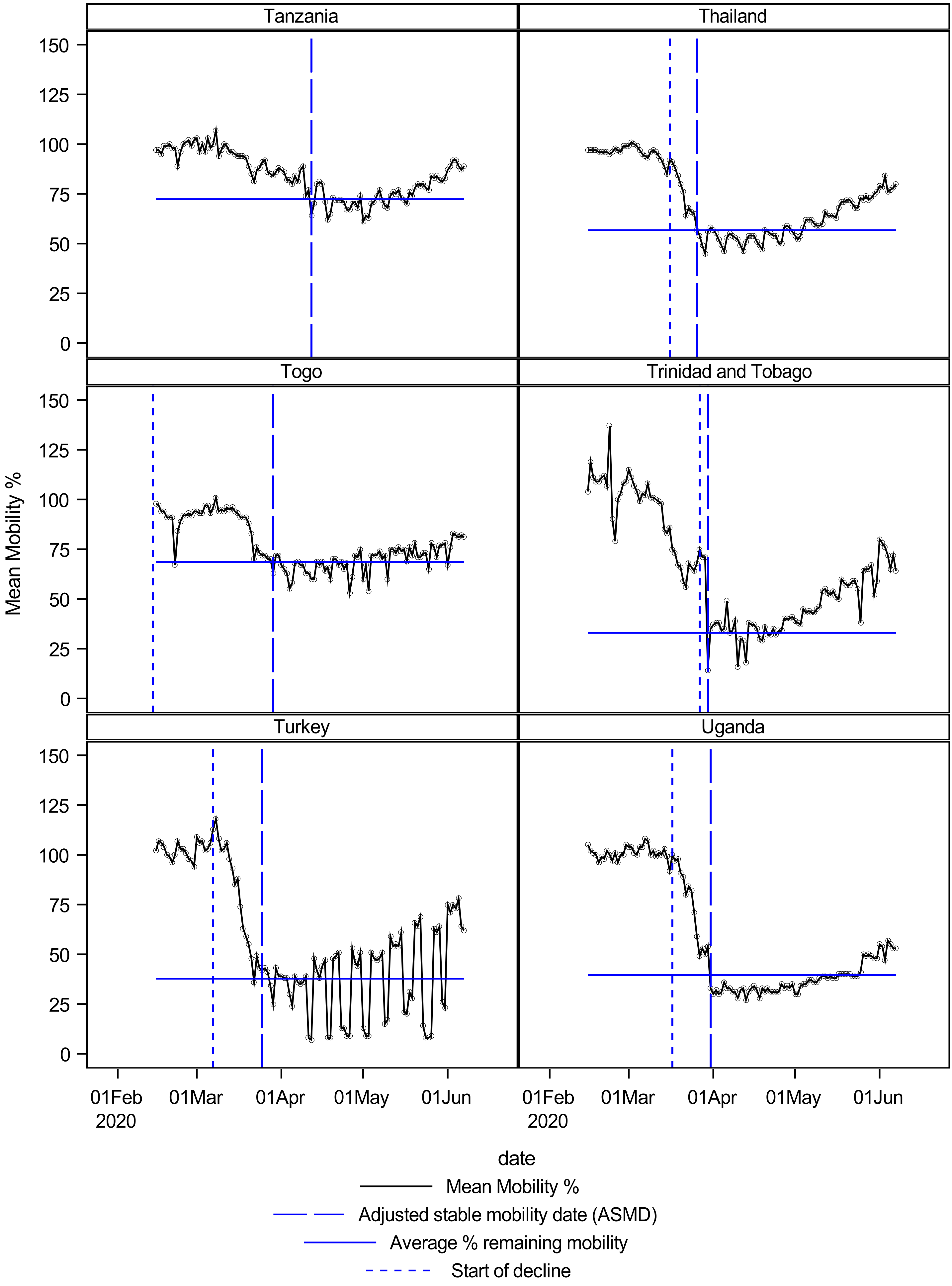

Supplementary Figures 2 Google RAR mobility profiles per country

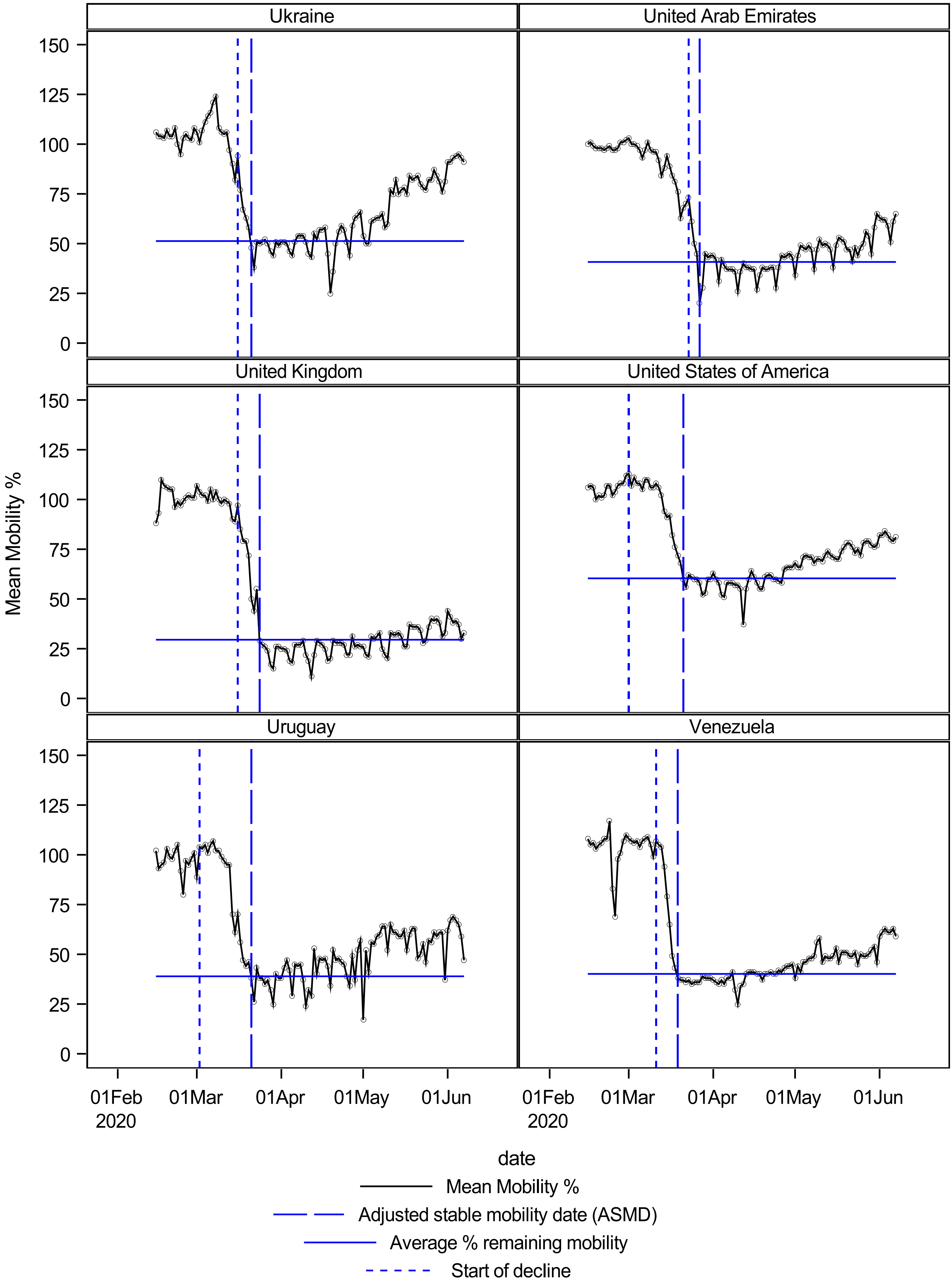

Supplementary Figures 2 Google RAR mobility profiles per country

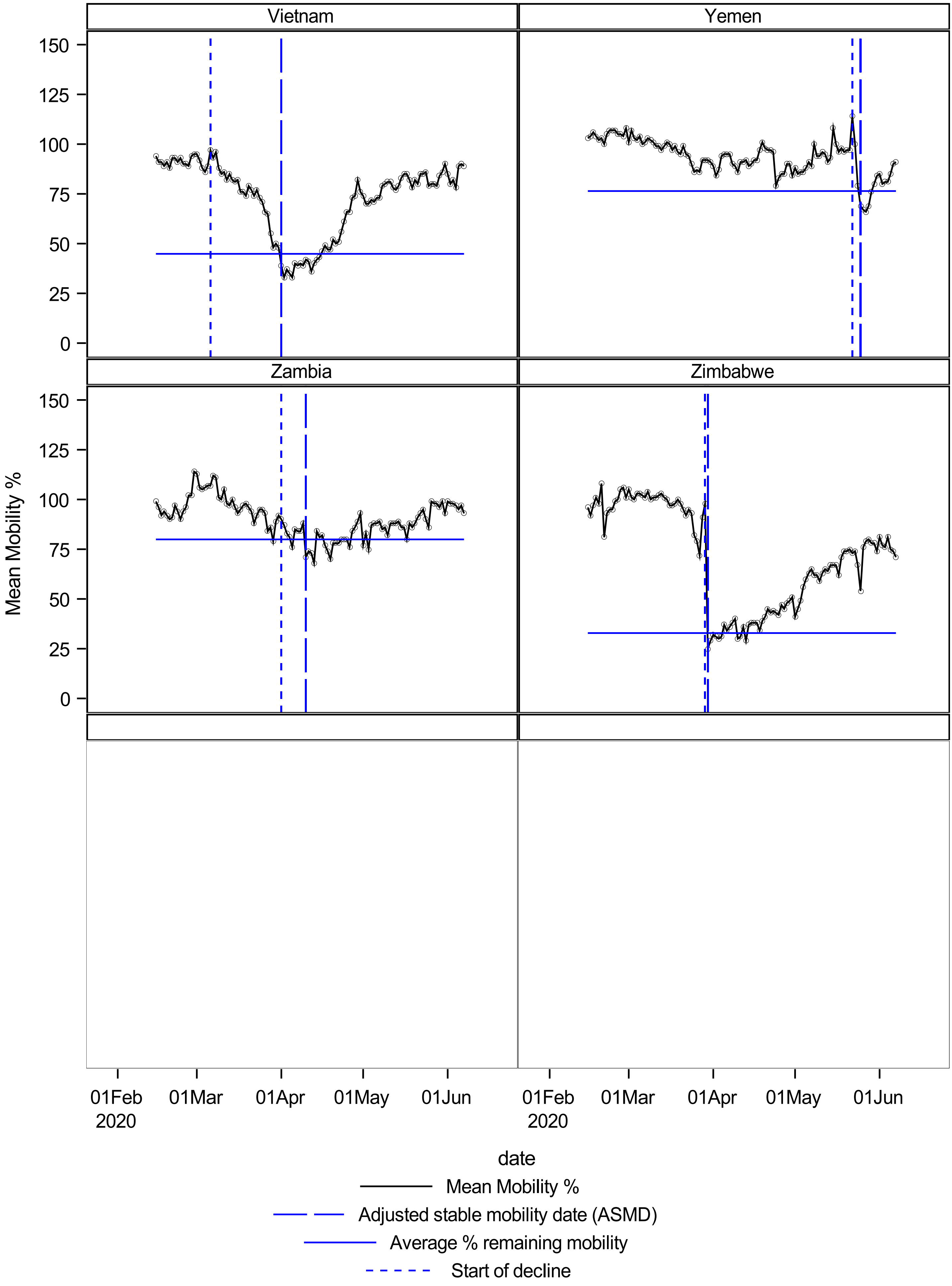

Supplementary Figures 3 Google TS mobility profiles per country

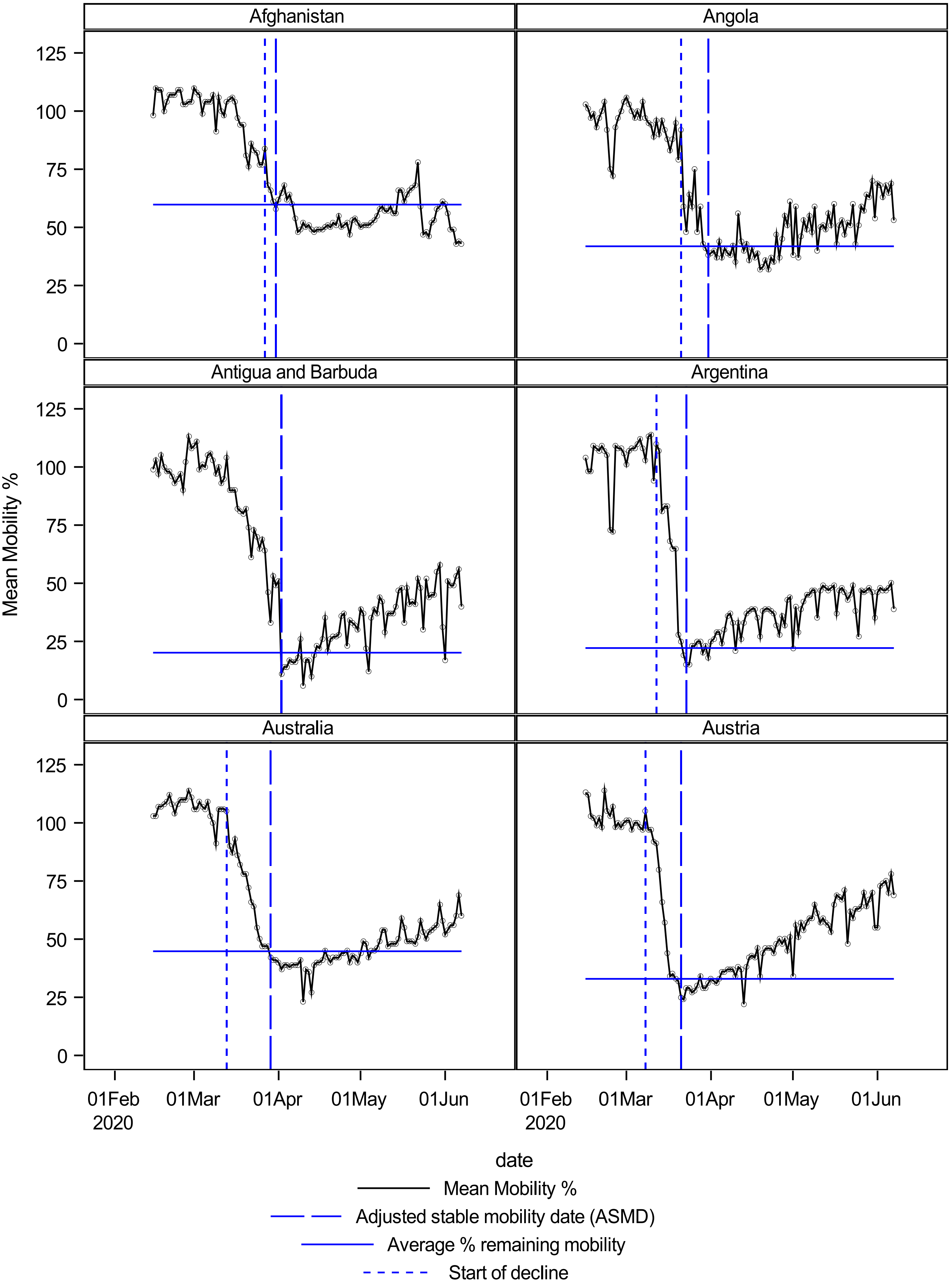

Supplementary Figures 3 Google TS mobility profiles per country

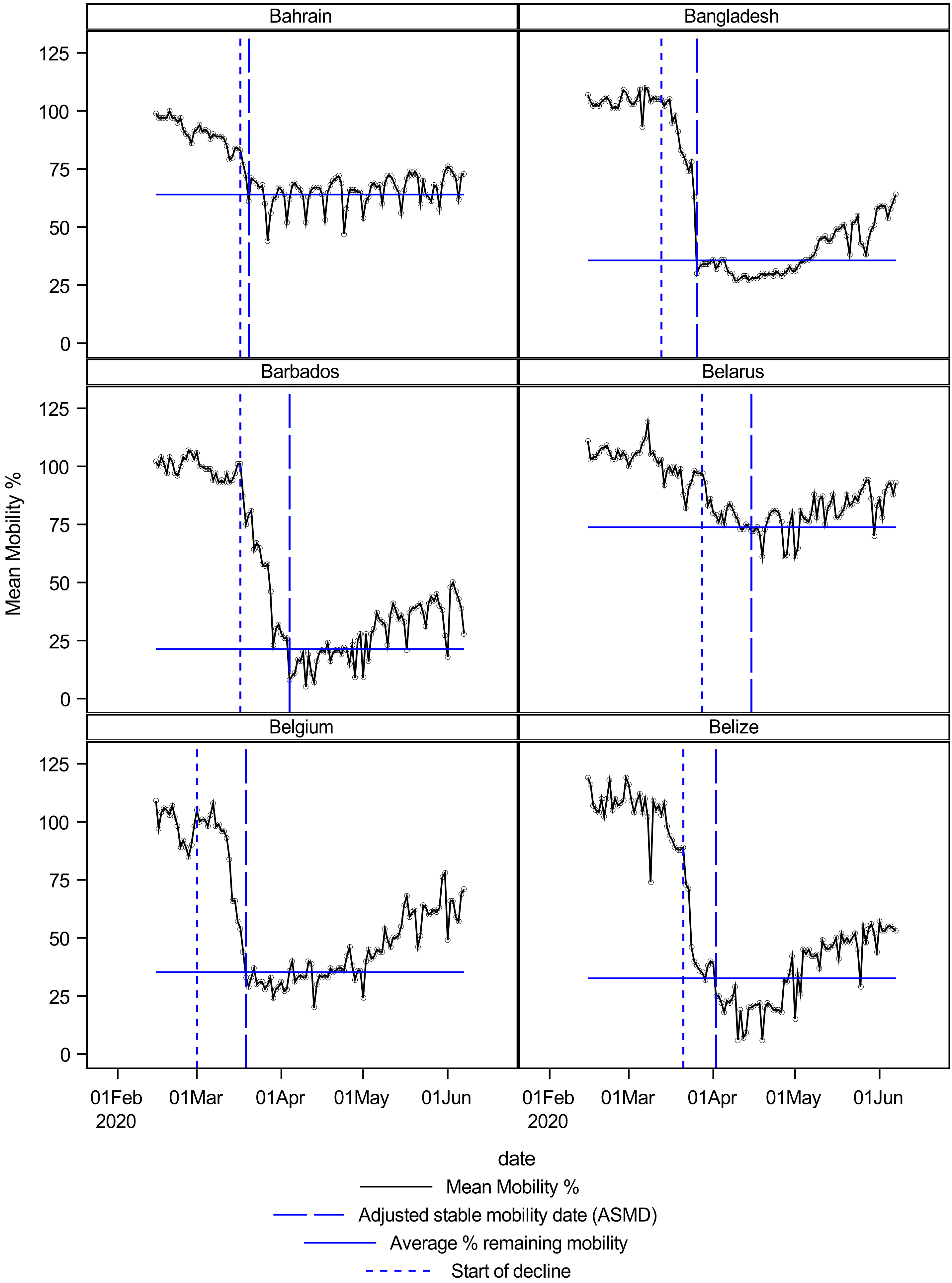

Supplementary Figures 3 Google TS mobility profiles per country

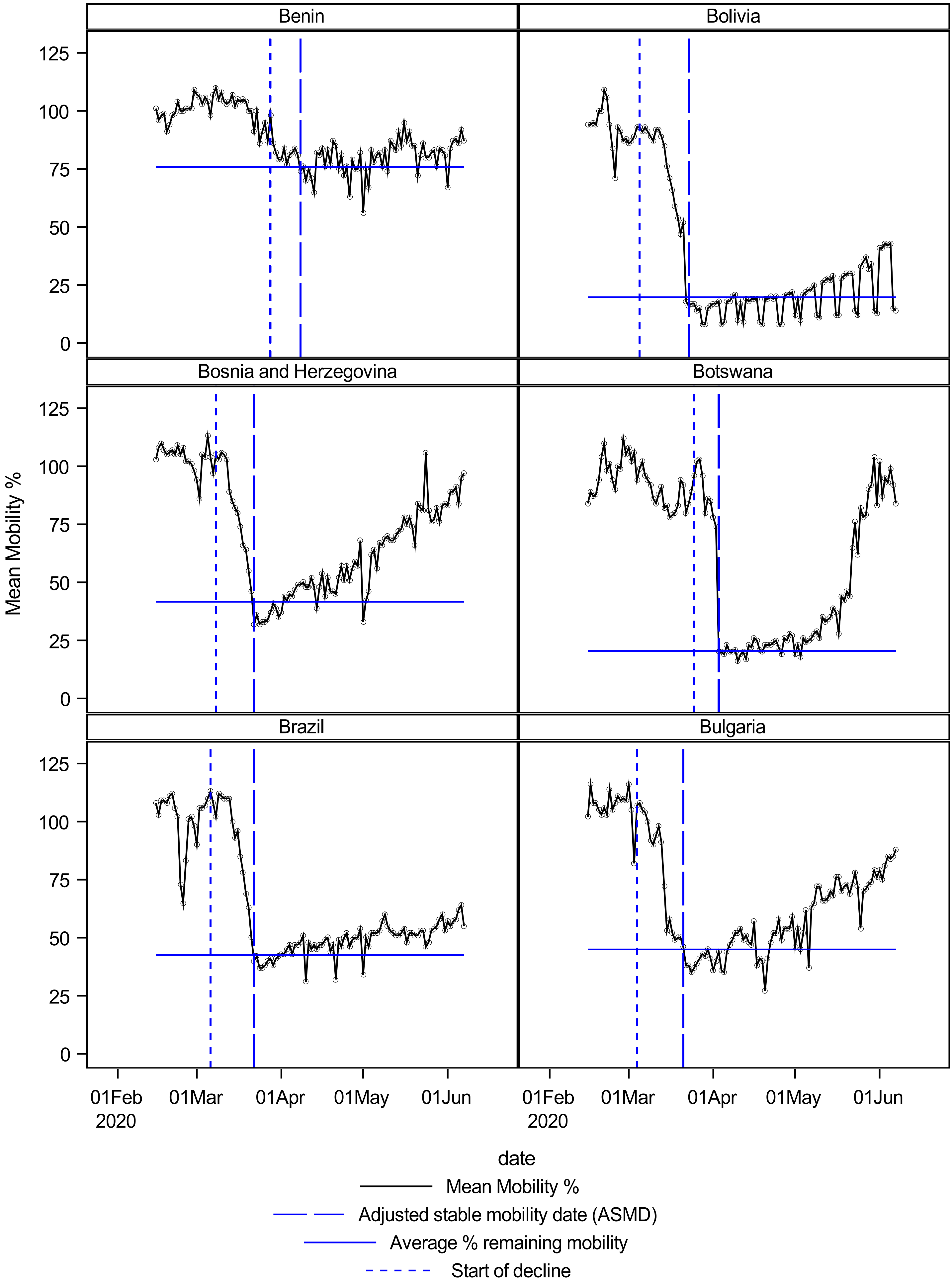

Supplementary Figures 3 Google TS mobility profiles per country

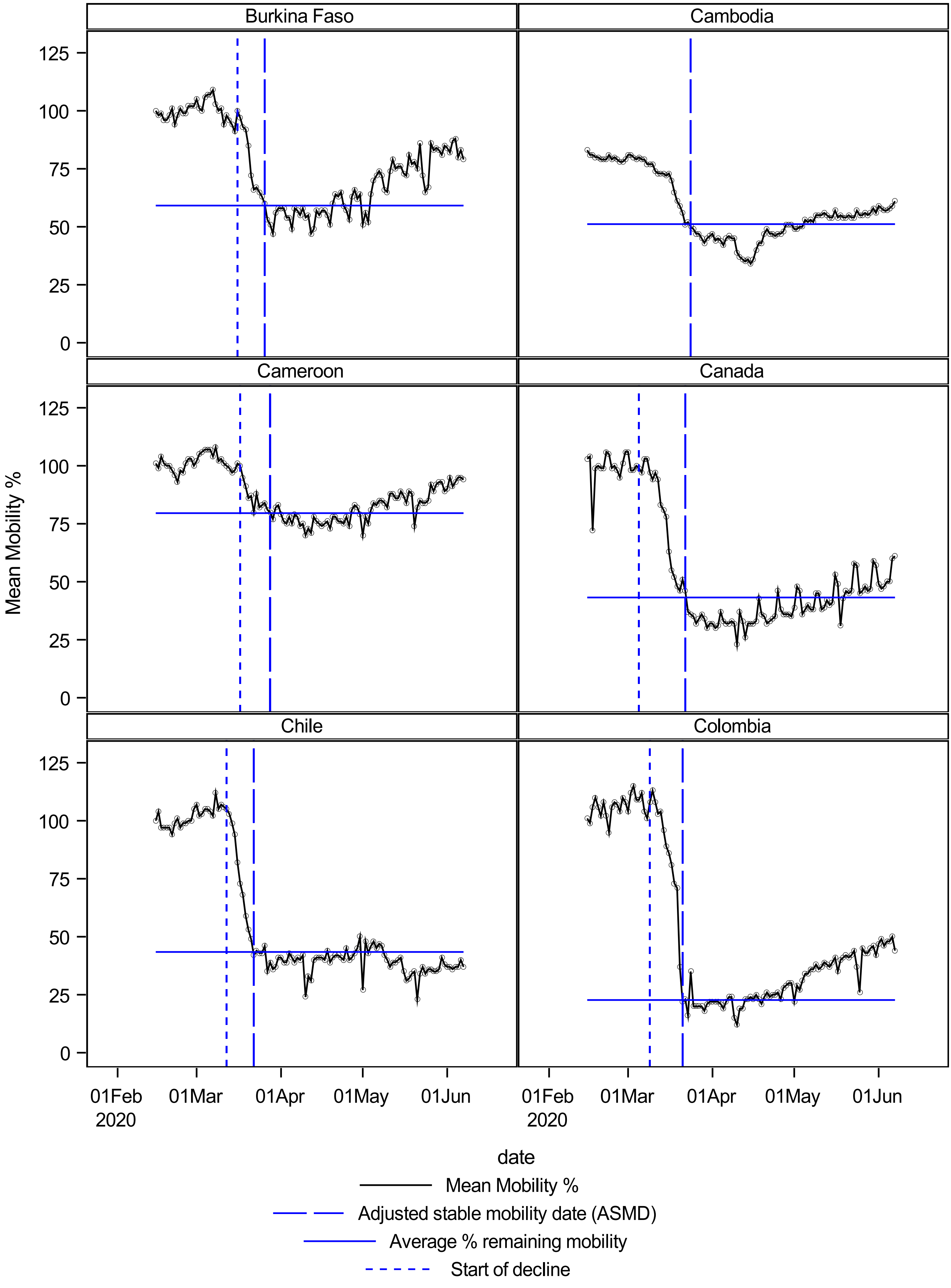

Supplementary Figures 3 Google TS mobility profiles per country

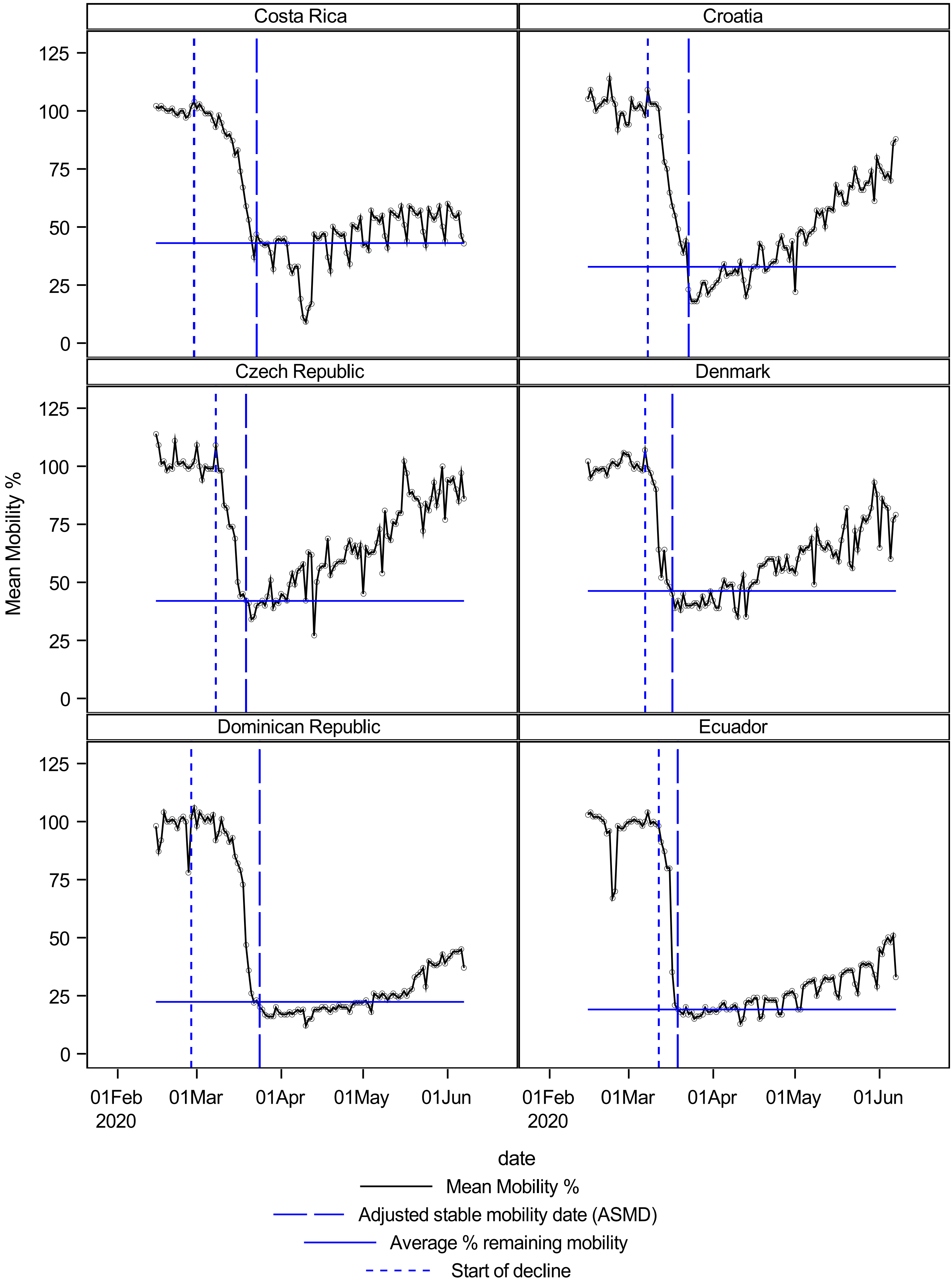

Supplementary Figures 3 Google TS mobility profiles per country

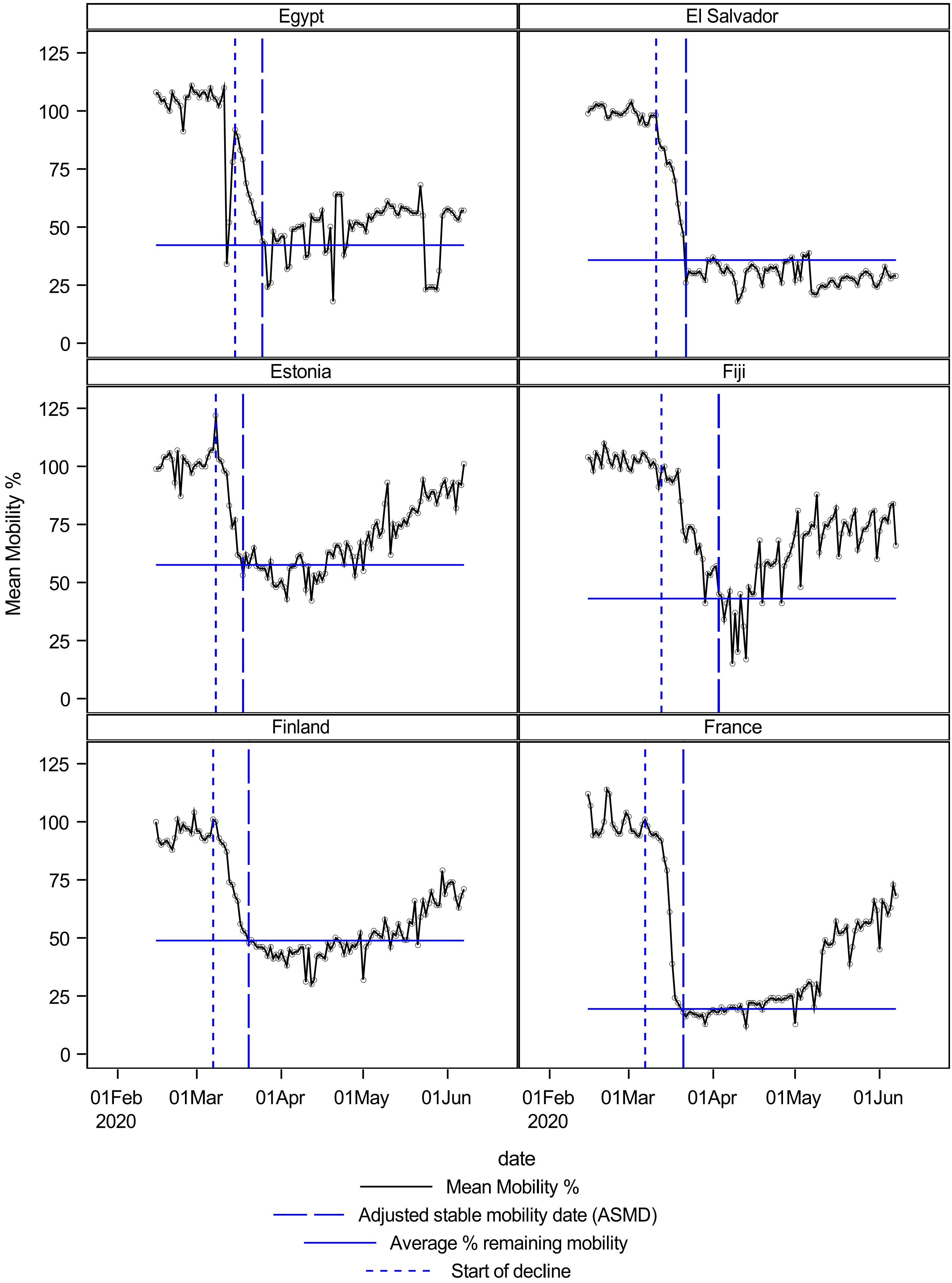

Supplementary Figures 3 Google TS mobility profiles per country

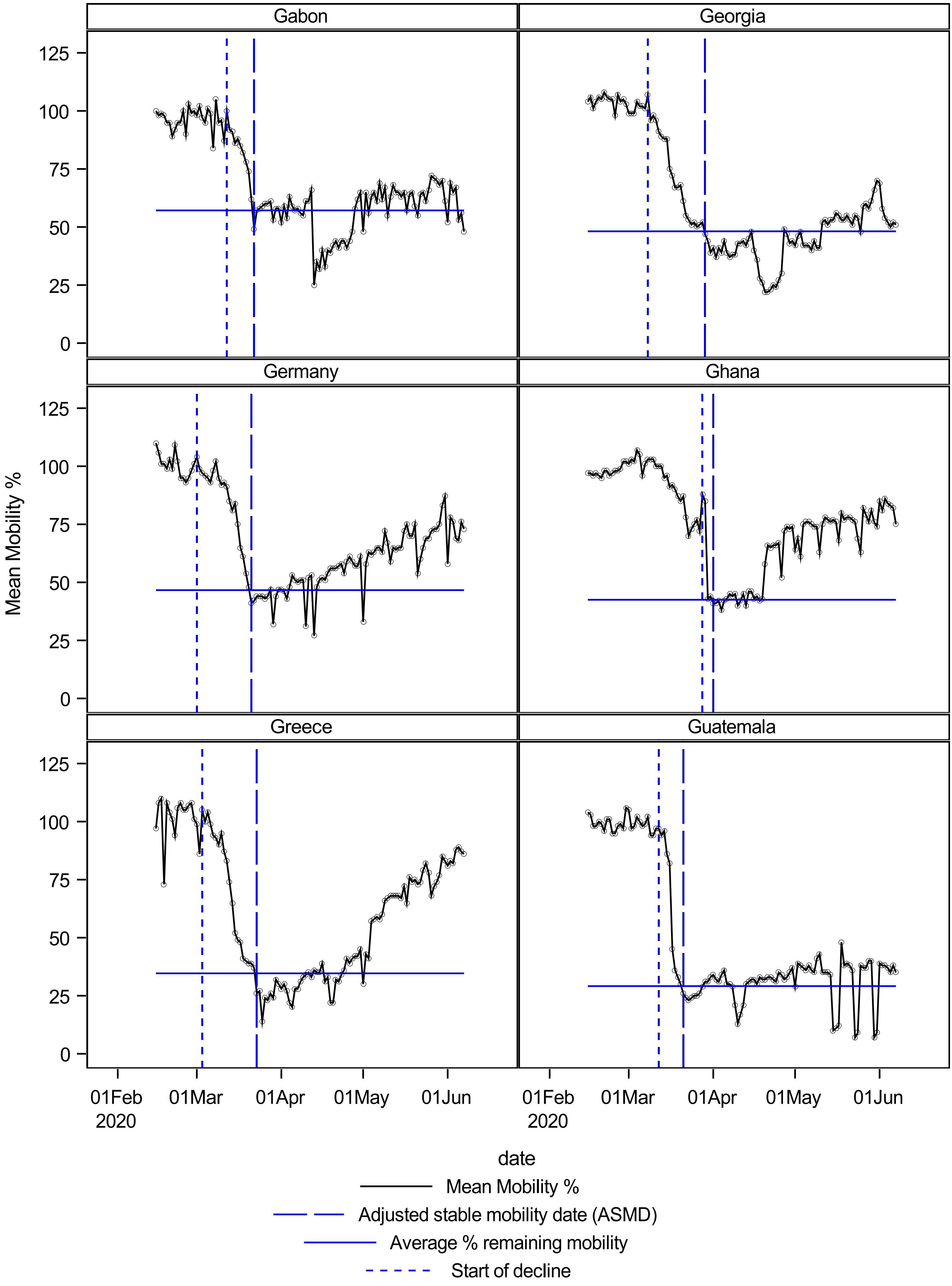

Supplementary Figures 3 Google TS mobility profiles per country

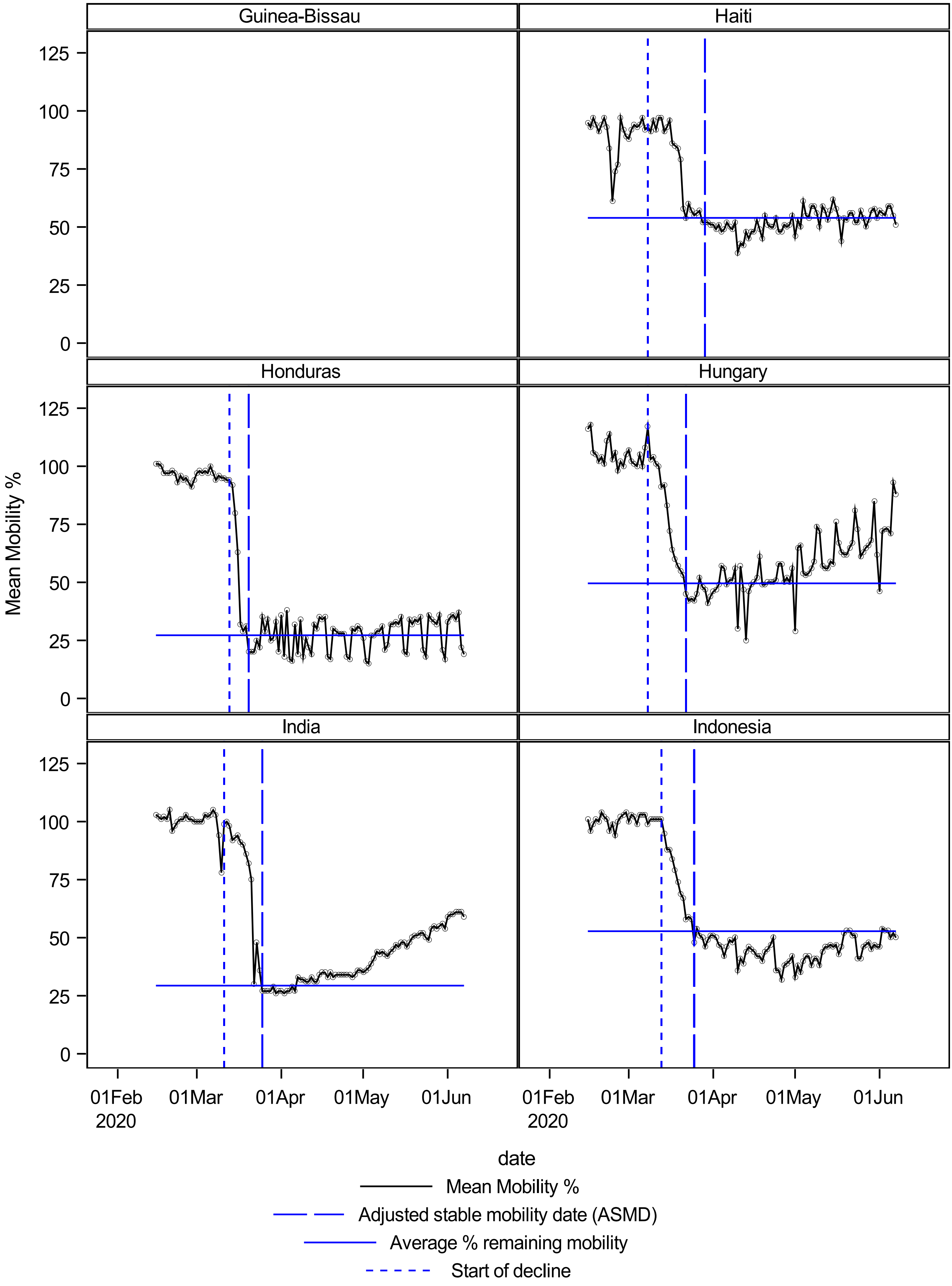

Supplementary Figures 3 Google TS mobility profiles per country

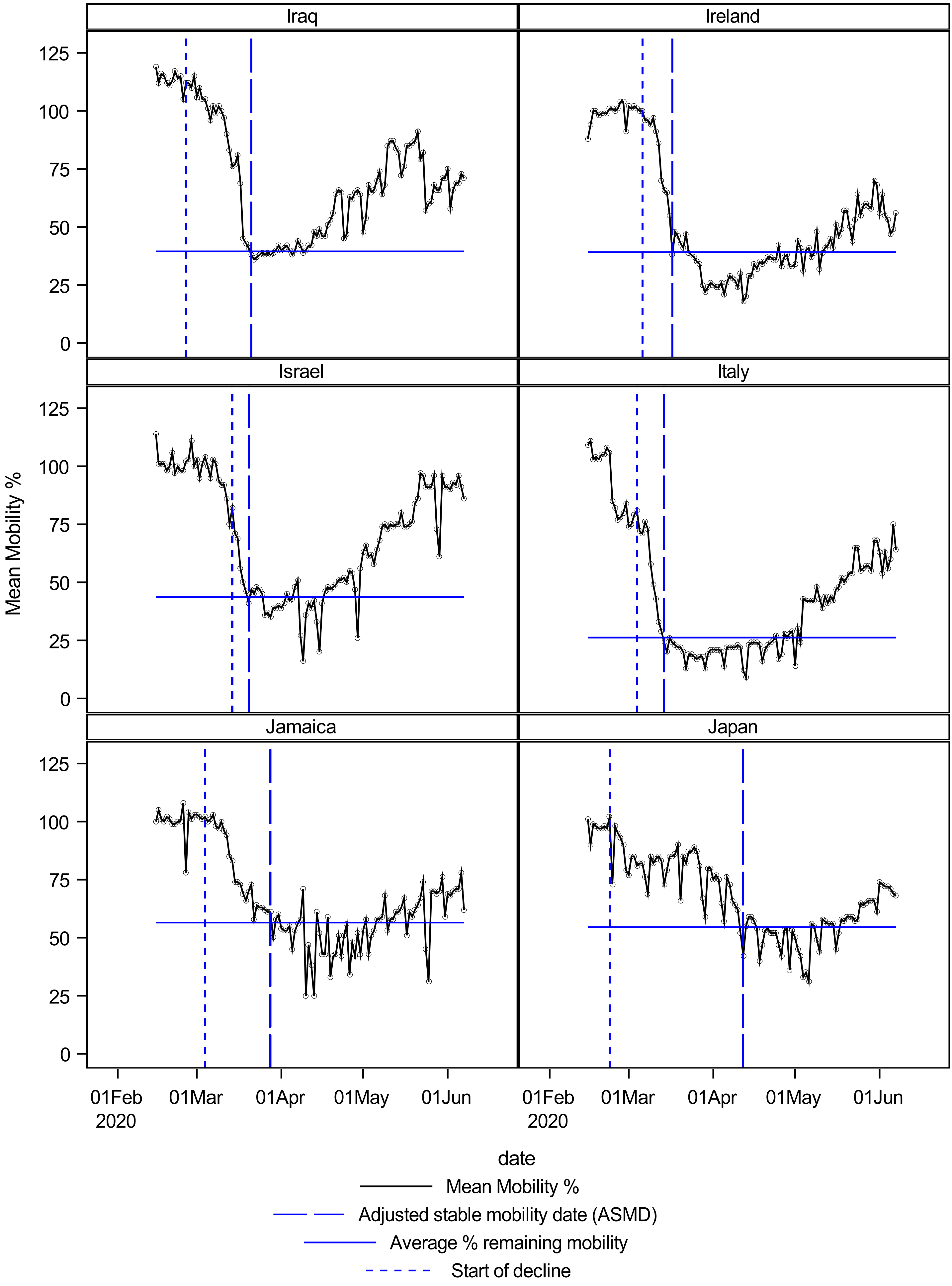

Supplementary Figures 3 Google TS mobility profiles per country

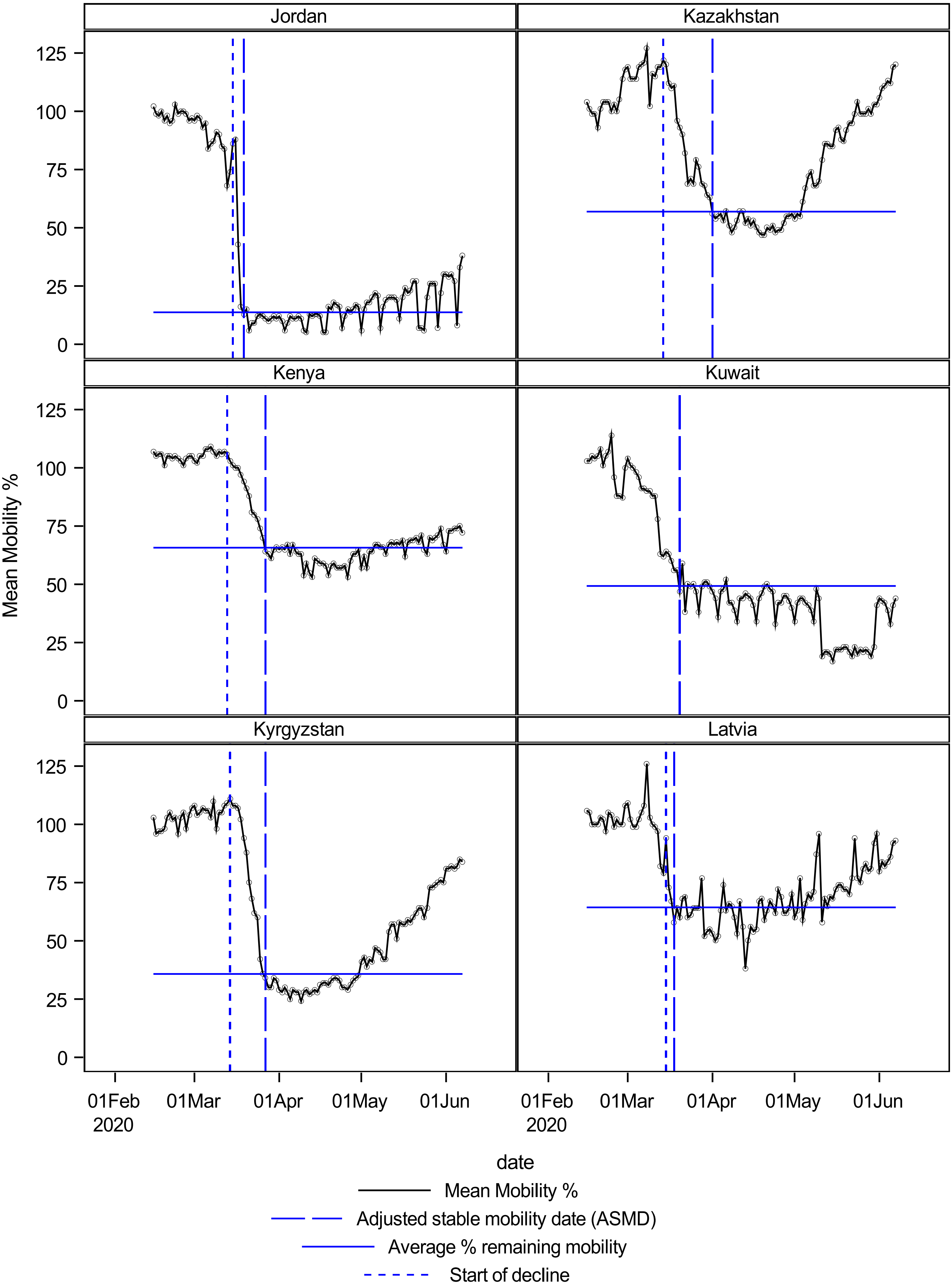

Supplementary Figures 3 Google TS mobility profiles per country

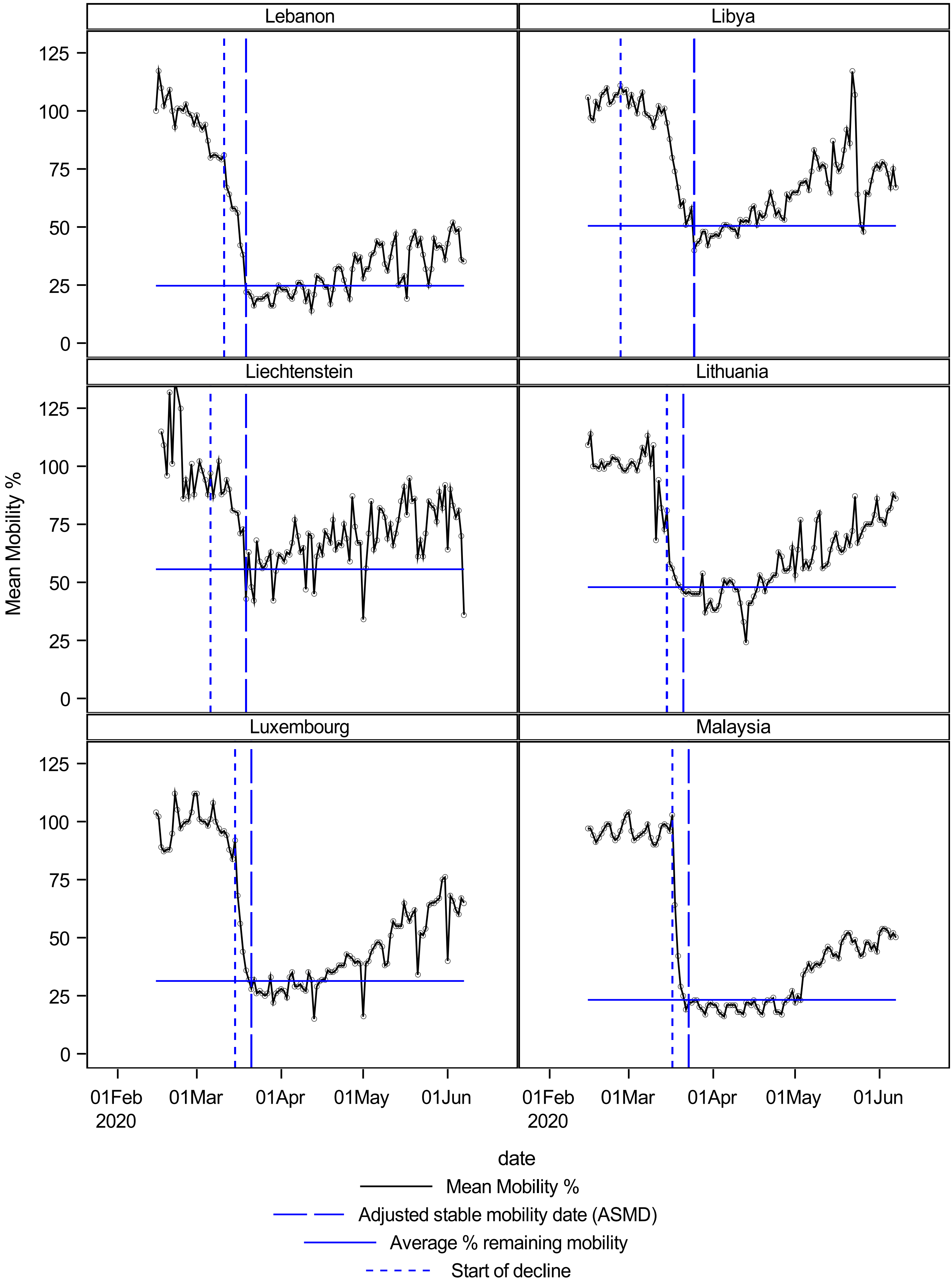

Supplementary Figures 3 Google TS mobility profiles per country

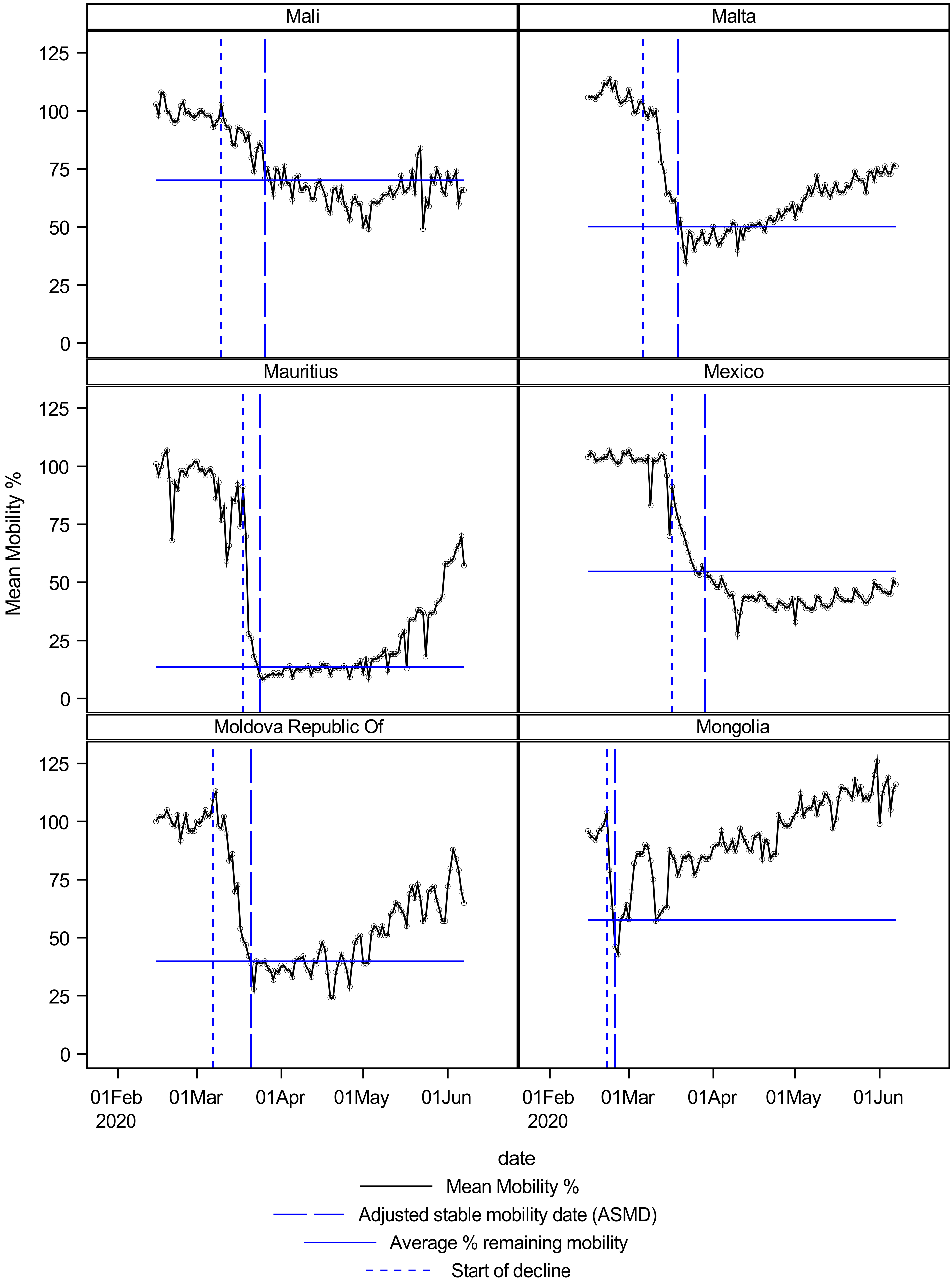

Supplementary Figures 3 Google TS mobility profiles per country

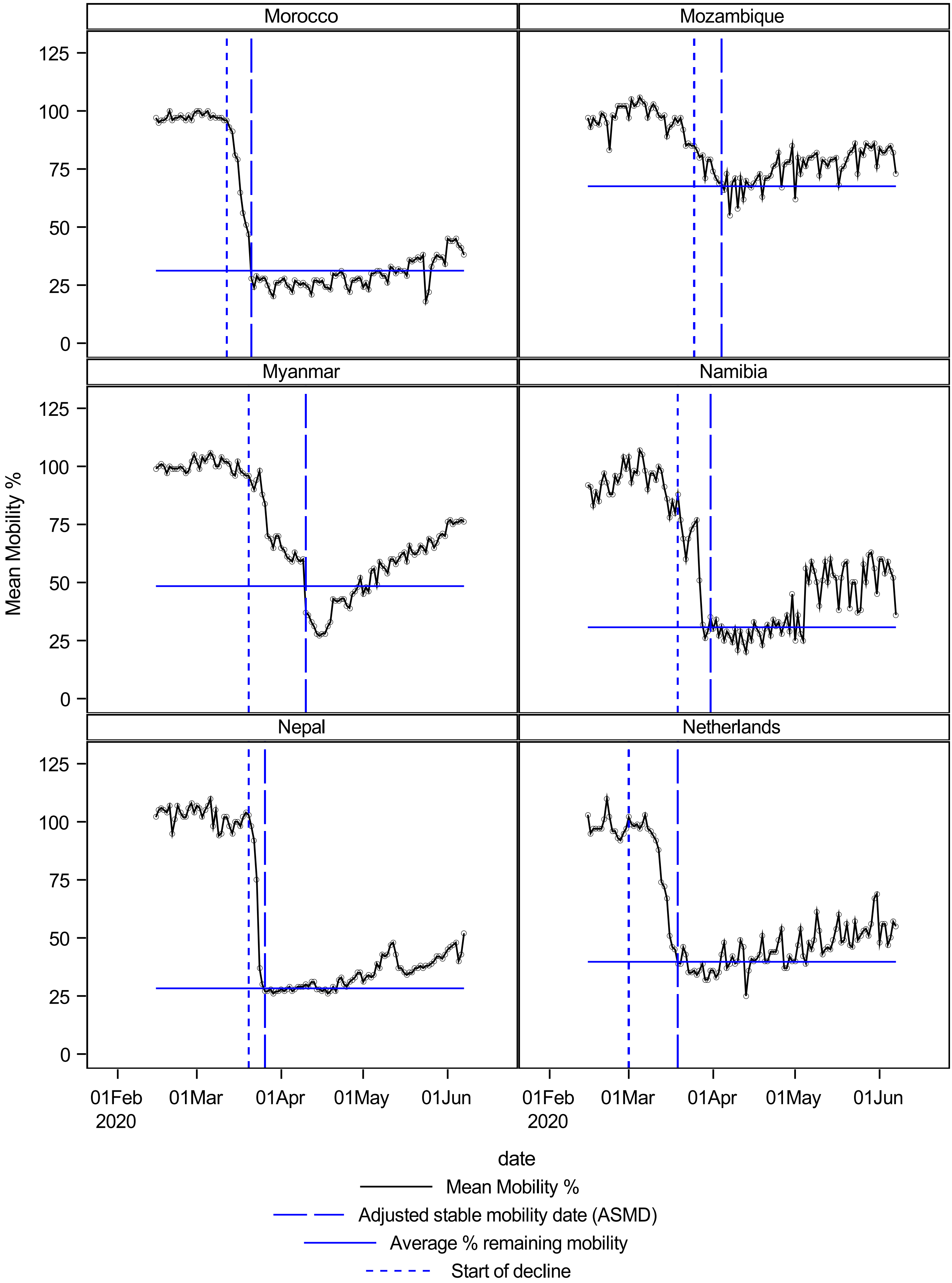

Supplementary Figures 3 Google TS mobility profiles per country

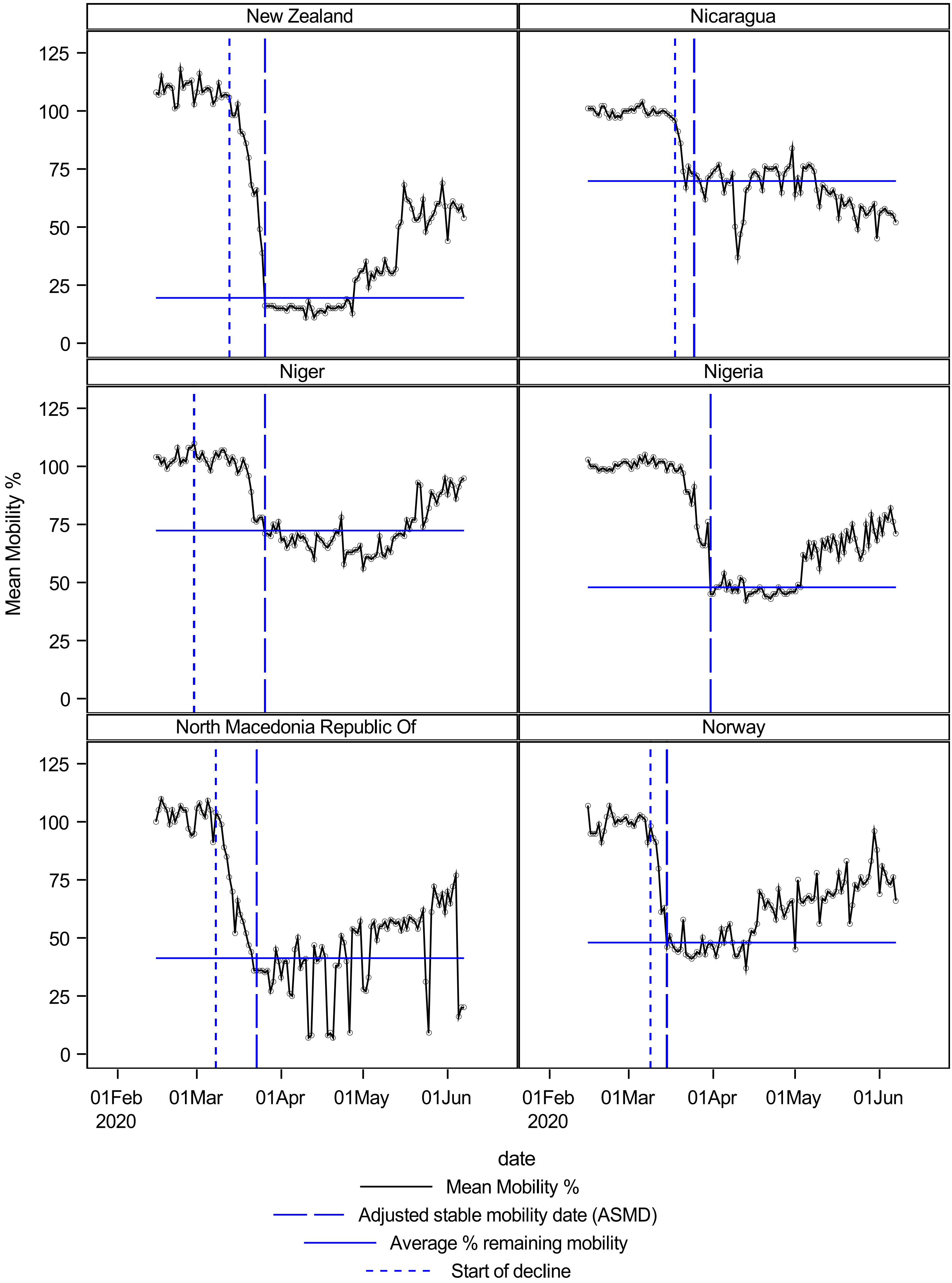

Supplementary Figures 3 Google TS mobility profiles per country

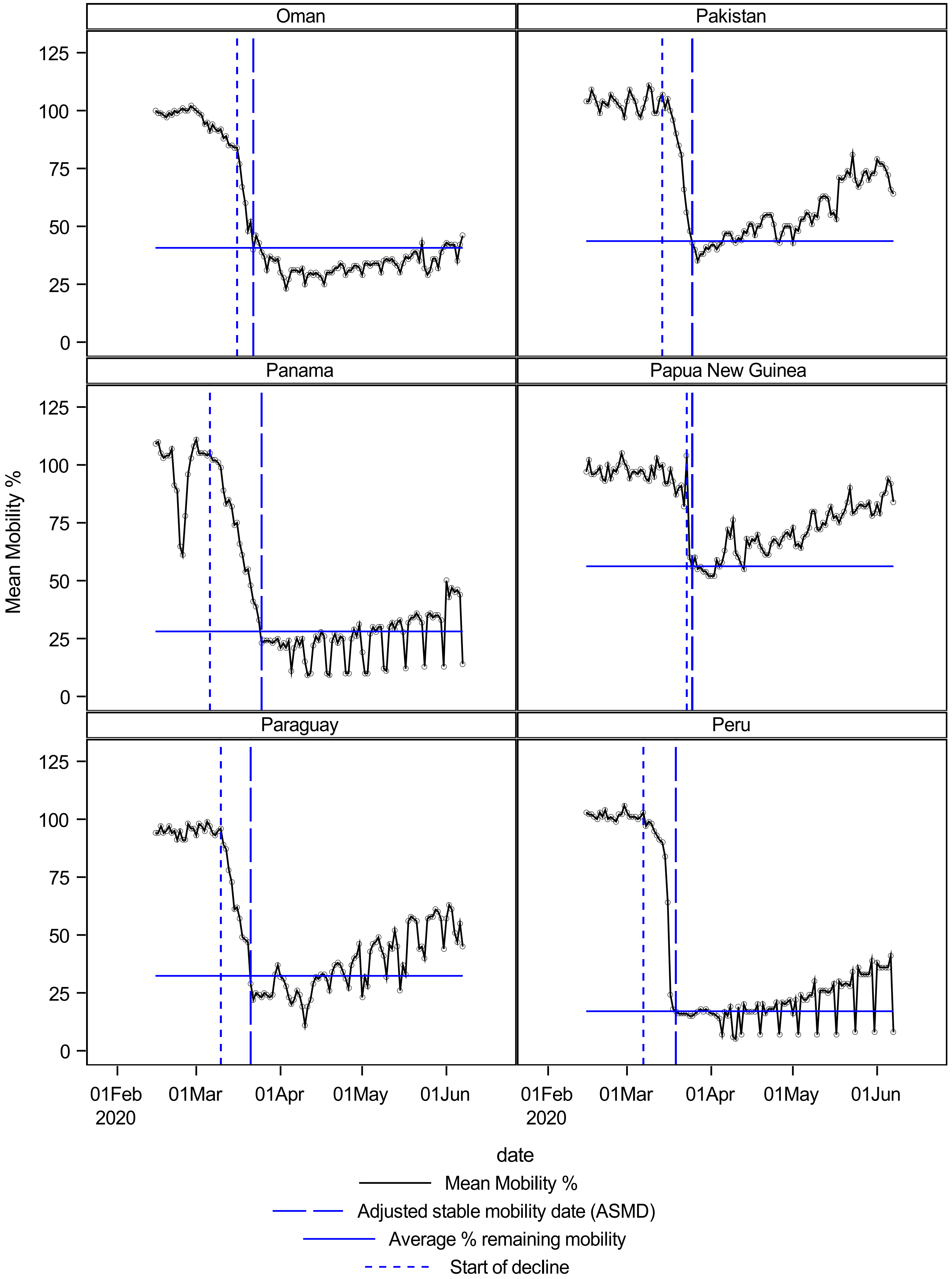

Supplementary Figures 3 Google TS mobility profiles per country

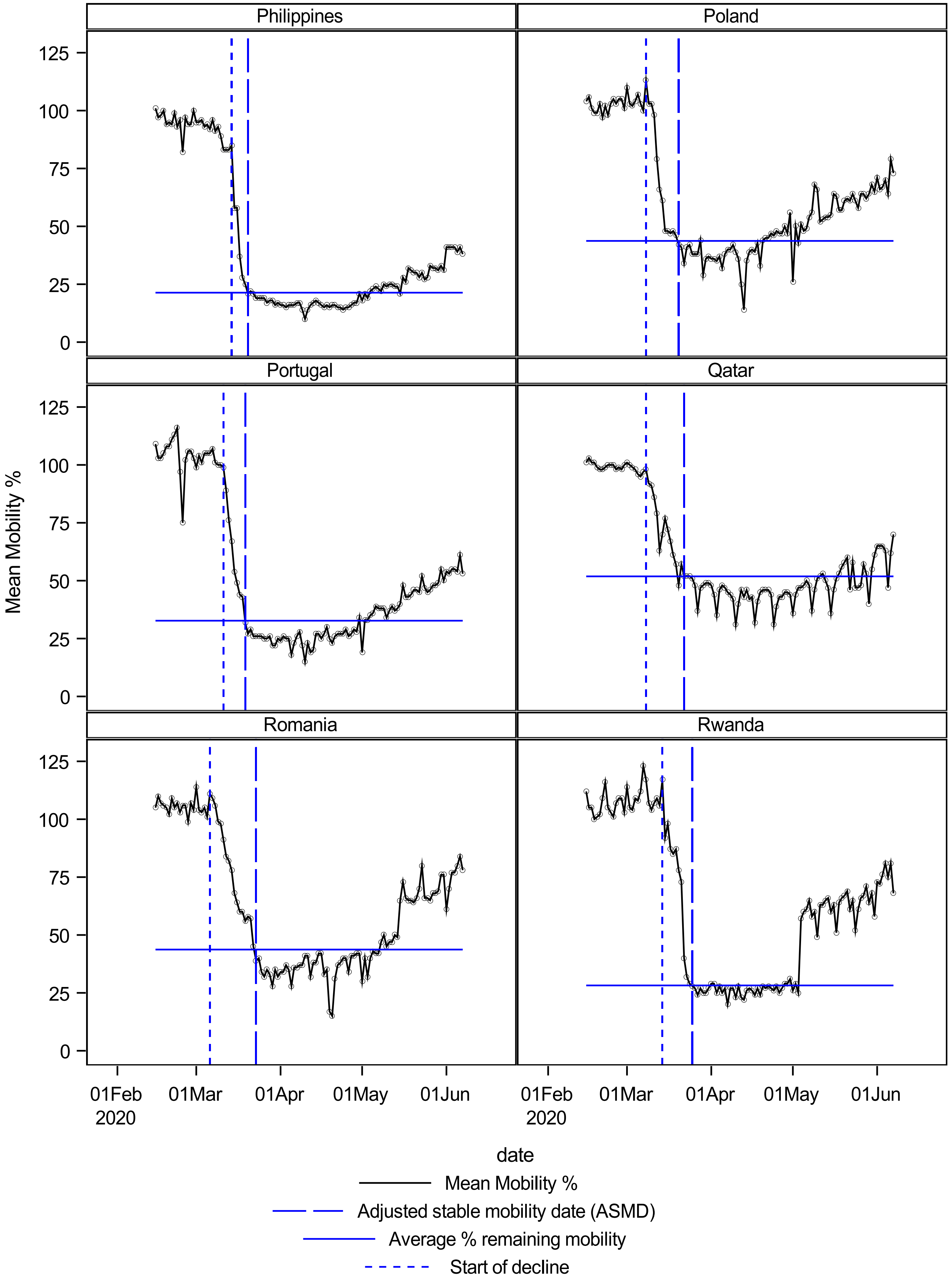

Supplementary Figures 3 Google TS mobility profiles per country

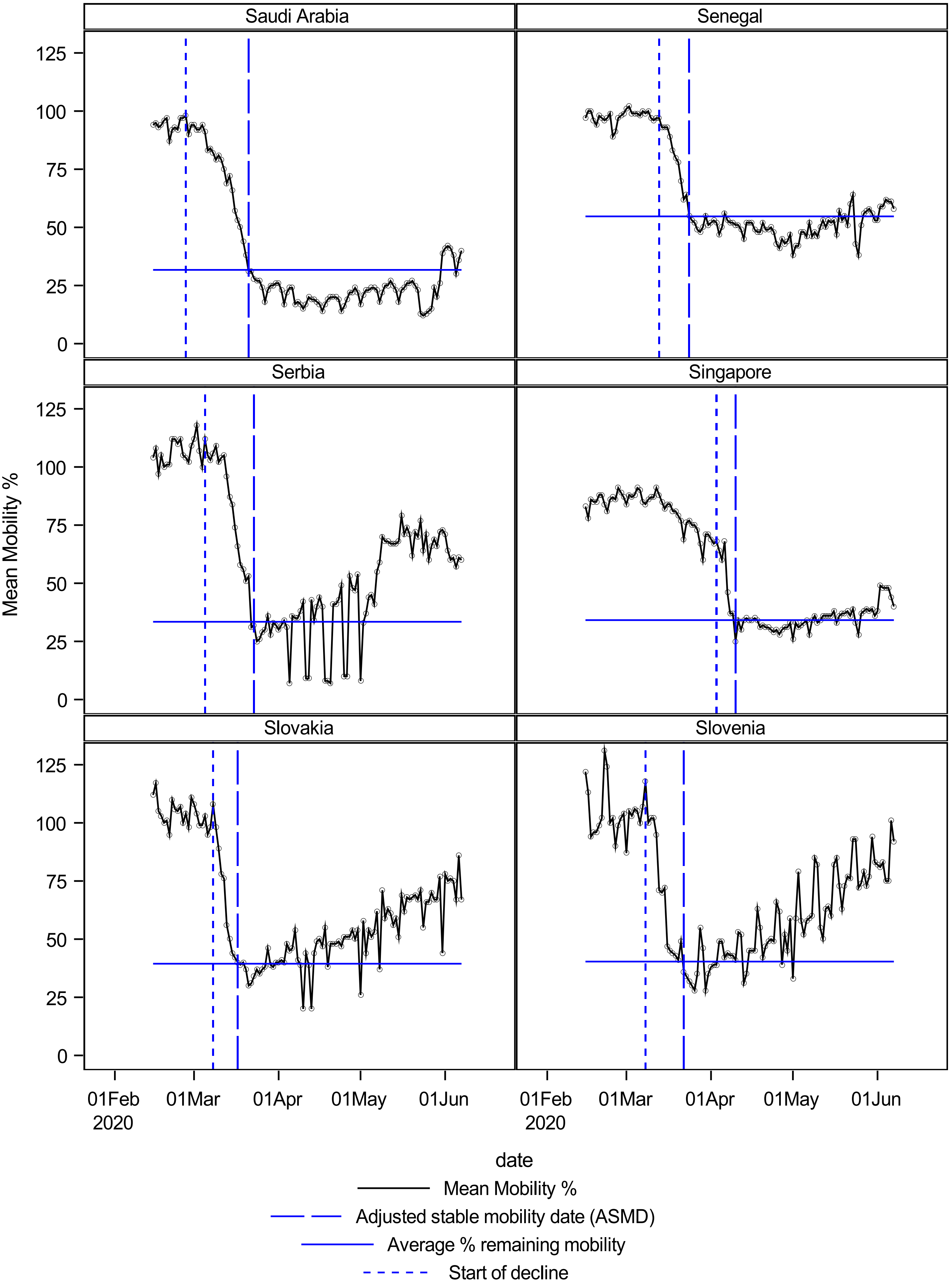

Supplementary Figures 3 Google TS mobility profiles per country

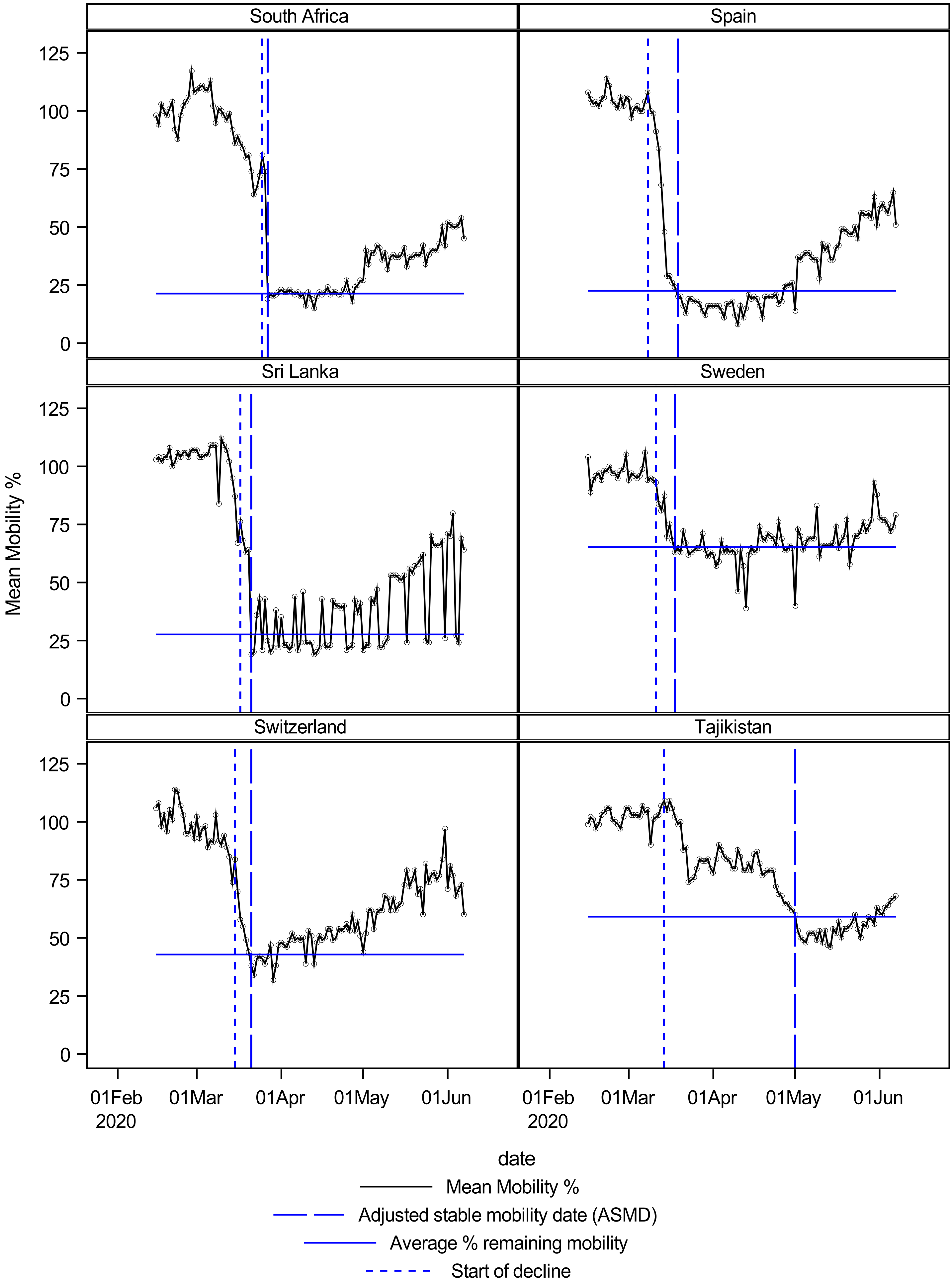

Supplementary Figures 3 Google TS mobility profiles per country

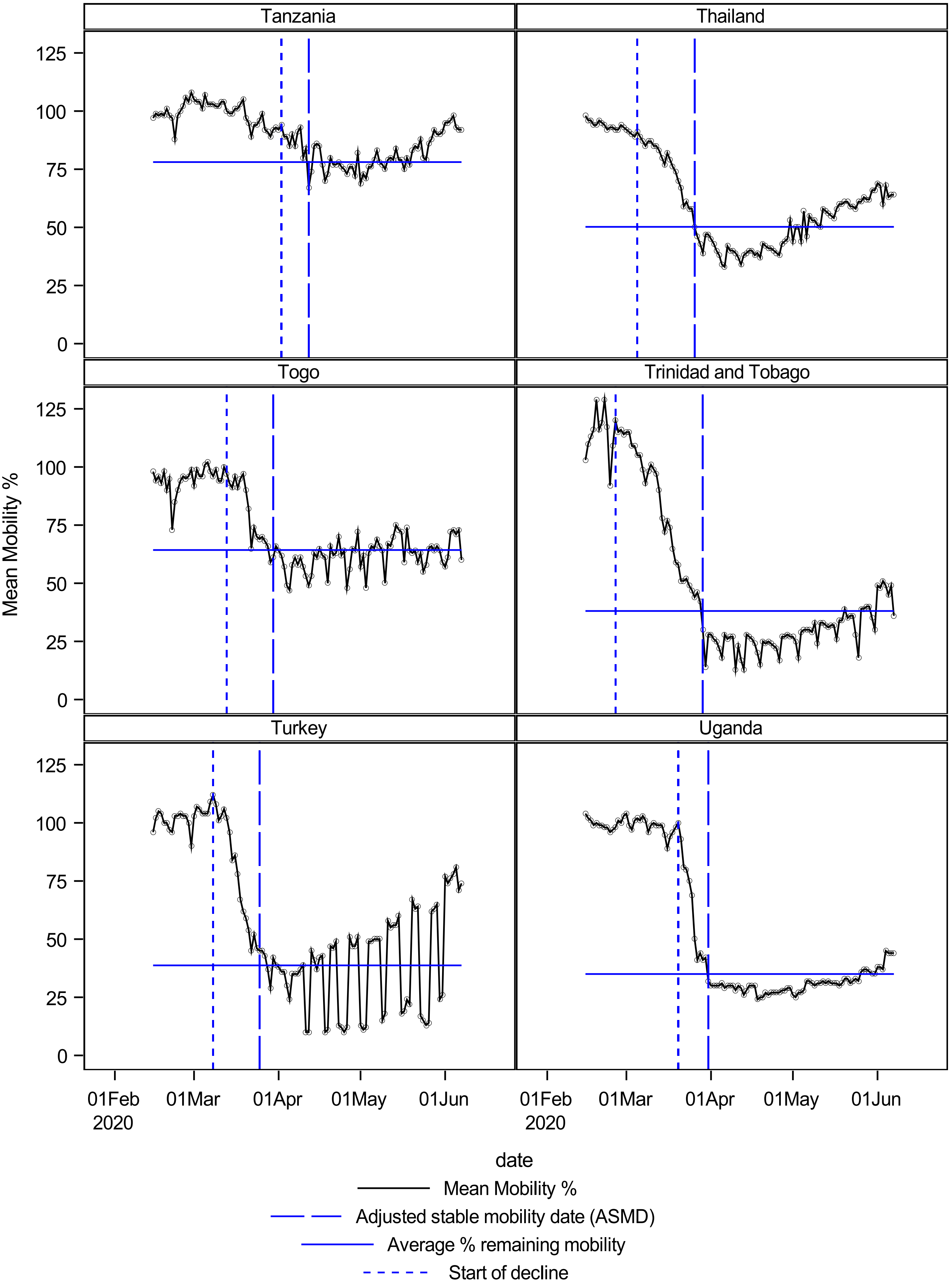

Supplementary Figures 3 Google TS mobility profiles per country

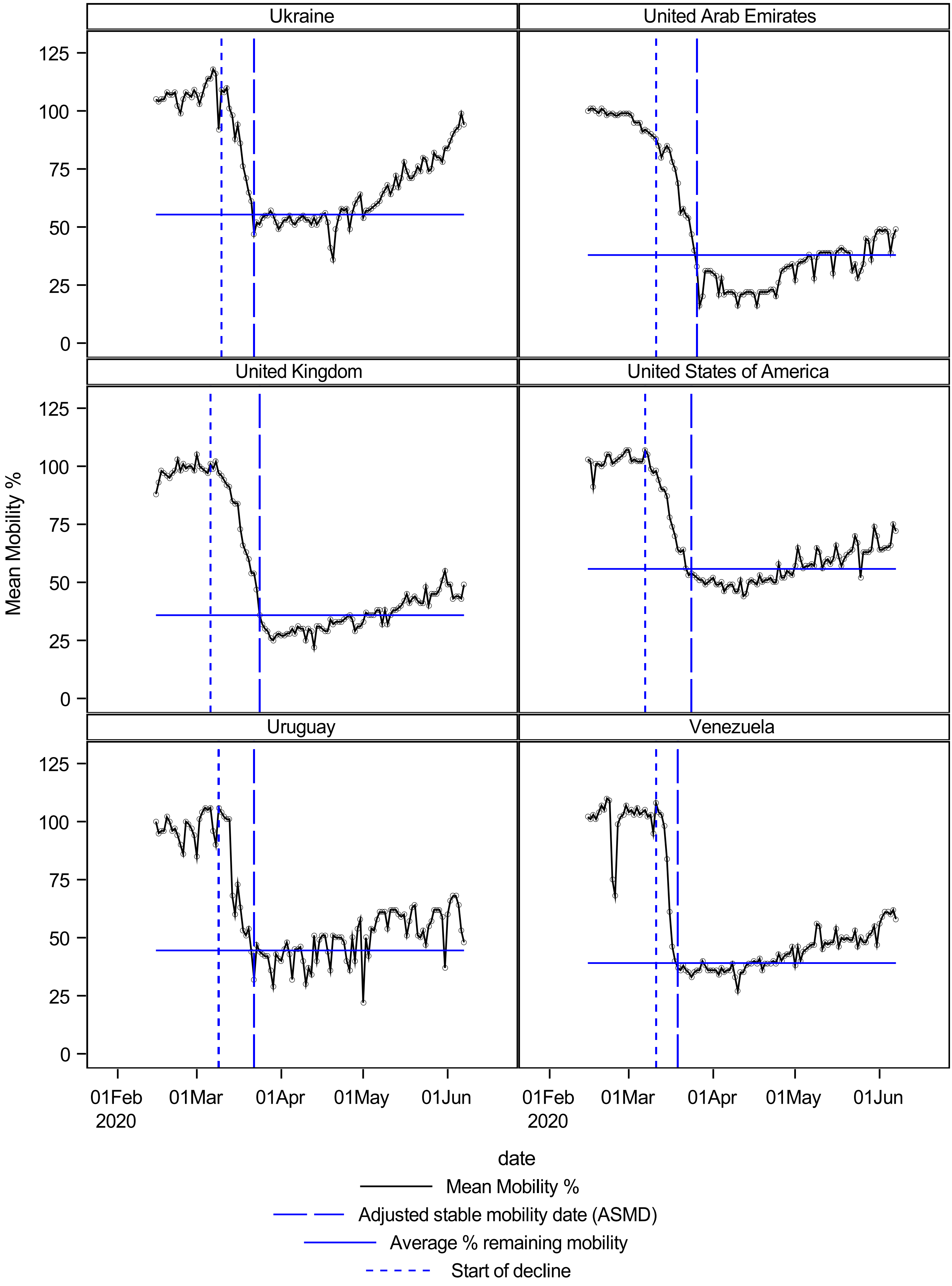

Supplementary Figures 3 Google TS mobility profiles per country

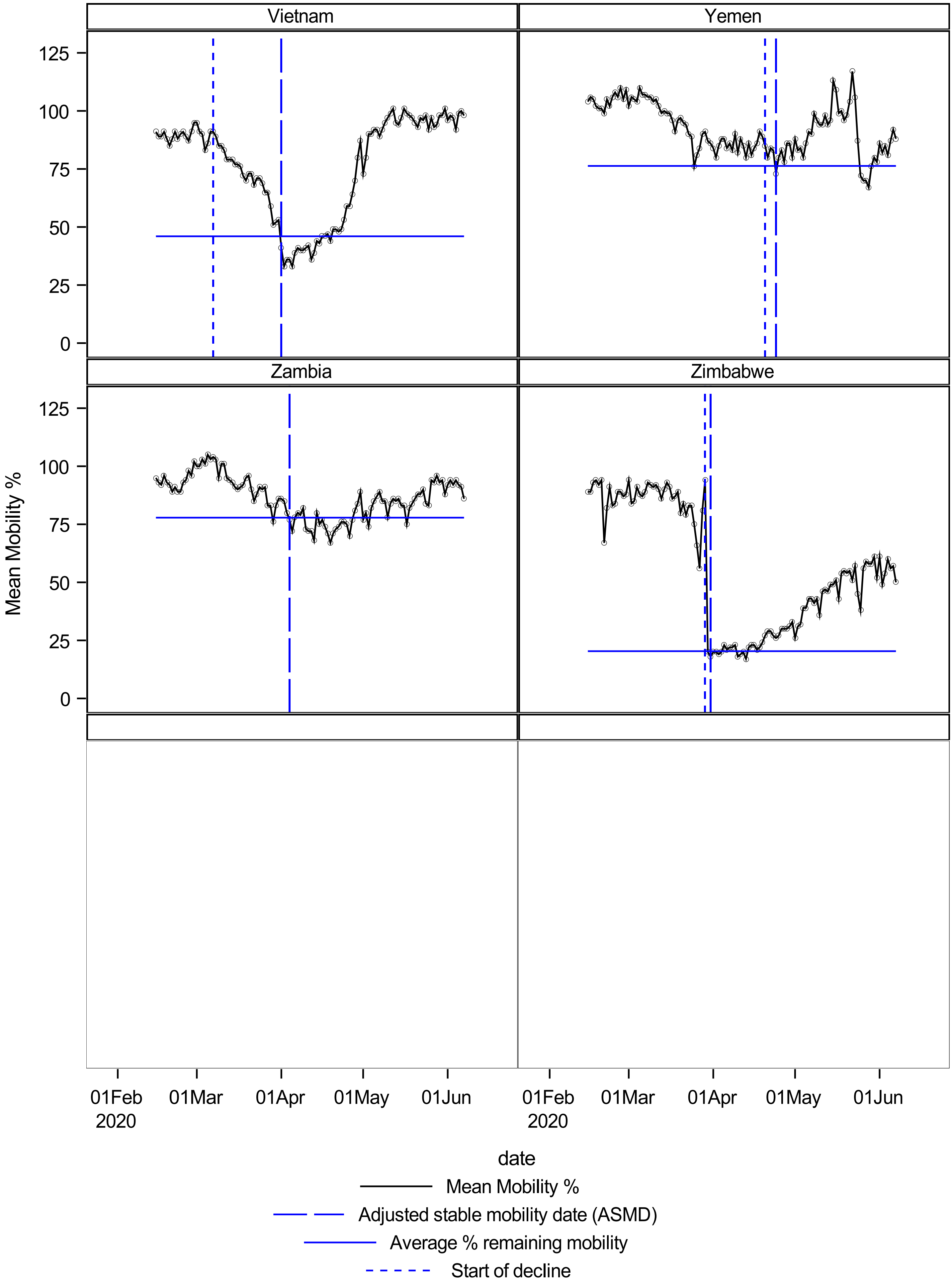

Supplement: Supplementary file 1 — Supplementary Information [file 41746_2021_451_MOESM1_ESM.pdf]
