# Supplementary material for: Measuring the effect of Non-Pharmaceutical Interventions (NPIs) on mobility during the COVID-19 pandemic using global mobility data
Source: NPJ Digit Med. 2021 May 13;4:81. doi: 10.1038/s41746-021-00451-2 (PMC8119480; doi:10.1038/s41746-021-00451-2)
Supplement: Supplementary file 2 — Reporting Summary [file 41746_2021_451_MOESM2_ESM.pdf]

nature research

Corresponding author(s): Berber T Snoeijer
Mariska Burger
Shaoxiong Sun
Richard JB Dobson
Amos A Folarin

Last updated by author(s): 24/03/2021

Reporting Summary

Nature Research wishes to improve the reproducibility of the work that we publish. This form provides structure for consistency and transparency in reporting. For further information on Nature Research policies, see our Editorial Policies and the Editorial Policy Checklist.

Statistics

For all statistical analyses, confirm that the following items are present in the figure legend, table legend, main text, or Methods section.

n/a	Confirmed	
☐	☒	The exact sample size (*n*) for each experimental group/condition, given as a discrete number and unit of measurement
☒	☐	A statement on whether measurements were taken from distinct samples or whether the same sample was measured repeatedly
☐	☒	The statistical test(s) used AND whether they are one- or two-sided<br>*Only common tests should be described solely by name; describe more complex techniques in the Methods section.*
☐	☒	A description of all covariates tested
☐	☒	A description of any assumptions or corrections, such as tests of normality and adjustment for multiple comparisons
☐	☒	A full description of the statistical parameters including central tendency (e.g. means) or other basic estimates (e.g. regression coefficient) AND variation (e.g. standard deviation) or associated estimates of uncertainty (e.g. confidence intervals)
☐	☒	For null hypothesis testing, the test statistic (e.g. *F*, *t*, *r*) with confidence intervals, effect sizes, degrees of freedom and *P* value noted<br>*Give P values as exact values whenever suitable.*
☒	☐	For Bayesian analysis, information on the choice of priors and Markov chain Monte Carlo settings
☒	☐	For hierarchical and complex designs, identification of the appropriate level for tests and full reporting of outcomes
☐	☒	Estimates of effect sizes (e.g. Cohen's *d*, Pearson's *r*), indicating how they were calculated

Our web collection on statistics for biologists contains articles on many of the points above.

Software and code

Policy information about availability of computer code

Data collection	Data used were open source and available from<br>Apple data: https://www.apple.com/covid19/mobility accessed on 14 June 2020<br>Google data: https://www.google.com/covid19/mobility/ accessed on 7 June 2020<br>non-pharmaceutical interventions database: https://data.humdata.org/dataset/acaps-covid19-government-measures-dataset accessed on 14 June 2020<br>The downloaded data used and derived paramters are available via https://github.com/MariskaBurger/Covid-19-analysis-results/tree/master.
Data analysis	All analyses were performed using SAS Studio 3.7 Enterprise Edition. The used SAS code is available via https://github.com/MariskaBurger/Covid-19-analysis-results/tree/master.

For manuscripts utilizing custom algorithms or software that are central to the research but not yet described in published literature, software must be made available to editors and reviewers. We strongly encourage code deposition in a community repository (e.g. GitHub). See the Nature Research guidelines for submitting code & software for further information.

Data

Policy information about availability of data

All manuscripts must include a data availability statement. This statement should provide the following information, where applicable:

- Accession codes, unique identifiers, or web links for publicly available datasets
- A list of figures that have associated raw data
- A description of any restrictions on data availability

For the analyses described in this article we have used freely available data sources from Apple, Google and ACAPs. The corresponding used data can be

April 2020

downloaded via their respective websites (See Data collection). The downloads used for the described analyses and datasets with derived parameters are available via https://github.com/MariskaBurger/Covid-19-analysis-results/tree/master.

Field-specific reporting

Please select the one below that is the best fit for your research. If you are not sure, read the appropriate sections before making your selection.

☐ Life sciences ☒ Behavioural & social sciences ☐ Ecological, evolutionary & environmental sciences

For a reference copy of the document with all sections, see nature.com/documents/nr-reporting-summary-flat.pdf

Behavioural & social sciences study design

All studies must disclose on these points even when the disclosure is negative.

Study description	A quantitative study measuring the effect of non-pharmaceutical interventions issued by governments on global mobility during the COVID-19 epidemic using global mobilty data made available by Apple and Google.
Research sample	All countries for which Apple and/or Google data were available and for which the governmental interventions were listed. For apple data the Sample size was 59 countries. For Apple mobility data the sample size was 124 countries.
Sampling strategy	All countries for which reliable data both from the mobility source (Apple or Google) as the governmental interventions database database ACAPS-COVID-19 (https://data.humdata.org/dataset/acaps-covid19-government-measures-dataset) were available
Data collection	Apple data: https://www.apple.com/covid19/mobility accessed on 14 June 2020<br>Google data: https://www.google.com/covid19/mobility/ accessed on 7 June 2020<br>non-pharmaceutical interventions database: https://data.humdata.org/dataset/acaps-covid19-government-measures-dataset The data in these data sources are incrementally collected and therefore not fixed. The data sources were accessed and data was downloaded on 14 June 2020. The corresponding datasets used are available via https://github.com/MariskaBurger/Covid-19-analysis-results/tree/master
Timing	Observation period Apple data: 13 januari 2020 - 14 June 2020<br>Observation period Google data: 15 february 2020 - 7 June 2020<br>Observation period ACAPs data: january 2020 - 14 June 2020
Data exclusions	Countries for which we were unable to accurately derive the two primary endpoints were excluded from the analysis.<br>For the Apple data for the analysis of the speed of the effect of the NPIs South-Africa was excluded as an extreme outlier.<br>For the Google RAR data for the analysis of the magnitude of the effect of the NPIs the following countries were excluded: Jamaica, Mongolia, Togo, Fiji and Yemen. For the Google TS data for the analysis of the magnitude of the effect of the NPIs the following countries were excluded: Mongolia, Yemen, Japan and Guinea Bissau.
Non-participation	not applicable
Randomization	not applicable

Reporting for specific materials, systems and methods

We require information from authors about some types of materials, experimental systems and methods used in many studies. Here, indicate whether each material, system or method listed is relevant to your study. If you are not sure if a list item applies to your research, read the appropriate section before selecting a response.

Materials & experimental systems

n/a	Involved in the study	
☒	☐	Antibodies
☒	☐	Eukaryotic cell lines
☒	☐	Palaeontology and archaeology
☒	☐	Animals and other organisms
☒	☐	Human research participants
☒	☐	Clinical data
☒	☐	Dual use research of concern

Methods

n/a	Involved in the study	
☒	☐	ChIP-seq
☒	☐	Flow cytometry
☒	☐	MRI-based neuroimaging
